# miR-330-5p targets SPRY2 to promote hepatocellular carcinoma progression via MAPK/ERK signaling

**DOI:** 10.1038/s41389-018-0097-8

**Published:** 2018-11-21

**Authors:** Shuai Xiao, Mengyuan Yang, Hao Yang, Ruimin Chang, Feng Fang, Lianyue Yang

**Affiliations:** 1 Liver Cancer Laboratory, Xiangya Hospital, Central South University, Changsha, Hunan China; 2 Department of Obstetrics and Gynecology, The Second Xiangya Hospital of Central South University, Changsha, Hunan China

## Abstract

MicroRNAs (miRNAs) have been identified as critical modulators of cell proliferation and growth, which are the major causes of cancer progression including hepatocellular carcinoma (HCC). Our previous miRNA microarray data have shown that miR-330-5p was always upregulated in HCC. However, the accurate role of miR-330-5p in HCC is still uncertain. Here, we report that miR-330-5p expression is upregulated in HCC tissues and cell lines, and is associated with tumor size, tumor nodule number, capsule formation and Tumor Node Metastasis (TNM) stage in HCC patients. Overexpression of miR-330-5p promotes proliferation and growth of HCC cells in vitro and in vivo, while miR-330-5p knockdown has the inverse effect. Moreover, using miRNA databases and dual luciferase report assay, we find miR-330-5p directly binds to the 3′-untranslated region (3′-UTR) of Sprouty2 (SPRY2). Then we find the novel biofunctional role of SPRY2 inactivation in promoting HCC progression. Finally, we confirm that miR-330-5p suppresses SPRY2 to promote proliferation via mitogen-activated protein kinases (MAPK)/extracellular regulated kinase (ERK) signaling in HCC. Taken together, our findings demonstrate the critical role of miR-330-5p in promoting HCC progression via targeting SPRY2 to activate MAPK/ERK signaling, which may provide a novel and promising prognostic marker and therapeutic target for HCC.

## Introduction

Hepatocellular carcinoma (HCC) is one of the most common and lethal malignancies in the world, which accounts for about 50% of the total number of cases and deaths each year in China^1^. Although surgical and systemic treatments for HCC have improved greatly in the past decade, the long-term survival is still poor due to the rapid cancer progression^2^. Cancer cell uncontrolled proliferation is one of the fundamental factors for HCC progression^3^. Evidence have shown that various growth factors, PTEN, p53, and etc. are responsible for HCC proliferation, and a series of targeted drugs such as sorafenib, APR-246, AD80 and so on are developed accordingly^4^. However, the intervention effect of these drugs is still unsatisfactory due to the precise regulatory mechanisms of HCC proliferation remain poorly understood. Therefore, there is an emergent need to reveal the potential mechanism of HCC proliferation and develop the new effective molecular targeted therapeutic strategy for HCC progression.

MicroRNAs (miRNAs) are a class of small noncoding RNAs that negatively modulate gene expression, which attract widespread attention and become a hotspot in cancer research^5^. More importantly, miRNA expression profiles have been successfully used to classify different tumor stages or subsets with distinct molecular pathology characteristic^6^. Our previous study has found a specific HCC subtype named solitary large HCC (SLHCC), which was one single lesion with a diameter over 5.0 cm and grew expansively, with aintact capsule or pseudo-capsule. The long-term survival of SLHCC was significantly longer than nodular HCC (NHCC, node number more than 1) and similar with small HCC (SHCC, 1 node, diameter≤ 5.0 cm)^7,8^. Accordingly, our previous genomic analysis revealed that the three HCC subtypes had distinct gene expression profiles, such as EGFL7, WAVE2, and VMP1 which could significantly affect tumor growth and metastasis^7–11^. These findings suggested that these HCC subtypes might have different inherent molecular characteristics, as well as miRNAs characteristic patterns. To identify this, we have detected the miRNA expression patterns in HCC by miRNA array analysis in the previous study^12^. Results showed some miRNAs had remarkably different expression levels among the HCC subtypes, such as miR-140-5p, miR-188-5p, miR-331-3p, etc., and these miRNAs had a strong effect on HCC progression and prognosis^12–14^.

Besides these reported miRNAs, we also found miR-330-5p expression level was upregulated in HCC, and the expression level in NHCC was significantly higher than SHCC and SLHCC in the miRNA array. miR-330-5p is the mature sequence of miR-330, and increasing studies reported that miR-330 played an important role in tumor progression such as glioblastoma, prostate cancer, as well as HCC^15–17^. However, there is still no report about the role of miR-330-5p in HCC now. Given the importance of miR-330 in cancer progression and miR-330-5p expression pattern in HCC, thereby there is a strong demand to explore the role of miR-330-5p in HCC progression.

In this study, we provide the first evidence that miR-330-5p is upregulated in HCC and its high expression level related to poor survival of HCC patients. miR-330-5p overexpression promotes HCC proliferation and growth through targeting Sprouty2 (SPRY2) mediated mitogen-activated protein kinase (MAPK)/extracellular regulated kinase (ERK) signaling. These results suggest that miR-330-5p may be a valuable prognostic biomarker and potential therapeutic target for HCC.

## Results

### miR-330-5p expression was significantly upregulated in HCC

**Fig. 1:**
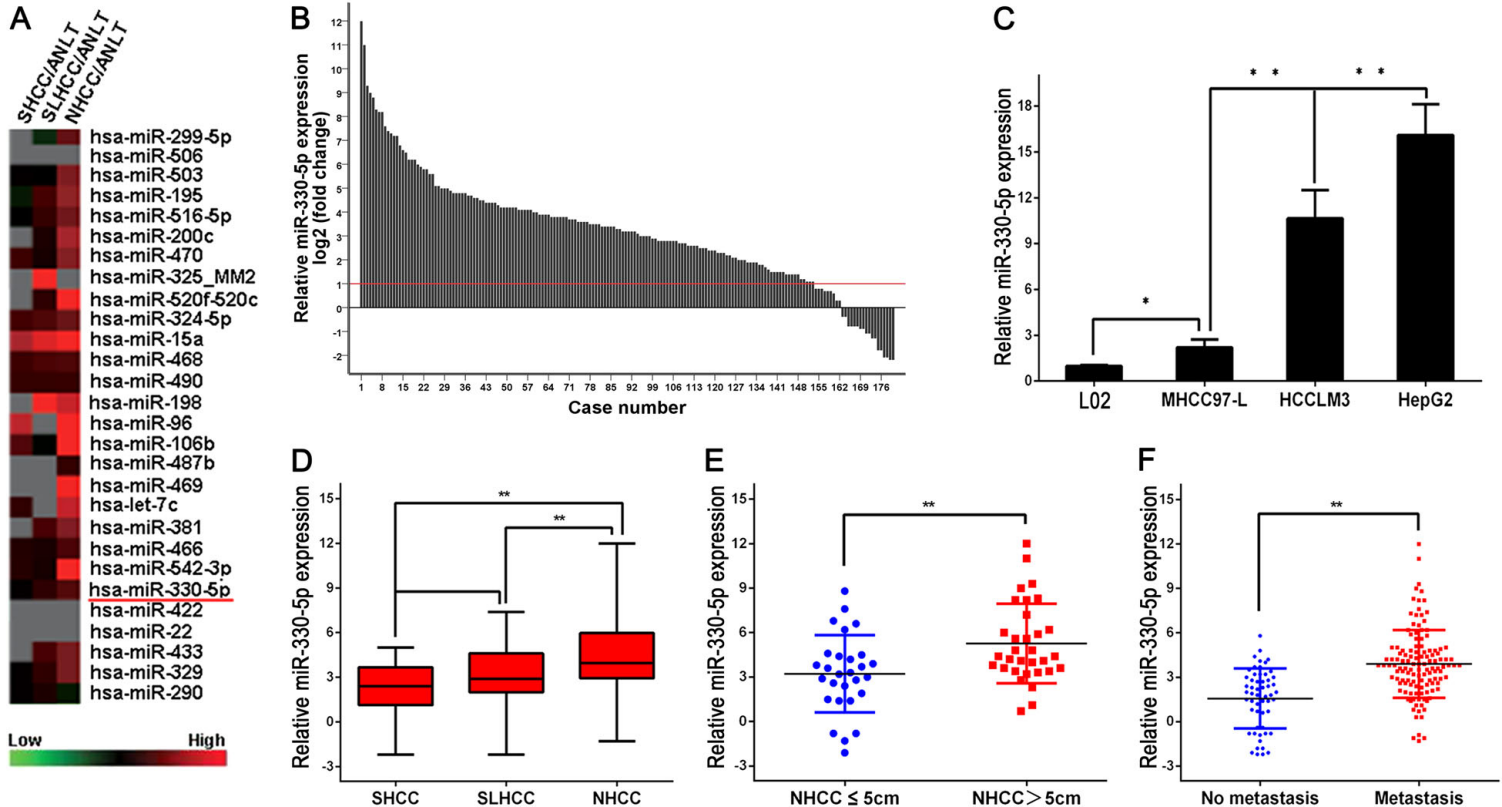
miR-330-5p expression was upregulated in HCC. **a** Cluster analysis of miRNAs expression profiles of SHCC, SLHCC, and NHCC versus ANLTs. High expression was indicated in red, whereas low expression was coded in green. **b** Expression of miR-330-5p in 180 pairs of HCC tissues and the corresponding ANLTs. Expression levels of miR-330-5p were normalized to the corresponding levels of U6 RNA. Data were analyzed as log^2^ fold change (2^-[ΔCt(HCC)-ΔCt(ANLT)]^). **c** miR-330-5p expression in liver cell line L02 and HCC cell lines (MHCC97-L, HCCLM3 and HepG2). **d** miR-330-5p expression in NHCC was higher than SHCC and SLHCC. (E) miR-330-5p expression in NHCC with tumor size > 5 cm was higher than that ≤5 cm. **f** miR-330-5p expression in HCC with metastasis was higher than that without metastasis. *P value < 0.05; **P value < 0.01

Our previous miRNA expression microarray analysis showed that miR-330-5p was higher expressed in HCC compared to ANLT, especially step-increased in SHCC, SLHCC, and NHCC (Fig. 1a). We then performed real-time polymerase chain reaction (PCR) to detect miR-330-5p expression in 180 pairs of HCC (60 pairs of each HCC subtype) bulk tissues and corresponding ANLTs. Consistent with the microarray results, miR-330-5p expression was significantly up-regulated in HCC tissues compared to corresponding ANLTs (more than 2-fold [i.e., log_2_ (fold change) > 1]) (Fig. 1b). Then, we also detected miR-330-5p expression in liver cell lines L02 and HCC cell lines (MHCC97-L, HCCLM3, and HepG2) by real-time PCR. Results showed that miR-330-5p expression was markedly higher in HCC cell lines than liver cell line L02, and the expression level was the highest in HepG2 cells which had strong proliferation capacity, but the lowest in MHCC97-L cells whose proliferation capacity was relatively weak (Fig. 1c). Subsequently, we made a comparison of miR-330-5p expression levels in different HCC subtypes, the results showed that miR-330-5p expression in NHCC was significantly higher than that in SHCC and SLHCC (*P* < 0.01, respectively) (Fig. 1d). Because miR-330-5p expression in SHCC was similar to SLHCC (Fig. 1d), which indicated size was not a critical factor for solitary tumor. Next, we compared miR-330-5p expression in NHCC with different sizes, results showed that miR-330-5p expression in group of NHCC with tumor size > 5.0 cm was obviously higher than that ≤ 5.0 cm (*P* < 0.01, Fig. 1e). Moreover, we also found miR-330-5p expression in HCC with metastasis was higher than that without metastasis (*P* < 0.01, Fig. 1f). These results showed that miR-330-5p expression was upregulated in HCC tissues and cell lines, especially in NHCC, NHCC with bigger tumor size and HCC with metastasis, which implied that miR-330-5p expression might be associated with HCC progression and prognosis.

### miR-330-5p high expression indicated poor prognosis of HCC

**Fig. 2:**
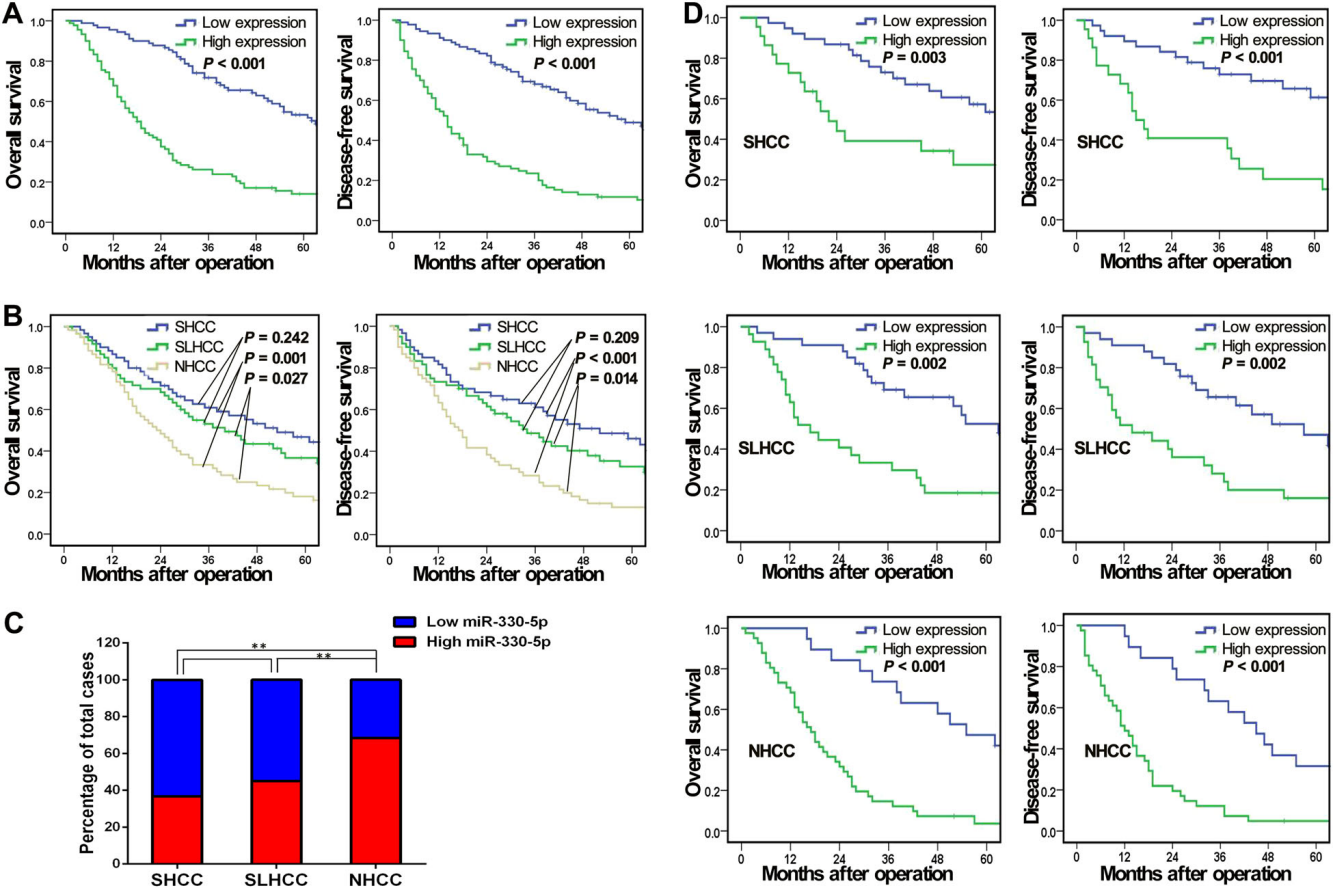
miR-330-5p high expression indicated poor prognosis of HCC. **a** High miR-330-5p expression associated with shorter OS and DFS of HCC. **b** The OS and DFS of NHCC were shorter than SHCC and SLHCC, which had a similar OS and DFS. **c** NHCC had more percent of high miR-330-5p expression cases than SHCC and SLHCC, while SHCC and SLHCC had no significant difference. **d** The high miR-330-5p expression group had a poorer OS and DFS in NHCC, SHCC, and SLHCC by stratification analysis. *OS* overall survival, *DFS* disease-free survival. **P value < 0.01

To further explore the correlation of miR-330-5p expression with clinicopathologic features and long-term survival of HCC patients after hepatic resection (HR), the median relative expression level of miR-330-5p in all 180 HCC samples was chosen as the cut-off point for separating HCC with the low and high expression of miR-330-5p groups. Results showed that high miR-330-5p expression was significantly associated with tumor size, tumor nodule number, capsule formation, Tumor Node Metastasis (TNM) stage and Barcelona Clinic Liver Cancer (BCLC) stage in HCC (all *P* < 0.05, Table 1). Kaplan–Meier survival analysis revealed that the 1, 3, 5-year overall survival (OS) and disease-free survival (DFS) of low miR-330-5p expression group were 95.6, 71.7, 53.3%, and 91.1, 68.1, 48.9%, which were significantly better than that in high miR-330-5p expression group of 67.8, 26.1, 14.1% and 55.6, 23.6, 11.8% (*P* < 0.001, respectively, Fig. 2a). Further stratification analysis revealed that OS and DFS among the three HCC subtypes were different. NHCC had the poorer OS when compared with SHCC and SLHCC (*P* = 0.001 and *P* = 0.027) as well as in DFS (*P* < 0.001 and *P* = 0.014), but the OS (*P* = 0.242) and DFS (*P* = 0.209) were similar between SHCC and SLHCC (Fig. 2b). These results were consistent with our previous study about the outcome of the different HCC subtypes^7^. In this study, we also found NHCC group had more percent of high miR-330-5p expression cases than SHCC and SLHCC groups (all *P* < 0.01, Fig. 2c), while SHCC and SLHCC groups had no significant difference (*P* > 0.05, Fig. 2c), which was consistent with Fig. 1d. Stratification survival analysis also showed that HCCs with high miR-330-5p expression group had a poorer OS and DFS than that with low miR-330-5p expression in each of the three HCC subtypes (all *P* < 0.01, Fig. 2d). These data indicated that miR-330-5p expression was associated with poor prognosis of HCC and might be a stratification prognostic marker for HCC subtypes.

**Table 1:** Correlations between miR-330-5p expression level and clinicopathological variables of 180 cases of HCC

Clinicopathologic variables	*n*	miR-330-5p expression		*P* value
		Low (90)	High (90)	
Gender				
Male	131	69	62	
Female	49	21	28	0.241
Age(years)				
≤60	146	77	69	
>60	34	13	21	0.128
AFP, ng/mL				
<20	87	48	39	
≥20	93	42	51	0.180
Hepatitis B status				
Negative	16	10	6	
Positive	164	80	84	0.295
Cirrhosis				
Absence	51	29	22	
Presence	129	61	68	0.247
Liver function				
Child-Pugh A	161	79	82	
Child-Pugh B	19	11	8	0.467
Tumor size (cm)				
≤5	68	41	27	
>5	112	49	63	0.031
Tumor nodule number				
Solitary	113	64	49	
Multiple(≥2)	67	26	41	0.021
Capsule formation				
Presence	82	51	31	
Absence	98	39	59	0.003
Edmondson-Steiner grade				
I-II	93	51	42	
III-IV	87	39	48	0.180
Microvascular invasion				
Absence	84	38	46	
Presence	96	52	44	0.232
TNM stage				
Stage I	75	47	28	
Stage II-III	105	43	62	0.004
BCLC stage				
Stage 0-A	87	51	36	
Stage B-C	93	39	54	0.025

No patients with Child-Pugh C or TNM Stage IV were included

*AFP* alpha-fetoprotein, *HBsAg*, *TNM* tumor node metastasis, *BCLC* Barcelona Clinic Liver Cancer

Next, in order to assess the value of the miR-330-5p expression in predicting HCC prognosis, the Cox proportional hazards regression model was applied. The univariate analysis revealed that cirrhosis, tumor size, tumor nodule number, Edmondson-Steiner grade, microvascular invasion, TNM stage, BCLC stage, as well as miR-330-5p expression were significant for OS (all *P* < 0.05, Table 2). Next, multivariate analysis revealed that OS was significantly dependent on cirrhosis, microvascular invasion, BCLC stage, and miR-330-5p expression (all *P* < 0.05, Table 2). Further analysis of DFS demonstrated that cirrhosis, tumor nodule number, microvascular invasion, TNM stage, BCLC stage, and miR-330-5p expression were significant in the univariate analysis (all *P* < 0.05, Table 3), while only tumor nodule number, microvascular invasion, BCLC stage, and miR-330-5p expression were independent risk factors for DFS in multivariate analysis (all *P* < 0.05, Table 3). These results indicated that miR-330-5p expression was closely correlated with adverse clinicopathological features and shorter survival of HCC patients, and was a potential prognostic marker for HCC.

**Table 2:** The Cox regression analyses of overall survival (OS) and miR-330-5p expression level as well as clinicopathological parameters

Variables	*n*	Univariable analysis		Multivariable analysis	
		HR (95% CI)	*P*	HR (95% CI)	*P*
Gender					
Male	131	1			
Female	49	1.31 (0.43–2.27)	0.427	NA	
Age(years)					
≤60	146	1			
>60	34	1.21 (0.71–1.83)	0.548	NA	
AFP, ng/mL					
<20	87	1			
≥20	93	1.84 (0.54–3.23)	0.085	NS	
Hepatitis B status					
Negative	16	1			
Positive	164	1.53 (0.49–2.86)	0.143	NA	
Cirrhosis					
Absence	51	1		1	
Presence	129	3.42 (1.59–5.73)	<0.001	2.94 (1.36–4.82)	0.001
Liver function					
Child-Pugh A	161	1			
Child-Pugh B	19	1.93 (0.78–3.46)	0.072	NS	
Tumor size (cm)					
≤5	68	1			
>5	112	2.01 (1.06–3.73)	0.036	NS	
Tumor nodule number					
Solitary	113	1			
Multiple(≥2)	67	2.23 (1.17–3.92)	0.014	NS	
Capsule formation					
Presence	82	1			
Absence	98	1.62 (0.56–3.04)	0.183	NA	
Edmondson-Steiner grade					
I-II	93	1			
III-IV	87	1.79 (1.15–2.90)	0.042	NS	
Microvascular invasion					
Absence	84	1		1	
Presence	96	3.72 (1.63–6.51)	<0.001	2.85 (1.29–4.68)	0.002
TNM stage					
Stage I-II	75	1			
Stage III	105	2.62 (1.44–4.23)	0.009	NS	
BCLC stage					
Stage 0-A	87	1		1	
Stage B-C	93	2.91 (1.25–5.03)	<0.001	2.46 (1.18–4.05)	0.013
MiR-330-5p expression					
Low	90	1		1	
High	90	2.14 (1.18–3.25)	0.007	1.96 (1.07–3.24)	0.028

*HR* hazard risk ratio, *CI* confidence interval, *NA* not applicable, *NS* not significant

**Table 3:** The Cox regression analyses of disease-free survival (DFS) and miR-330-5p expression level as well as clinicopathological parameters

Variables	*n*	Univariable analysis		Multivariable analysis	
		HR (95% CI)	*P*	HR (95% CI)	*P*
Gender					
Male	131	1			
Female	49	1.17 (0.63–1.85)	0.736	NA	
Age(years)					
≤60	146	1			
>60	34	1.41 (0.74–2.31)	0.529	NA	
AFP, ng/mL					
<20	87	1			
≥20	93	1.75 (0.59–3.24)	0.092	NS	
Hepatitis B status					
Negative	16	1			
Positive	164	2.03 (0.87–3.45)	0.068	NS	
Liver function					
Child-Pugh A	51	1			
Child-Pugh B	129	1.88 (0.69–3.27)	0.081	NS	
Cirrhosis					
Absence	161	1			
Presence	19	1.95 (1.12–3.16)	0.034	NS	
Tumor size (cm)					
≤5	68	1			
>5	112	1.52 (0.48-2.79)	0.233	NA	
Tumor nodule number					
Solitary	113	1		1	
Multiple(≥2)	67	2.13 (1.26-3.43)	0.010	1.97 (1.15–3.04)	0.028
Capsule formation					
Presence	82	1			
Absence	98	1.79 (0.72-3.17)	0.069	NS	
Edmondson-Steiner grade					
I-II	93	1			
III-IV	87	1.44 (0.83-2.24)	0.325	NA	
Microvascular invasion					
Presence	84	1		1	
Absence	96	2.47 (1.22–4.57)	0.006	2.12 (1.15–3.82)	0.013
TNM stage					
Stage I-II	75	1			
Stage III	105	1.89 (1.07–3.21)	0.035	NS	
BCLC stage					
Stage 0-A	87	1		1	
Stage B-C	93	2.61 (1.34–4.74)	0.001	2.25 (1.16–3.92)	0.007
MiR-330-5p expression					
Low	90	1		1	
High	90	2.17 (1.21–3.31)	0.004	1.93(1.13–3.24)	0.019

### miR-330-5p promoted proliferation and growth of HCC

**Fig. 3:**
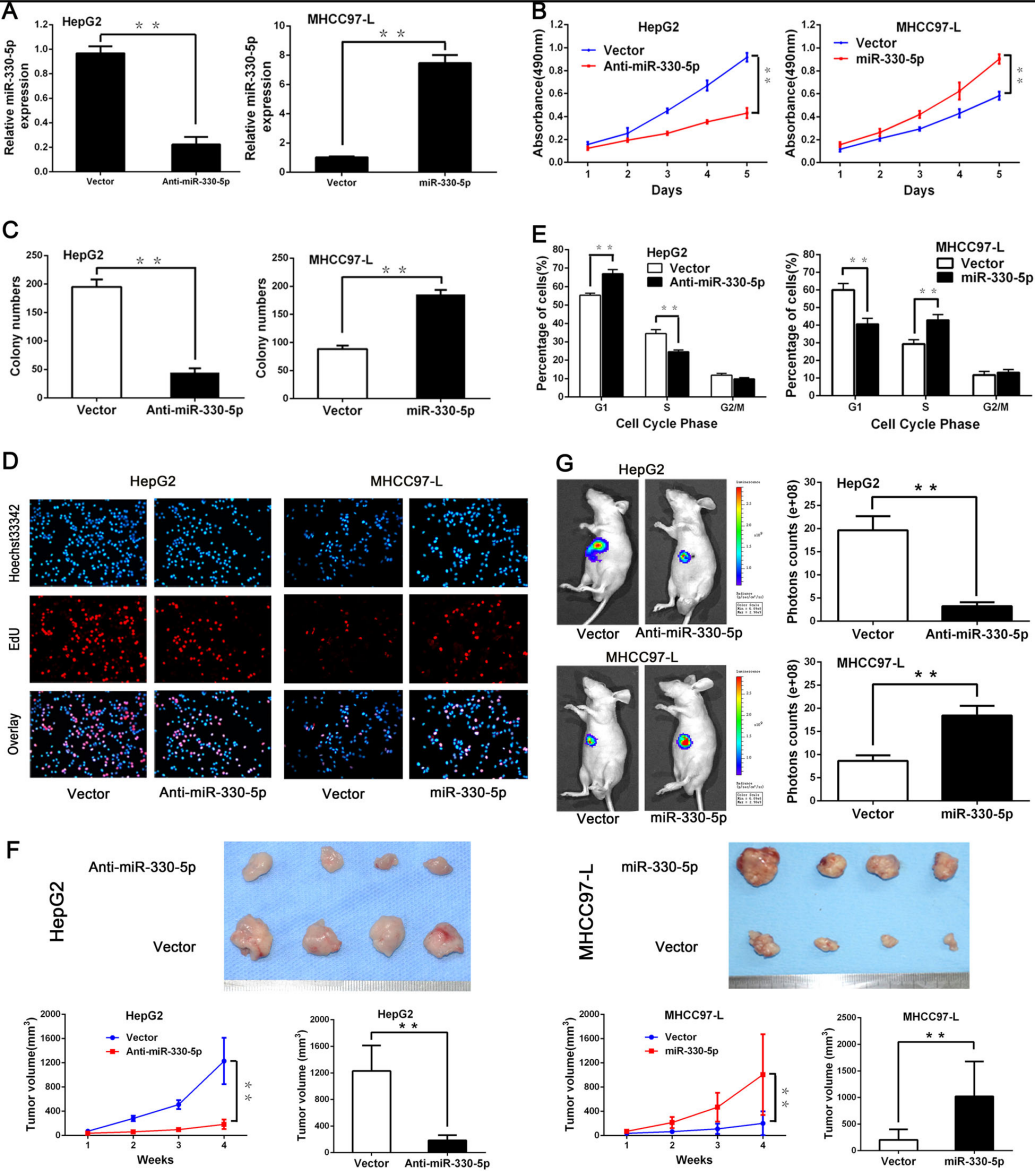
miR-330-5p promoted HCC cells proliferation and growth in vitro and in vivo. **a** The interfered efficiency of miR-330-5p was determined by real-time PCR. **b**-**e** The proliferation of HepG2 or MHCC97-L cells with miR-330-5p interfered was examined by MTT assay (**b**), colony formation assay (**c**), EdU assay(**d**), and cell cycle assay (**e**). **f** The growth of HepG2 or MHCC97-L cells with miR-330-5p interfered was examined by subcutaneous tumor mouse model. The size of liver tumors in these two groups was calculated and compared. **g** *R*epresentative bioluminescence images of the orthotopic HCC mouse model were taken in the eighth week which showed miR-330-5p expression significantly promoted HCC growth in vivo

Because the rapid growth of tumor is the main cause of poor prognosis, we then performed miR-330-5p gain-and-loss-of-function assays to explore the role of miR-330-5p in HCC. We chose HepG2 to establish stable infection of anti-miR-330-5p cells (designed as HepG2^anti-miR-330-5p^) and selected MHCC97-L to construct stable ectopic expression miR-330-5p cells (designed as MHCC97-L^miR-330-5p^) for next functional assays. The interfered efficiency was determined by real-time PCR (Fig. 3a). Results showed the proliferation capacity of HepG2 and MHCC97-L cells was obviously changed by miR-330-5p interference, but invasion potential had no significant change (Supplementary Fig. 1a, b). The methyl thiazolyl tetrazolium (MTT) assay and colony formation assay showed that HepG2^anti-miR-330-5p^ cells had lower proliferation rate and fewer colony numbers than control cells, but MHCC97-L^miR-330-5p^ cells had increased proliferation rate and more colony numbers than control cells (Fig. 3b, c). The 5-Ethynyl-2′-deoxyuridine (EdU) assays also showed the percentage of positive cells was lower in HepG2^anti-miR-330-5p^ compared with control cells, while in MHCC97-L^miR-330-5p^ cells had the opposite effect (Fig. 3d). Then, the cell-cycle analysis further validated that anti-miR-330-5p increased the percentage of HepG2 cells in G1 phase, but decreased the percentage of cells in S phase, while ectopic expression miR-330-5p decreased the percentage of MHCC97-L cells in G1 phase and increased the percentage of cells in S phase (Fig. 3e). These data concluded that miR-330-5p could increase the proliferation capacity of HCC cell in vitro.

We further verified the functional role of miR-330-5p in the mouse model. HepG2^anti-miR-330-5p^, MHCC97-L^miR-330-5p^, and their control cells were injected into the bilateral inguinal region of nude mice respectively, and tumor growth was evaluated. Results showed that subcutaneous tumors generated from HepG2^anti-miR-330-5p^ cells grew slower and smaller than control cells, while tumors from MHCC97-L^miR-330-5p^ cells grew quicker and bigger than control cells (Fig. 3f). Further, we established liver orthotopic HCC mouse model and detected by in vivo imaging system (IVIS) to test the oncogenic function of miR-330-5p in vivo. Consistent with the in vitro and subcutaneous model data, results showed that HepG2^anti-miR-330-5p^ cells generated smaller tumors than control cells, while MHCC97-L^miR-330-5p^ cells generated bigger tumors than control cells (Fig. 3g). Taken these results together, we could draw the conclusion that miR-330-5p promoted HCC growth in vitro and in vivo.

### SPRY2 was a direct target of miR-330-5p in HCC

To explore the potential mechanism of miR-330-5p promotes the growth of HCC, we first searched the validated miR-330-5p associated pathways by miRWalk 2.0 (Supplementary Table 1), and focused in MAPK signaling for the strong effect in promoting proliferation. Then we searched candidate targets for miR-330-5p in miRWalk (TargetScan, miRanda, DIANAmT and PICTAR5 databases) (Supplementary Table 2). Among the hundreds of candidates, we focused the genes which were closely related to MAPK signaling and chose SPRY2 as the potential target of miR-330-5p which was predicted by all the 4 databases. SPRY2 was an important inhibitor for ERK, which was the central member of the canonical MAPK pathway and played a crucial role in promoting cell proliferation, especially in cancers^18^. Therefore, we focus on SPRY2 as the potential target of miR-330-5p for further study.

**Fig. 4:**
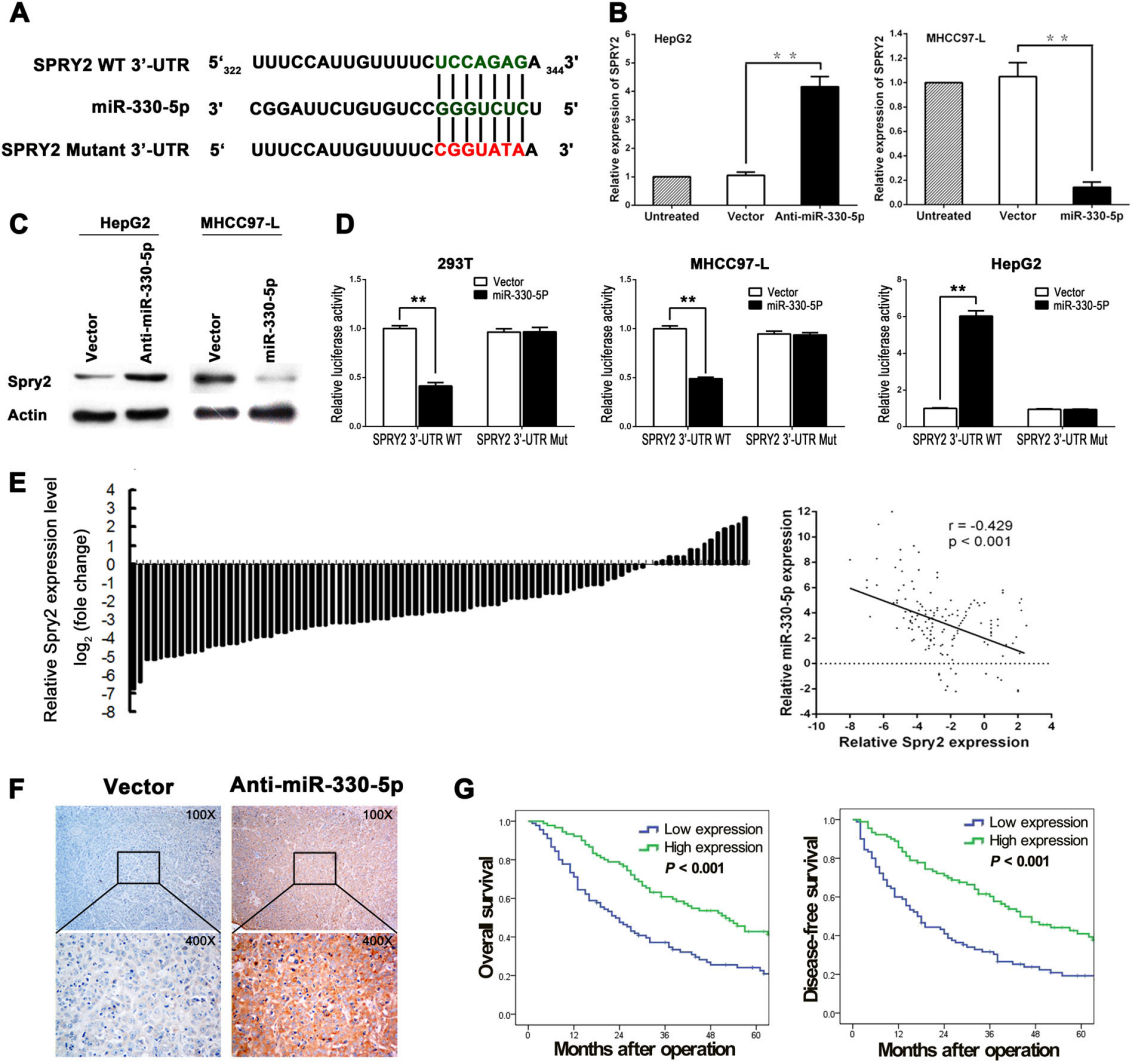
SPRY2 was a direct target of miR-330-5p in HCC. **a** Schematic of putative miR-330-5p binding sequence in the 3′-UTR of SPRY2, and the generation of mutation of SPRY2. **b**, **c** SPRY2 expression was detected in the miR-330-5p interfered HCC cells by real-time PCR (**b**) and western blot (**c**). **d** Relative luciferase activity of SPRY2 WT or MT 3′UTR was detected in HEK-293T and MHCC97-L cells transfected with miR-330-5p, and HepG2 cells transfected with anti-miR-330-5p. **e** SPRY2 mRNA expression in HCC samples was determined by real-time PCR, and showed SPRY2 mRNA expression was negatively correlated with miR-330-5p expression. **f** SPRY2 protein expression in the subcutaneous transplanted tumor from HepG2 cells with miR-330-5p knockdown or control was detected by IHC. **g** Survival curves analysis showed SPRY2 low expression group had a poorer OS and DFS than SPRY2 high expression group

To validate whether miR-330-5p could directly bind to the 3′ UTR of SPRY2, we constructed the luciferase reporter vector of the SPRY2 WT 3′ UTR and the mutant 3′ UTR target sequences (Fig. 4a). The real-time PCR and western blot assays also confirmed that SPRY2 expression was upregulated in HepG2^anti-miR-330-5p^ cells but decreased in MHCC97-L^miR-330-5p^ cells (Fig. 4b, c). As expected, the relative SPRY2-3′UTR WT luciferase activity was significantly decreased in 293 T cells and MHCC97-L cells after transfected with miR-330-5p, but increased in HepG2^Anti-miR-330-5p^ cells which had miR-330-5p knockdown (Fig. 4d). Then we detected the SPRY2 expression in HCC samples, results showed the SPRY2 expression was down-regulation in most of HCCs, which was significantly inversely correlated with the expression of miR-330-5p by Spearman rank correlation (Fig. 4e, *r* = −0.429, *P* < 0.001). We further detected the SPRY2 expression in the tumor of HepG2^anti-miR-330-5p^ cells by immunohistochemical staining, the representative images showed the markedly increased positive staining of SPRY2 compared with control tumor (Fig. 4f). We also analyzed the correlation of SPRY2 expression with HCC patients’ survival, results showed that low SPRY2 expression was associated with poorer OS and DFS (*P* < 0.01, respectively), which was consistent with high miR-330-5p in HCC (Fig. 4g). These results suggested that SPRY2 was the direct downstream target and negatively modulated by miR-330-5p in HCC.

### miR-330-5p suppressed SPRY2 expression to promote HCC growth

**Fig. 5:**
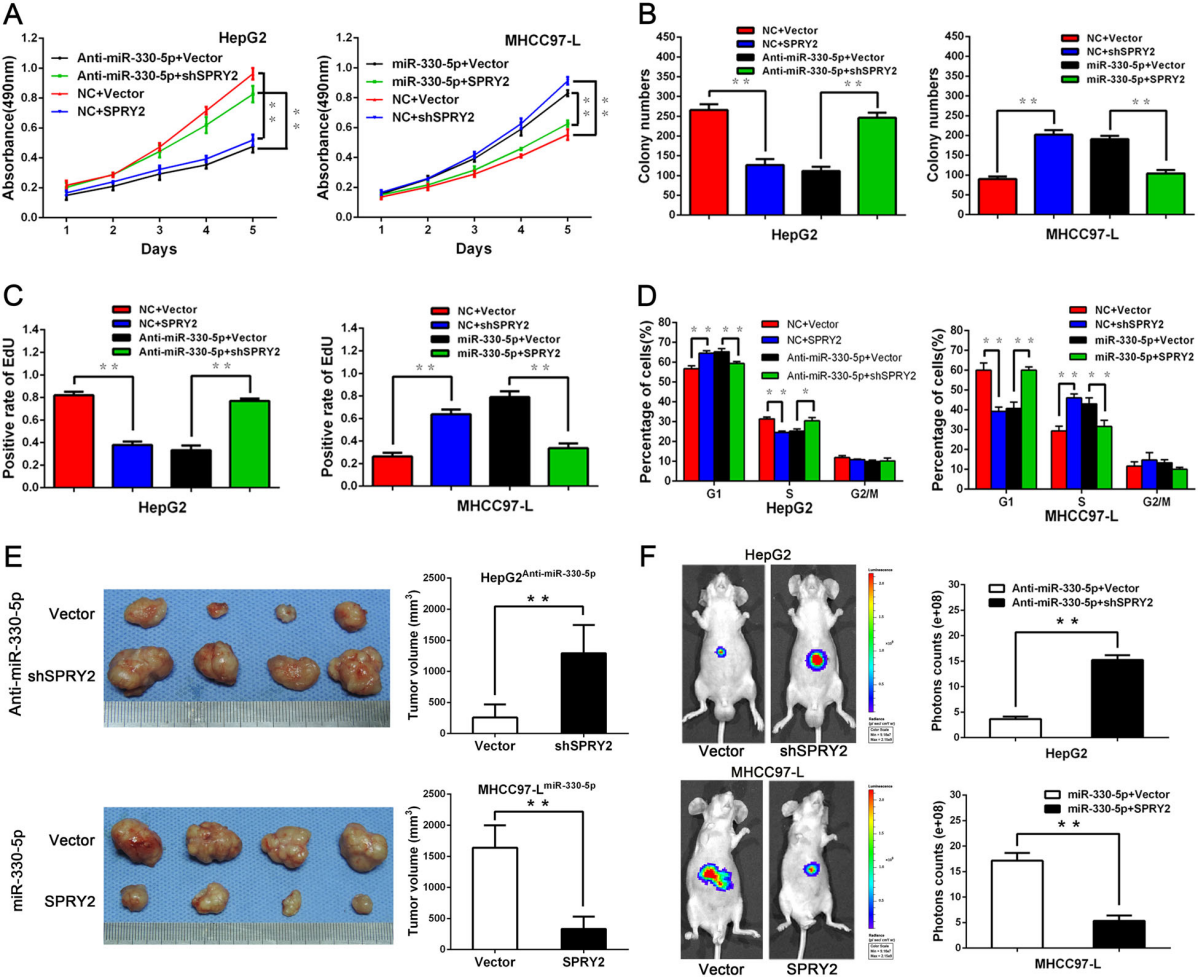
miR-330-5p suppressed SPRY2 expression to promote HCC growth. **a** The proliferation capacity of HepG2^anti-miR-330-5p^ cells with SPRY2 knockdown (HepG2^anti-miR-330-5p+shSPRY2^) and MHCC97-L^miR-330-5p^ cells with SPRY2 ectopic expression (MHCC97-L^miR-330-5p+SPRY2^) and their control cells was assessed by MTT assay (**a**), colony formation assay (**b**), EdU assay (**c**), and cell cycle assay (**d**). **e** The subcutaneous tumor mouse model was used to compare the tumor volume from SPRY2 interfered HepG2^anti-miR-330-5p^ or MHCC97-L^miR-330-5p^ cells. **f** The orthotopic HCC mouse model was used and representative bioluminescence images were taken in the eighth week showed that SPRY2 inhibited tumor growth in vivo

Further assays to validate the functional role of miR-330-5p suppressing SPRY2 in HCC were carried out. We constructed SPRY2 overexpression and knockdown plasmid vector respectively, then transfected the SPRY2 overexpression plasmid into MHCC97-L^miR-330-5p^ cells (MHCC97-L^miR-330-5p+SPRY2^), and SPRY2 knockdown plasmid into HepG2^anti-miR-330-5p^ (HepG2^anti-miR-330-5p+shSPRY2^) cells. The intervention efficiency was assessed by real-time PCR and western blot (Supplementary Fig. 2a, b). The MTT and colony formation assays revealed that SPRY2 overexpression inhibited the proliferation of HepG2 and MHCC97-L^miR-330-5p^ cells, while silence SPRY2 significantly recovered the proliferation ability of HepG2^anti-miR-330-5p^ and MHCC97-L cells (Figs. 5a, b). The EdU and cell cycle assays result further verified these results (Figs. 5c, d). Then, subcutaneous in vivo assays showed that tumors generated from HepG2^anti-miR-330-5p+shSPRY2^ cells were bigger than tumors from HepG2^anti-miR-330-5p^ cells, while tumors generated from MHCC97-L^miR-330-5p+SPRY2^ cells were smaller than tumors from MHCC97-L^miR-330-5p^ cells (Fig. 5e). Next, the tumors from orthotopic HCC model also showed the similar results by IVIS (Fig. 5f).

These gain-and-loss-of-function data from in vitro and in vivo assays showed that miR-330-5p suppressed SPRY2 expression to promote HCC growth.

### miR-330-5p suppressed SPRY2 to promote HCC growth via MAPK/ERK signaling

**Fig. 6:**
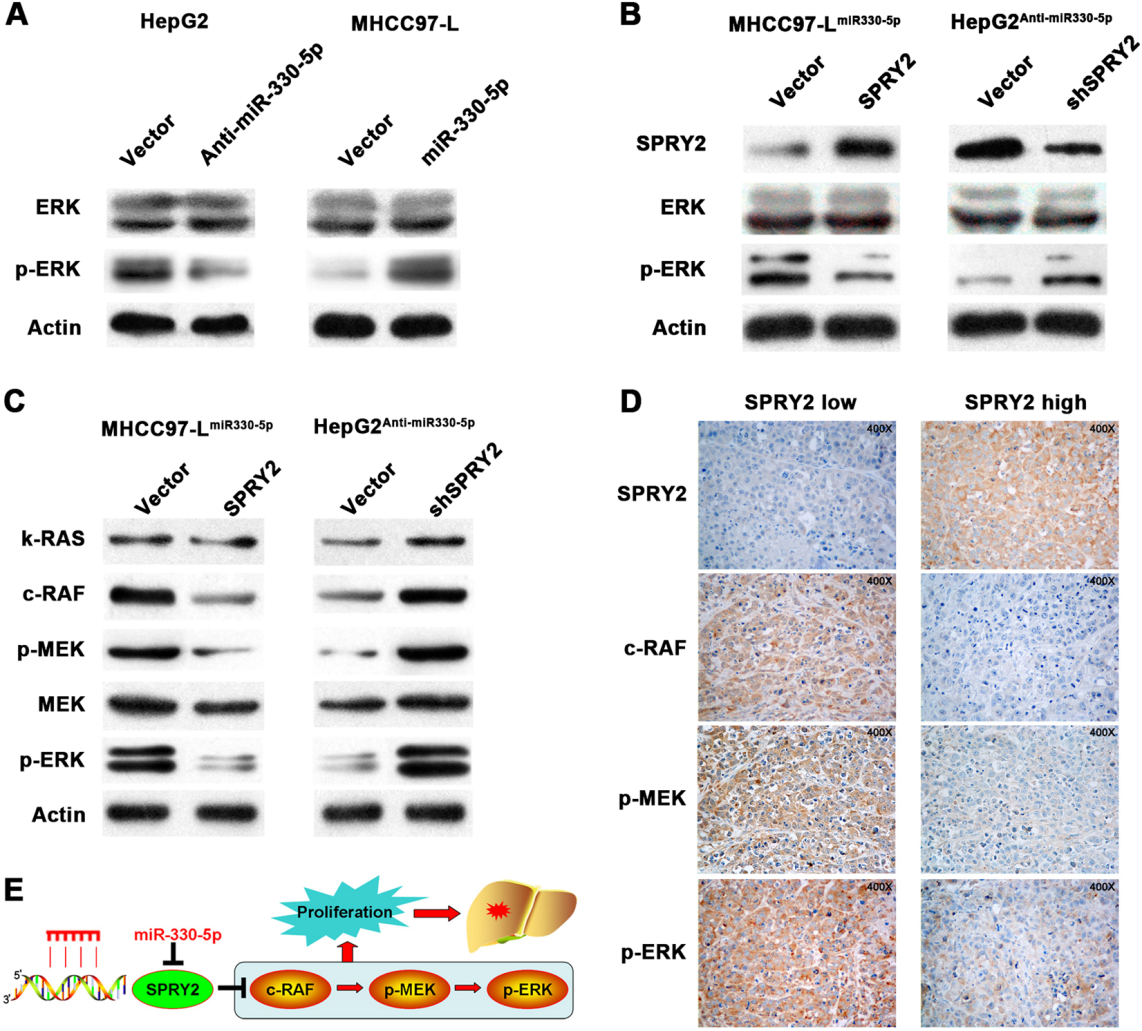
miR-330-5p suppressed SPRY2 to promote HCC growth via MAPK/ERK signaling. **a** ERK and p-ERK expression in miR-330-5p interfered cells were detected by western blot. **b** ERK and p-ERK expression level were detected in SPRY2 interfered HepG2^anti-miR-330-5p^ or MHCC97-L^miR-330-5p^ cells by western blot. **c** The central members of MAPK/ERK signaling (k-RAS, c-RAF, p-MEK, p-ERK and MEK) were detected by western blot. **d** c-RAF, p-MEK and p-ERK protein expressions in subcutaneous transplanted tumor with high or low SPRY2 expression were detected by IHC. **e** The diagram of miR-330-5p targeted SPRY2 and activated MAPK/ERK signaling to promote HCC growth and progression

After we confirmed that miR-330-5p could repress SPRY2 expression to promote the growth of HCC, we next wanted to explore the potential regulatory network. Because SPRY2 was generally considered inhibiting MAPK/ERK signaling, we first detected ERK and p-ERK expression in miR-330-5p interfered cells by western blot. Results showed that miR-330-5p overexpression was associated with high p-ERK expression, while inhibition miR-330-5p expression was associated with low p-ERK expression, but the total ERK expression had no significant change (Fig. 6a). Then we detected the ERK and p-ERK expression in SPRY2-interfered cells and found that SPRY2 expression was negatively correlated with p-ERK expression (Fig. 6b). These data indicated that miR-330-5p might regulate ERK signaling activity via SPRY2. We further studied the role of SPRY2 on the central members of ERK signaling. Results showed that SPRY2 overexpression inhibited the expressions of c-RAF, p-MEK and p-ERK in MHCC97-L^miR-330-5p^ cells, while SPRY2 silence recovered the expression of c-RAF, p-MEK and p-ERK in HepG2^anti-miR-330-5p^ cells, and had no obvious effect on K-RAS and MEK (Fig. 6c). These data further indicated that SPRY2 modulated the activity of ERK signaling via c-RAF and then induced the phosphorylation of MEK and ERK. Besides these, we detected the expression of SPRY2, c-RAF, p-MEK and p-ERK in xenografts from SPRY2-interfered HCC cells by IHC. Results also showed in SPRY2 low expression HCC tissues always had high c-RAF, p-MEK and p-ERK expression, while in SPRY2 low expression HCC tissues had the low c-RAF, p-MEK, and p-ERK expression (Fig. 6d). Taken together, we concluded that miR-330-5p suppressed SPRY2 to promote proliferation via activating c-RAF-MEK-ERK signaling in HCC (Fig. 6e).

## Discussion

Uncontrollable cell proliferation is one of the fundamental hallmarks of cancer progression^3^. Increasing studies have demonstrated that miRNAs played a crucial role in cancer cell proliferation^5^. Consistent with these, our previous miRNAs profiling has found that miR-140-5p, miR-331-3p, miR-188-5p and etc. had a great effect on HCC proliferation, growth, progression and correlated with poor prognosis of HCC patients^12–14^. In this study, we first found and confirmed that miR-330-5p significantly promoted HCC proliferation, growth, and progression in vitro and in vivo, which was not reported before. And these findings further verified the important value of miRNAs in HCC progression.

miR-330-5p and miR-330-3p were the mature sequences generated from 5′ or 3′ terminal of miR-330 pre-miRNA. miR-330 aberrant expression was closely related to cancer development and progression, such as glioblastoma, colorectal cancer, prostate cancer, as well as HCC^15–17,19^. However, the exact role of miR-330 in cancer was still controversial, and so was miR-330-5p. Some studies found miR-330-5p functioned as a tumor suppressor in pancreatic cancer, colorectal cancer and glioblastoma, while our study indicated the oncogenic role in HCC which was consistent with the previous study of miR-330 in HCC^15,20–22^. Therefore, our findings suggested that both miR-330 and miR-330-5p were functioned as oncogenic miRNAs in HCC, which expanded our understanding of miR-330 in cancer.

More interestingly, we found miR-330-5p expression levels were similar in SHCC and SLHCC which had obviously different tumor sizes, but miR-330-5p expression level was elevated in NHCC with the increase of size. These data seemed to be a contradiction at first view, but we thought that explained why SHCC and SLHCC were different to NHCC. The potential explanation was that SHCC and SLHCC had relatively low metastasis potential and the tumor size of them might just reflect the growth time but not invasion ability^23–25^. However, NHCC had an obvious higher aggressive capacity and tumor size was a marker of aggressive potential, thus miR-330-5p expression level in NHCC was elevated with increased tumor size. These findings were also consistent with our previous studies that size of the solitary tumor was not a determining factor for the staging of HCC, which was also supported by some other studies^7,26^.

In this study, we have explored the molecular mechanism of miR-330-5p in promoting HCC progression. We found and verified SPRY2 was the direct target, SPRY2 was generally considered as an inhibitor of MAPK/ERK signaling and played an important role in embryonic development and stemness maintenance^27–29^. Recent years, researchers had further recognized that SPRY2 had a great capacity in regulating cell proliferation, survival, migration, and angiogenesis, thus became a hotspot in cancer progression^30,31^. Existing studies showed that SPRY2 inactivation promoted various cancer development and progression, such as melanoma, lymphoma, gastric cancer^32–34^. Since Fong first reported that SPRY2 was down-regulated and inhibited proliferation in HCC in 2006, there were many studies confirmed that SPRY2 inactivation was powerful for hepatocarcinogenesis, but no study involved in HCC progression^35,36^. Based on this, our study had not only validated the previous studies but also further indicated that SPRY2 inactivation by miR-330-5p promoted HCC progression and predicted poor prognosis. Therefore, our findings provided novel clues of SPRY2 in HCC progression and might develop a novel biomarker for HCC surveillance and treatment.

The potential molecular mechanism of SPRY2 inactivation was also studied in this study. Numerous studies had confirmed that SPRY2 played its biologic role mainly through inhibiting the canonical MAPK/ERK signaling, which was composed and consecutively activated by EGFR, RAS, RAF, MEK, and ERK^37^. In this study, we found SPRY2 regulated the expression of c-RAF, p-MEK, and p-ERK in HCC, but had no effect on k-RAS, MEK and ERK. Therefore, SPRY2 might exert its ERK inhibitor role via suppressing c-RAF in HCC, which were consistent with prior studies in leukemia^38,39^. RAF-MAPK/ERK was an important signaling for targeting drugs such as regorafenib and sorafenib, thus our findings potentially provided a new clue for developing anovel therapeutic approach for HCC^40^.

In summary, our study has well defined the oncogenic role and promising prognostic factor of miR-330-5p in HCC. We have verified miR-330-5p directly targeted SPRY2 to activate MAPK/ERK signaling to promote HCC progression, which provided an important evidence that targeting miR-330-5p would be the promising therapeutic approach for HCC treatment.

## Materials and methods

### HCC patients, samples, and data

One hundred and eighty randomly group selected patients (60 SHCC, 60 SLHCC, and 60 NHCC) who underwent HR for HCC and had detailed clinicopathological and prognostic data at Department of Surgery, Xiangya Hospital of Central South University (Changsha, China) from January 2007 to December 2010 were included in this study. Only patients with initially diagnosed with HCC were enrolled, and none of them accepted chemotherapy, transcatheter arterial chemoembolization or radiotherapy before the operation. Diagnosis of HCC was confirmed by two independent histopathologists blind to the information of patients. The surgical indication was HCC patient with enough residual liver volume, and lack of distant metastasis, decompensated cirrhosis, and organic dysfunction. The snap-frozen and paraffin embedded HCCs and ANLTs were collected and reserved. Tumor recurrence and metastasis were detected by serum AFP, ultrasonography and chest x-ray every 3 months in the first 2 years, and twice a year in the next 5 years. Contrast-enhanced Computed Tomography scan or Magnetic Resonance Imaging was performed if relapse or metastasis was suspected clinically. The OS defined as the time from HR to death for HCC or to the date of the last follow-up; DFS defined as the time from the date of HR to the date when relapse or metastasis was detected. The complete clinical, pathological and prognosis data of these patients were collected and stored in our database by a researcher fellow. The present prognostic study strictly follows the consensus of the Reporting Recommendations for Tumor Marker Prognostic Studies (REMARK) guideline^41^. All the human materials and matching information (all anonymous) were obtained with a written informed consent form, and approved by the ethics committee of Xiangya Hospital of Central South University. All the experiments and procedures were in accordance with the Helsinki Declaration.

### In vivo assay

First, luciferase-labeled HepG2 and MHCC97-L cell lines are built via transfecting by recombinant plasmid pSin-hyg-GFP/luc2 (Berthold, Bad Wildbad, Germany). Then, the HCC subcutaneous and orthotopic transplantation model in nude mice were constructed as described before^12^. Briefly, 5 × 10^6^ treated cells were subcutaneously injected into the left buttock regions of 4 nude mouse (randomly selected, 3–4 weeks of age, male, BALB/c), and the untreated cells were injected into the right buttock of the same mouse as a control. After 4 weeks of implantation, the mice were sacrificed, and the subcutaneous tumor size was calculated and recorded used vernier caliper as follows: tumor volume (mm^3^) = (L × W^2^)/2, where L = long axis and W = short axis^42^. All the measurements were repeated three times. Then the subcutaneous tumor was cut into pieces of the size as 1 mm^3^ and implanted into the liver of each group (also random selected, 4 in each group). The activity of luciferase was detected by IVIS Lumina II (Xenogen, Hopkinton, MA) every week according to the protocol of IVIS. After 8 weeks of implantation, the mice were sacrificed, and the size of tumors was calculated and compared as mentioned above. Serial sections were subjected to IHC staining. The animal management and experimental procedures were carried out in accordance with the Guidelines of National Institutes of Health for the Care and Use of Laboratory Animals, and approved by the Medical Experimental Animal Care Commission of Central South University.

### Statistical analysis

Statistical analyses were performed using SPSS 18.0 for Windows (SPSS Inc., Chicago, IL) and GraphPad Prism6 (GraphPad Software Inc., La Jolla, CA). Data were expressed as the mean ± standard error of the mean from at least three independent experiments. Quantitative data between groups were compared using the Student *t* test or analysis of variance. Dichotomous variables were analyzed by the *χ*^2^ test or Fisher exact test. Correlations between different protein expressions level were analyzed using Spearman’s rank analysis. Overall survival and disease-free survival curves were obtained by the Kaplan-Meier method, and differences were compared by the log-rank test. Univariate analysis and multivariate analysis were analyzed with Cox proportional hazard regression model to verify the independent risk factors. A *P*-value < 0.05 was considered statistically significant.

## Electronic supplementary material

Supplementary Materials and Methods

Supplementary Figure 1

Supplementary Figure 2

Supplementary Figure 3

Supplementary Table S1

Supplementary Table S2

Ethics approval

Checklist
