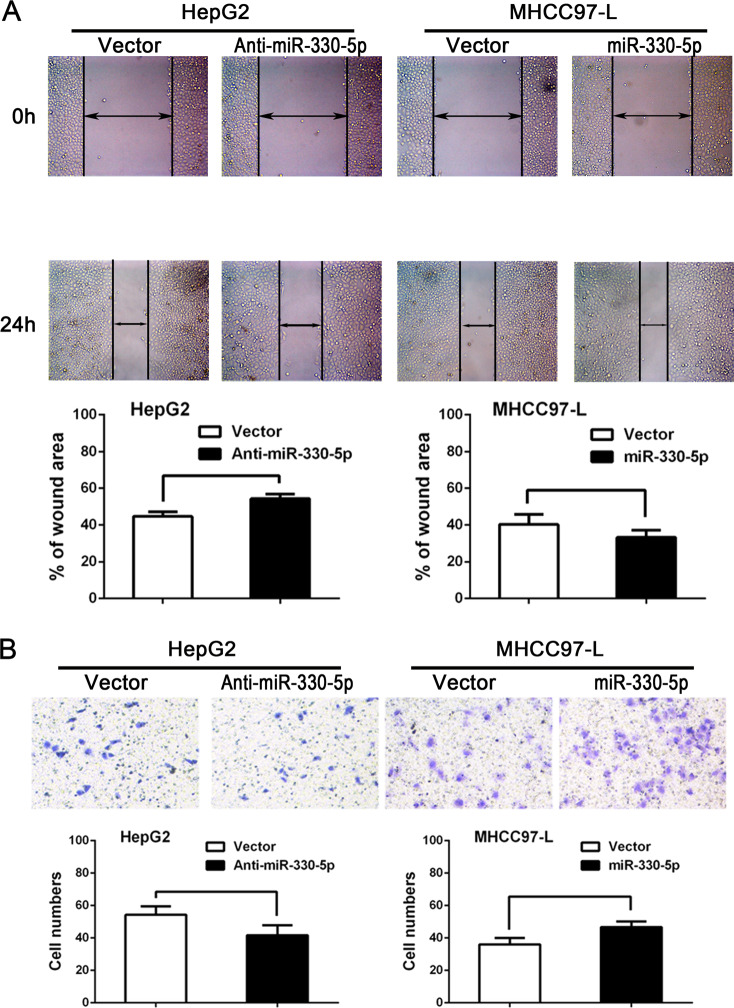# Correction to: miR-330-5p targets SPRY2 to promote hepatocellular carcinoma progression via MAPK/ERK signaling

**DOI:** 10.1038/s41389-021-00340-z

**Published:** 2021-07-13

**Authors:** Shuai Xiao, Mengyuan Yang, Hao Yang, Ruimin Chang, Feng Fang, Lianyue Yang

**Affiliations:** 1 Liver Cancer Laboratory, Xiangya Hospital, Central South University, Changsha, Hunan China; 2 Department of Obstetrics and Gynecology, The Second Xiangya Hospital of Central South University, Changsha, Hunan China

**Keywords:** Liver cancer, Metastasis

Correction to: *Oncogenesis* 10.1038/s41389-018-0097-8, published online 21 November 2018

Following the publication of this article, it was noted that there was a duplication of wound-healing images at 0 h for MHCC97-L cells in Supplementary Fig. 1a. The corrected image is provided below. The authors confirm this error has no effect on the scientific conclusions of this article.