# Supplementary material for: miR-330-5p targets SPRY2 to promote hepatocellular carcinoma progression via MAPK/ERK signaling
Source: Oncogenesis. 2018 Nov 21;7(11):90. doi: 10.1038/s41389-018-0097-8 (PMC6249243; doi:10.1038/s41389-018-0097-8)
Supplement: Supplementary file 1 — Supplementary Materials and Methods [file 41389_2018_97_MOESM1_ESM.doc]

**Supplementary Materials and Methods**

**Cell lines and cell culture**

HepG2 cell line was purchased from the American Type Culture Collection (ATCC, Manassas, VA). Liver cancer cell lines MHCC97-L and HCCLM3 were kindly presented by the Liver Cancer Institute of Fudan University, Shanghai, China. Liver cell line L02 was purchased from the Cell Bank of Typical Culture Preservation Committee of Chinese Academy of Science, Shanghai, China. Cell culture was according to the manufacturer's protocol and all the cell lines growed at 37℃ with 5% CO_2_.

**RNA extraction and Real-Time PCR**

TRIzol® Reagent (Life Technologies, Carlsbad, CA) was used to isolate and extract total RNA from frozen patient samples and cells according to the manufacturer’s protocol. The miRNA expression level was analyzed using TaqMan® microRNA reverse transcription kit and TaqMan® universal PCR master mix (Ambion, Austin, TX) as previously described (1). To quantify mRNA expression, the RNA was reversely-transcribed by the universal cDNA synthesis kit (Toyobo, tokyo, JP) to obtain cDNA, then subjected to real-time PCR using the SYBR green PCR kit (Roche Life Sciences, Indianapolis, IN) and the reagents was detected by PRISM 7300 sequence detection system (Applied Biosystems, Foster City，CA) according to the manufacturer’s instructions(2). RNU6B (U6) or GAPDH was used as an internal control for miRNA or mRNA detection. The experiments were done in triplicate. The miR-330-5p RT and real-time PCR primer sequences were: miR-330-5p-RT: 5'-CTCAACTGGTGTCGTGGAGTCGGCAATTCAGTTGAGGCCTAAGA-3'; miR-330-5p-forward: 5’- TCTCTGGGCCTGTGTC-3’, miR-330-5p- reverse: 5’- CCAGTTTTTTTTTTTTTTTGCCTAAG-3’. The SPRY2 primer sequences used in real-time PCR were: forward 5'-TCGCAGCCTTTGCTGCAGTGT-3' and reverse 5'-TTAGGCTGCACTCGGATTATT-3'.

**Vector construction**

The DNA fragment of miR-330-5p was amplified from genomic DNA and inserted into the BamHI/HindIII site of lentivirus expression vector pGCsil-009 (GenePharma, Shanghai, China). MiR-330-5p mimic or miR-330-5p inhibitor was used for miR-330-5p ectopic expression or suppression, respectively. The SPRY2 expression vector was constructed by inserting its CDS sequence into the pEX-2 vector (GeneChem, Shanghai, China). The SPRY2-wild type expression vector was constructed by inserting overall sequence of SPRY2 into pEX-2 vector (Gene Pharma). The SPRY2 siRNAs were purchased from GenePharma Company (Shanghai, China). The wild type and mutant 3’-UTR sequences of SPRY2 was amplified from human liver genomic DNA and then cloned into the downstream region of firefly luciferase cassette in pGL3 vector. The anti-miR-330-5p sequence was 5’-GCCTAAGACACAGGCCCAGAGA-3’, the SPRY2 siRNA sequences for lentivirus construct in this study were as follows, siRNA-1: 5’-AAGTGCAAGTGTAAGGAGTGC-3’, siRNA-2: 5’- AACAGAGACTGTTAGGACCGT-3’, siRNA-3: 5’-AACACCAATGAGTACACAGAG-3'. According to the intervention efficiency analysis (Supplementary Figure 1A, B), we chose siRNA-3 for follow-up experiments.

**Western blot**

Total proteins were extracted with RIPA lysis buffer, 50–100μg of protein extracts were separated by SDS-PAGE and then transferred to the PVDF membrane (Roche Life Sciences, Indianapolis, IN). The membrane were blocked with 5% skimmed milk and incubated with the appropriate primary antibody: mouse anti-human SPRY2 (1:1000, Santa Cruz, Dallas, TX); rat anti-human k-RAS (1:3000, Abcam); mouse anti-human c-RAF (1:2000, Abcam); mouse anti-human p-MEK (1:2000, Santa Cruz); mouse anti-human p-ERK (1:1000, Santa Cruz); mouse anti-human MEK (1:3000, Santa Cruz); mouse anti-human ERK (1:1500, Santa Cruz); mouse anti-human actin (1:3000, Sigma Aldrich). The antigen-antibody complex on the membrane was detected with enhanced chemiluminescence regents (Thermo Scientific, Waltham, MA).

**Immunohistochemistry (IHC)**

Immunohistochemical staining for tissue was performed using the polymer horseradish peroxidase (HRP) detection system (Zhongshan Goldenbridge Biotechnology, Shanghai, China) on formalin-fixed, paraffin-embedded tissue sections that had been cut to 4 µm thickness according to the instructions as described previously (3). Appropriate positive and negative controls were included for each run of IHC. The antibodies were SPRY2 (1:200, Santa Cruz), MEK (1:200, Santa Cruz), ERK (1:400, Santa Cruz), p-MEK (1:400, Santa Cruz) and p-ERK (1:300, Santa Cruz). IHC results were photographed using an optical microscope (Olympus, Tokyo, Japan) and assessed by two independently pathologists in our institution according to previous report (4, 5).

**MTT assay, EdU assay, cell cycle assay and colony formation assay**

Cells were seeded into each well of 96-well plates at a density of 5 × 10^3^ cells/well. Six wells of each group were detected every day. 100 μl fresh medium containing MTT (Sigma, St Louis, MO) 0.5 mg/ml was put into each well and incubated at 37°C for 4 hrs, then the medium was replaced by 100 μl of DMSO and shaken at room temperature for 10 min. The absorbance was measured at 490 nm. For EdU assay, cells were seeded into each well of 96-well plates at a density of 1 × 10^5^ cells/well, then cell proliferation was assessed by Cell-Light EdU DNA cell proliferation kit (RiboBio, Guangzhou, China), according to the manufacturer’s instructions. For cell cycle analysis, 1×10^6^ cells were harvested and washed in PBS, then fixed in 75% alcohol for 60 min at 4°C. Cell cycle analysis was conducted by flow cytometry using a propidium iodide (PI) cell cycle detection kit (Beyotime Institute of Biotechnology, Beijing, China). For colony formation assays, cells were seeded into 35 mm dishes (Corning, NY) at a density of 5 × 10^2^ cells/dish and cultured for 2 weeks at 37°C. The numbers of colonies per dish were counted after staining with crystal violet. All studies were conducted with 3 replicates.

Wound-healing assay and Trans-well invasion assay

These assays were conducted as described (1-3). Cells cultured in six-well plates with medium containing DMEM with 10% FBS, when cells grew to 90% confluence they were pre-incubated with Mitomycin-C (10 μg/ml) for 1h at 37 °C to suppress cell proliferation, next rinsed with phosphate-buffered saline (PBS), and then starved for 24 hours in serum-free medium. A sterile 10 μL pipette tip was used to create three separate, parallel wounds, and migration of the cells across the wound line was assessed after 24 h. For trans-well invasion assay, cells were pre-incubated with Mitomycin-C (10 μg/ml) for 1h at 37 °C, then 1 × 10^5^ cells in serum-free medium containing 0.1% bovine serum albumin were placed into the upper chamber of the insert with matrigel (BD Biosciences, MA). After 12 h of incubation at 37°C, the cells remained in the upper chamber or on the upper membrane were removed. The cells adhering to the lower membrane of the inserts was counted after staining with a 0.1% crystal violet and 20% methanol. The numbers of cells was counted under an inverted microscope (Nikon, Tokyo, Japan).

**Dual luciferase reporter assay**

Luciferase activity was assessed according to the Dual-Luciferase Reporter Assay protocol (Promega, Madison, WI). HEK-293T cells infected with miR-330-5p lentivirus or control virus were seeded in 96-well plates with 70% confluence. 12 hours later, the cells were cotransfected with 50 ng pGL3-UTR and 10 ng pRLTK by using Lipofectamine LTX. 24 hr after transfection, firefly and Renilla luciferase signals were measured using the Dual-Luciferase Reporter Assay System (Promega, Madison, WI). The Renilla luciferase activities were used as an internal control for transfection efficiency.

**References:**

1. Yang H, Fang F, Chang R, Yang L. MicroRNA-140-5p suppresses tumor growth and metastasis by targeting transforming growth factor beta receptor 1 and fibroblast growth factor 9 in hepatocellular carcinoma. HEPATOLOGY 2013;58: 205-17.

2. Chang RM, Yang H, Fang F, Xu JF, Yang LY. MicroRNA-331-3p promotes proliferation and metastasis of hepatocellular carcinoma by targeting PH domain and leucine-rich repeat protein phosphatase. HEPATOLOGY 2014;60: 1251-63.

3. Yang H, Zheng W, Shuai X*, et al*. MicroRNA-424 inhibits Akt3/E2F3 axis and tumor growth in hepatocellular carcinoma. ONCOTARGET 2015;6: 27736-50.

4. Wang C, Delogu S, Ho C*, et al*. Inactivation of Spry2 accelerates AKT-driven hepatocarcinogenesis via activation of MAPK and PKM2 pathways. J HEPATOL 2012;57: 577-83.

5. Lee SA, Ladu S, Evert M*, et al.* Synergistic role of sprouty2 inactivation and c-Met up-regulation in mouse and human hepatocarcinogenesis. HEPATOLOGY 2010;52: 506-17.

**Supplementary Figures Legends**

**Supplementary Figure 1. The role of miR-330-5p in invasiveness capacity of HCC cells.** (A) Wound-healing assays showed miR-330-5p knockdown or ectopic expression had no significant change in HCC cell migration capacity. (B) Trans-well invasion assays showed miR-330-5p knockdown or ectopic expression had no significant change in HCC cell invasion capacity.

**Supplementary Figure 2. The interfered efficiency of SPRY2 in miR-330-5p interfered HCC cells.** (A) The SPRY2 mRNA expression level was detected by real-time PCR after SPRY2 silence or ectopic expression. (B) The SPRY2 protein expression level was detected by western blot after SPRY2 interfered. **: *P* value < 0.01.

**Supplementary Figure 3. The proliferation capacity of HepG2 cells and MHCC97-L cells.** (A-C) The *in vitro* proliferation assays showed HepG2 cells had stronger proliferative capacity than MHCC97-L cells detected by MTT assay (A), cell cycle assay (B) and colony formation assay (C). (D) The subcutaneous tumor mouse model showed HepG2 cells generated larger tumor than MHCC97-L cells. (E) *The IVIS results showed* tumor of orthotopic HCC mouse model generated from HepG2 cells had more photons counts than MHCC97-L cells. *: *P* value < 0.05. **: *P* value < 0.01.
