# Supplementary figures and images for: miR-330-5p targets SPRY2 to promote hepatocellular carcinoma progression via MAPK/ERK signaling
Source: Oncogenesis. 2018 Nov 21;7(11):90. doi: 10.1038/s41389-018-0097-8 (PMC6249243; doi:10.1038/s41389-018-0097-8)

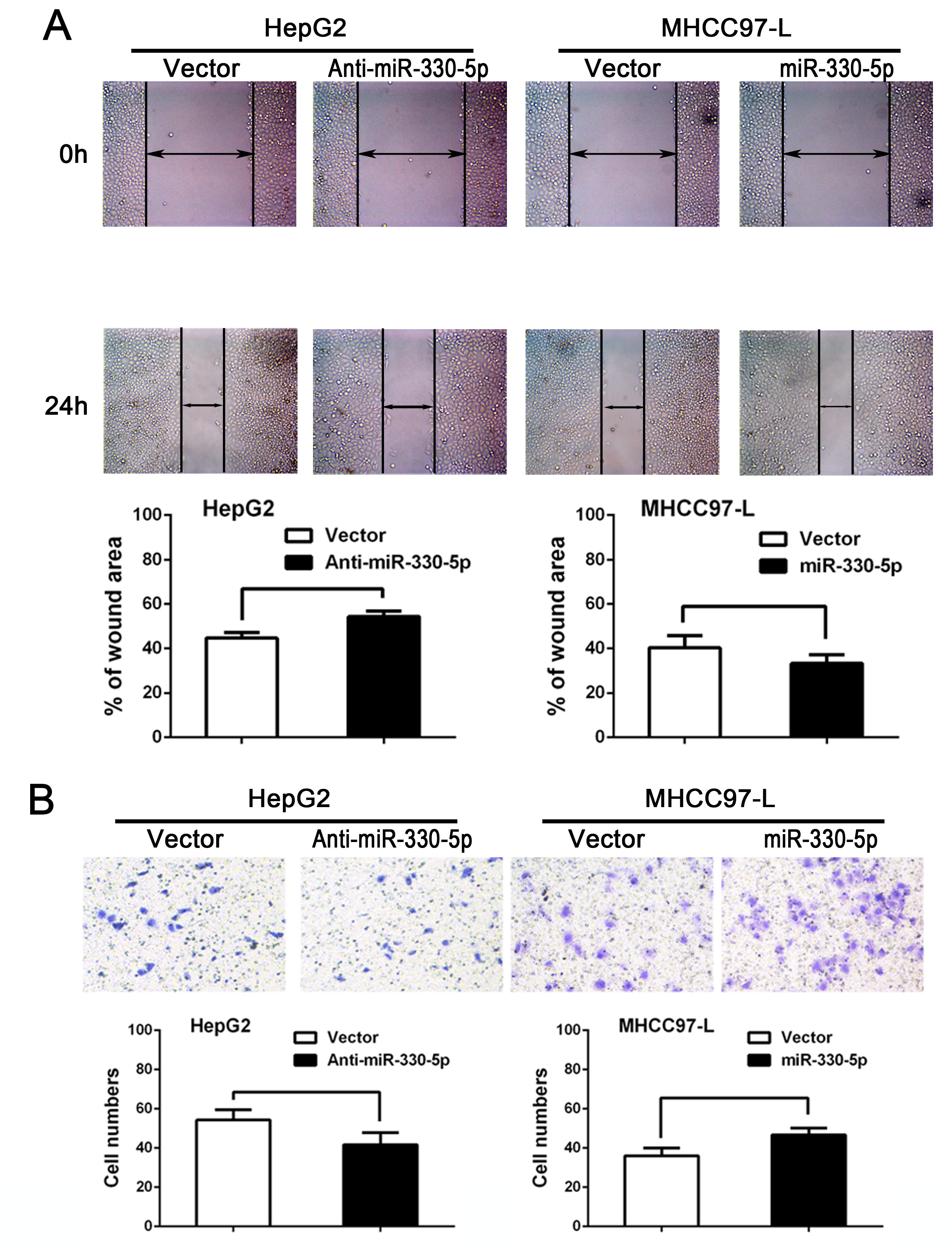

Supplement: Supplementary file 2 — Supplementary Figure 1 [file 41389_2018_97_MOESM2_ESM.jpg]

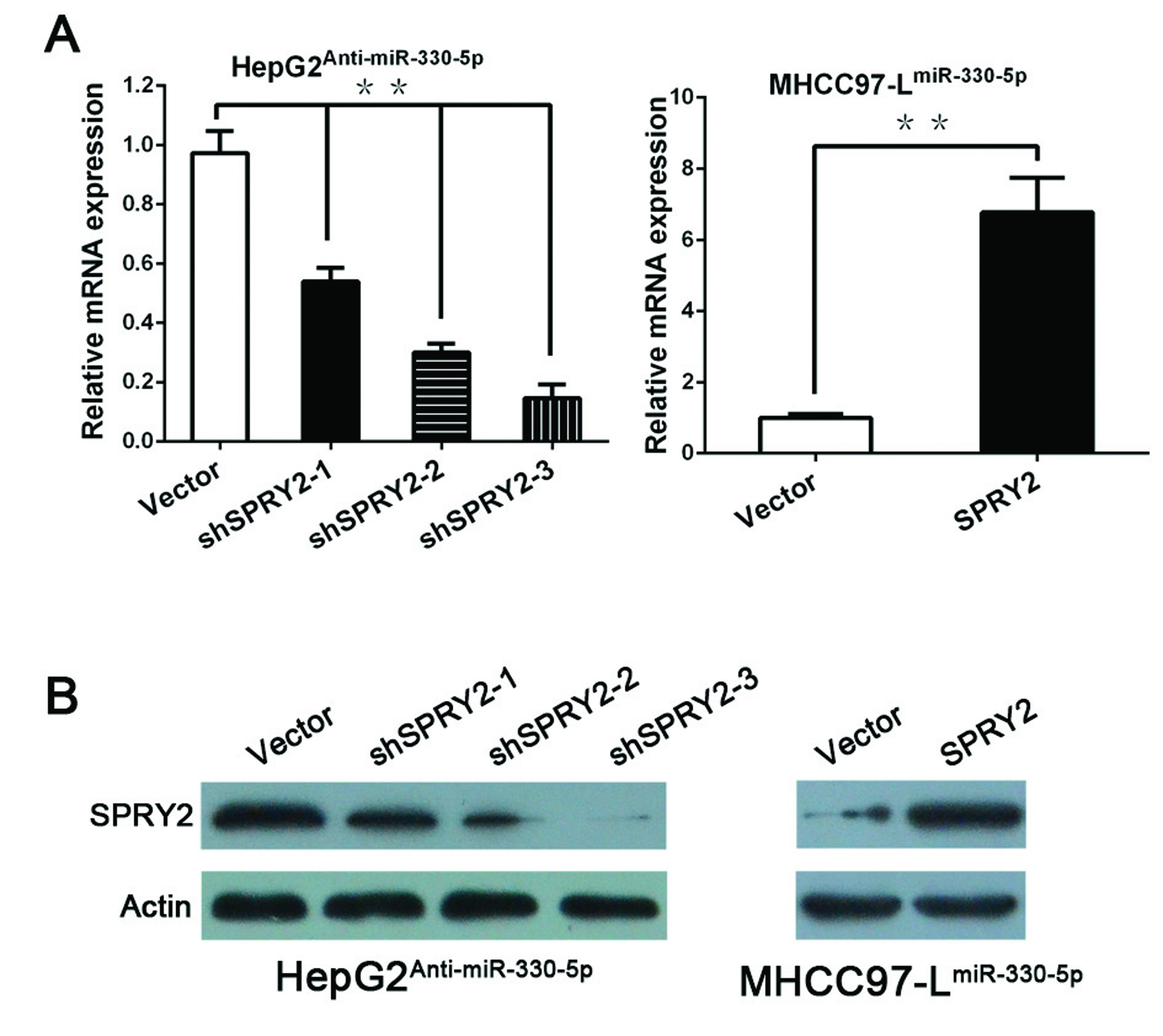

Supplement: Supplementary file 3 — Supplementary Figure 2 [file 41389_2018_97_MOESM3_ESM.jpg]

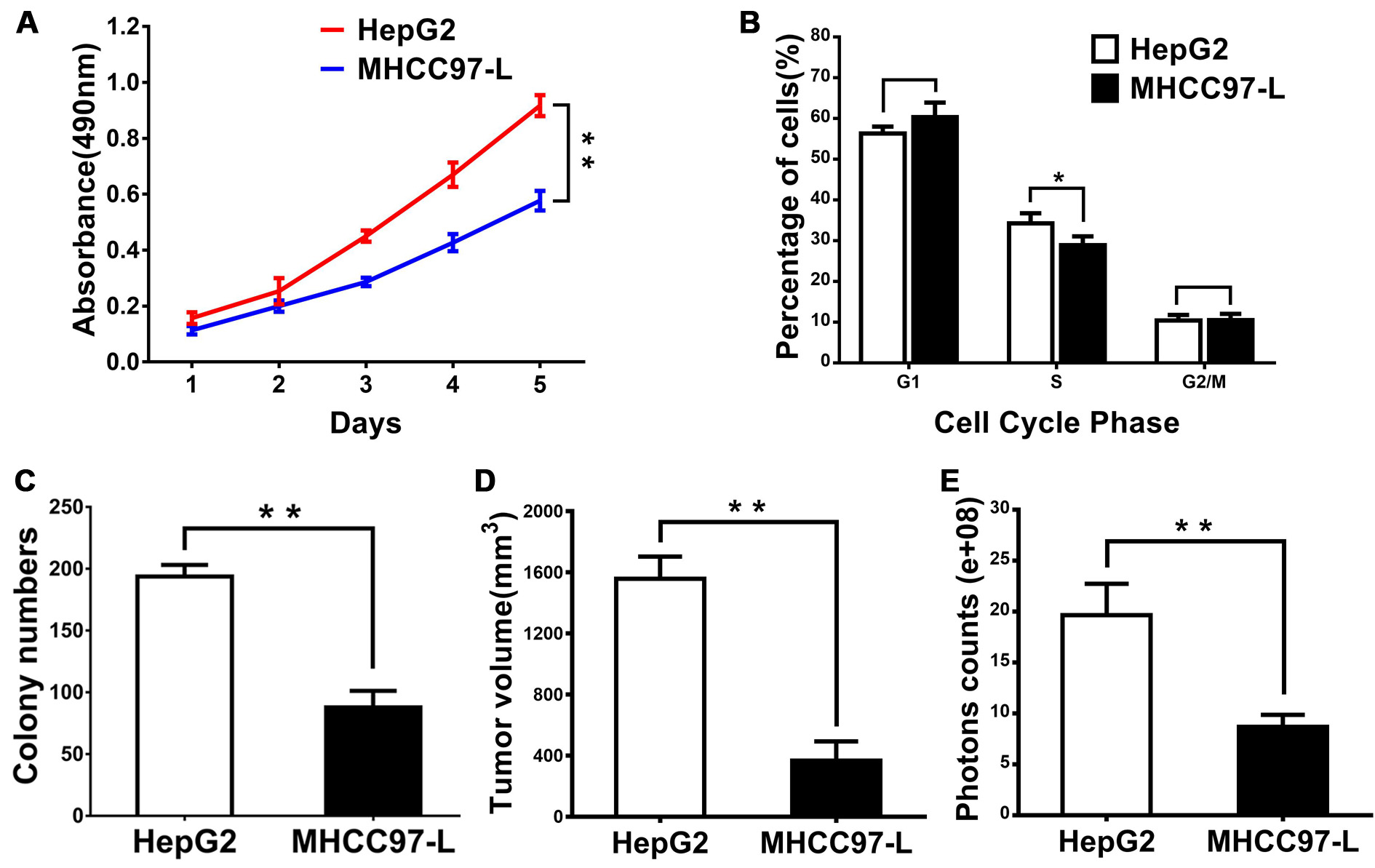

Supplement: Supplementary file 4 — Supplementary Figure 3 [file 41389_2018_97_MOESM4_ESM.jpg]

Ethics approval for human:


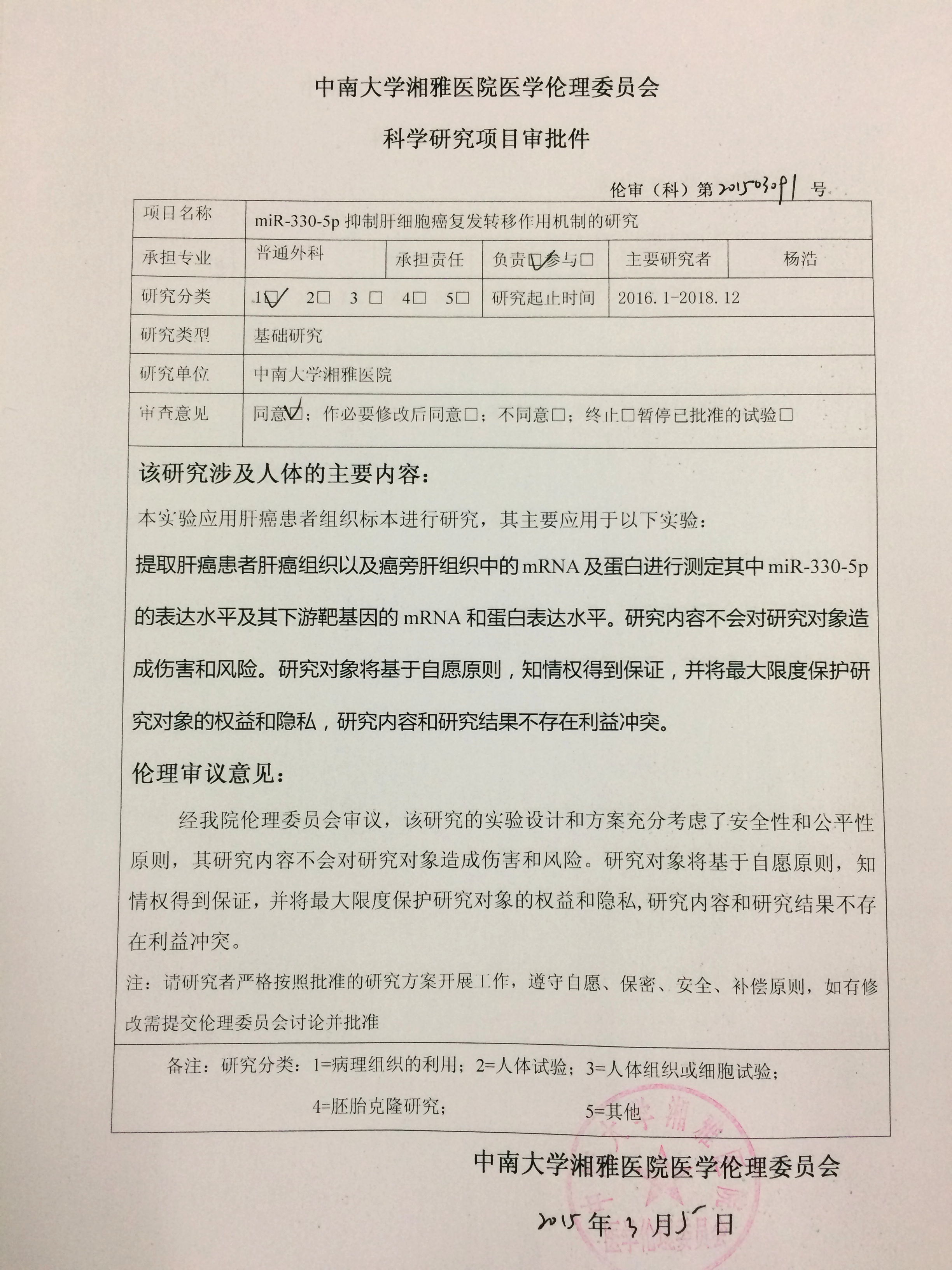


Ethics approval for animal:


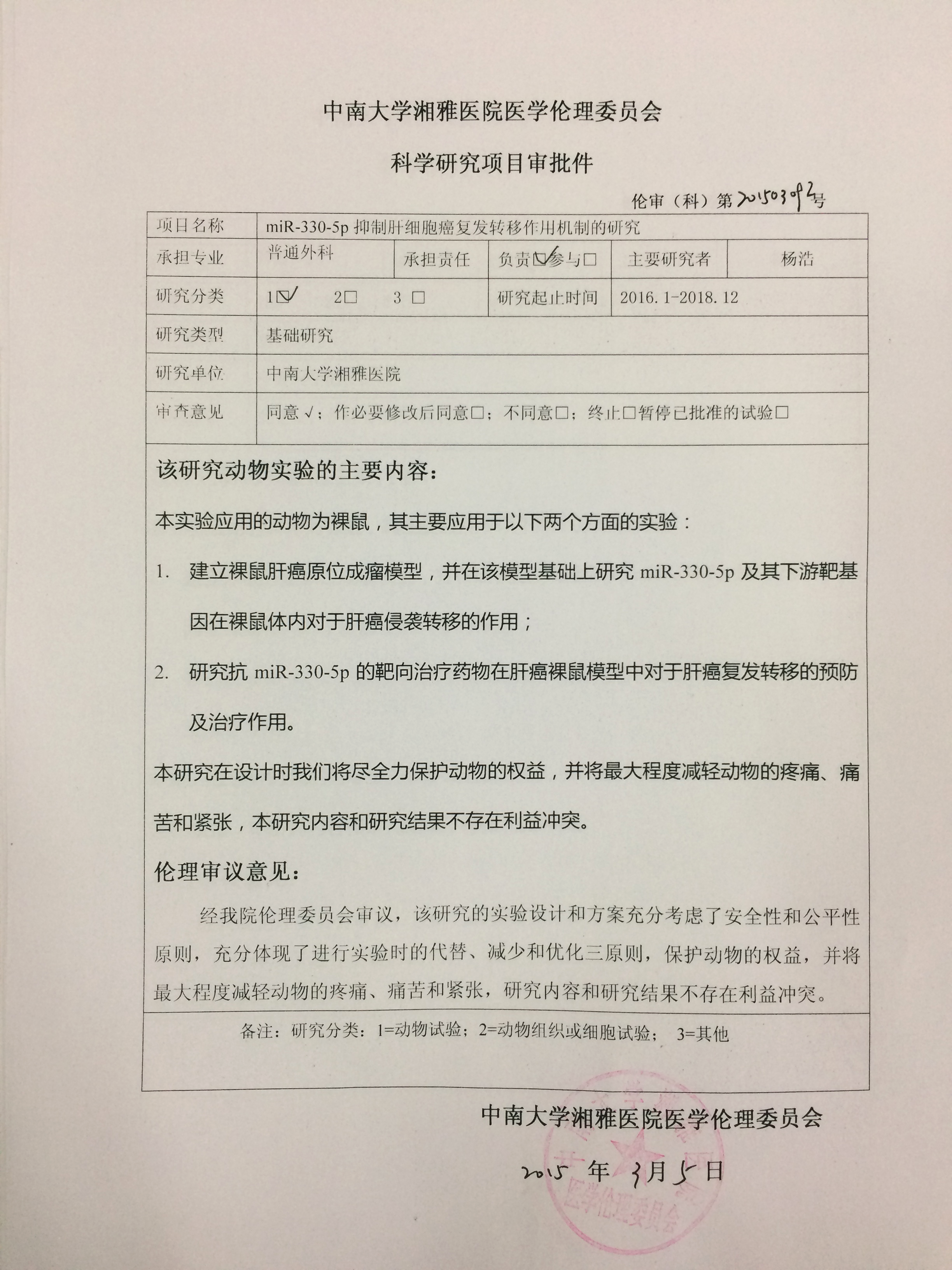

Supplement: Supplementary file 7 — Ethics approval [file 41389_2018_97_MOESM7_ESM.doc]
