# Supplementary material for: miR-330-5p targets SPRY2 to promote hepatocellular carcinoma progression via MAPK/ERK signaling
Source: Oncogenesis. 2018 Nov 21;7(11):90. doi: 10.1038/s41389-018-0097-8 (PMC6249243; doi:10.1038/s41389-018-0097-8)
Supplement: Supplementary file 5 — Supplementary Table S1 [file 41389_2018_97_MOESM5_ESM.doc]

**Supplementary Table S1** miR-330-5p involved signaling pathway

| **pathways** | **miRNAname** |
| --- | --- |
| hsa00010_Glycolysis Gluconeogenesis | hsa-miR-330-5p |
| hsa00030_Pentose phosphate pathway | hsa-miR-330-5p |
| hsa00051_Fructose and mannose metabolism | hsa-miR-330-5p |
| hsa00052_Galactose metabolism | hsa-miR-330-5p |
| hsa00230_Purine metabolism | hsa-miR-330-5p |
| hsa00510_N Glycan biosynthesis | hsa-miR-330-5p |
| hsa00514_O Mannosyl glycan biosynthesis | hsa-miR-330-5p |
| hsa01100_Metabolic pathways | hsa-miR-330-5p |
| hsa01100_Metabolic pathways | hsa-miR-330-5p |
| hsa03010_Ribosome | hsa-miR-330-5p |
| hsa03040_Spliceosome | hsa-miR-330-5p |
| hsa04010_MAPK signaling pathway | hsa-miR-330-5p |
| hsa04010_MAPK signaling pathway | hsa-miR-330-5p |
| hsa04012_ErbB signaling pathway | hsa-miR-330-5p |
| hsa04060_Cytokine cytokine receptor interaction | hsa-miR-330-5p |
| hsa04062_Chemokine signaling pathway | hsa-miR-330-5p |
| hsa04120_Ubiquitin mediated proteolysis | hsa-miR-330-5p |
| hsa04120_Ubiquitin mediated proteolysis | hsa-miR-330-5p |
| hsa04130_SNARE interactions in vesicular transport | hsa-miR-330-5p |
| hsa04144_Endocytosis | hsa-miR-330-5p |
| hsa04144_Endocytosis | hsa-miR-330-5p |
| hsa04210_Apoptosis | hsa-miR-330-5p |
| hsa04210_Apoptosis | hsa-miR-330-5p |
| hsa04370_VEGF signaling pathway | hsa-miR-330-5p |
| hsa04510_Focal adhesion | hsa-miR-330-5p |
| hsa04510_Focal adhesion | hsa-miR-330-5p |
| hsa04510_Focal adhesion | hsa-miR-330-5p |
| hsa04612_Antigen processing and presentation | hsa-miR-330-5p |
| hsa04620_Toll like receptor signaling pathway | hsa-miR-330-5p |
| hsa04630_Jak STAT signaling pathway | hsa-miR-330-5p |
| hsa04660_T cell receptor signaling pathway | hsa-miR-330-5p |
| hsa04662_B cell receptor signaling pathway | hsa-miR-330-5p |
| hsa04666_Fc gamma R mediated phagocytosis | hsa-miR-330-5p |
| hsa04670_Leukocyte transendothelial migration | hsa-miR-330-5p |
| hsa04722_Neurotrophin signaling pathway | hsa-miR-330-5p |
| hsa04722_Neurotrophin signaling pathway | hsa-miR-330-5p |
| hsa04722_Neurotrophin signaling pathway | hsa-miR-330-5p |
| hsa04740_Olfactory transduction | hsa-miR-330-5p |
| hsa04910_Insulin signaling pathway | hsa-miR-330-5p |
| hsa04910_Insulin signaling pathway | hsa-miR-330-5p |
| hsa04920_Adipocytokine signaling pathway | hsa-miR-330-5p |
| hsa04930_Type II diabetes mellitus | hsa-miR-330-5p |
| hsa04960_Aldosterone regulated sodium reabsorption | hsa-miR-330-5p |
| hsa05010_Alzheimers disease | hsa-miR-330-5p |
| hsa05014_Amyotrophic lateral sclerosis ALS | hsa-miR-330-5p |
| hsa05200_Pathways in cancer | hsa-miR-330-5p |
| hsa05210_Colorectal cancer | hsa-miR-330-5p |
| hsa05212_Pancreatic cancer | hsa-miR-330-5p |
| hsa05213_Endometrial cancer | hsa-miR-330-5p |
| hsa05215_Prostate cancer | hsa-miR-330-5p |
| hsa05218_Melanoma | hsa-miR-330-5p |
| hsa05220_Chronic myeloid leukemia | hsa-miR-330-5p |
| hsa05221_Acute myeloid leukemia | hsa-miR-330-5p |
| hsa05223_Non small cell lung cancer | hsa-miR-330-5p |
