# Supplementary material for: miR-330-5p targets SPRY2 to promote hepatocellular carcinoma progression via MAPK/ERK signaling
Source: Oncogenesis. 2018 Nov 21;7(11):90. doi: 10.1038/s41389-018-0097-8 (PMC6249243; doi:10.1038/s41389-018-0097-8)
Supplement: Supplementary file 6 — Supplementary Table S2 [file 41389_2018_97_MOESM6_ESM.doc]

**Supplementary Table S2** miR-330-5p predicted targets on mRNA 3' UTR region

| **MicroRNA** | **Gene** | **DIANAmT** | **miRanda** | **PICTAR5** | **Targetscan** | **SUM** |
| --- | --- | --- | --- | --- | --- | --- |
| hsa-miR-330-5p | ARPP-19 | 1 | 1 | 1 | 1 | 4 |
| hsa-miR-330-5p | RIMS4 | 1 | 1 | 1 | 1 | 4 |
| hsa-miR-330-5p | KCNIP2 | 1 | 1 | 1 | 1 | 4 |
| hsa-miR-330-5p | PDE3B | 1 | 1 | 1 | 1 | 4 |
| hsa-miR-330-5p | PRKCA | 1 | 1 | 1 | 1 | 4 |
| hsa-miR-330-5p | RAP2A | 1 | 1 | 1 | 1 | 4 |
| hsa-miR-330-5p | THBD | 1 | 1 | 1 | 1 | 4 |
| hsa-miR-330-5p | C22orf13 | 1 | 1 | 1 | 1 | 4 |
| hsa-miR-330-5p | PPP1R1B | 1 | 1 | 1 | 1 | 4 |
| hsa-miR-330-5p | SLC25A15 | 1 | 1 | 1 | 1 | 4 |
| hsa-miR-330-5p | WDR68 | 1 | 1 | 1 | 1 | 4 |
| hsa-miR-330-5p | SRRM1 | 1 | 1 | 1 | 1 | 4 |
| hsa-miR-330-5p | TRDN | 1 | 1 | 1 | 1 | 4 |
| hsa-miR-330-5p | SLC25A17 | 1 | 1 | 1 | 1 | 4 |
| hsa-miR-330-5p | ADARB2 | 1 | 1 | 1 | 1 | 4 |
| hsa-miR-330-5p | POLR3F | 1 | 1 | 1 | 1 | 4 |
| hsa-miR-330-5p | CTCF | 1 | 1 | 1 | 1 | 4 |
| hsa-miR-330-5p | ZNF275 | 1 | 1 | 1 | 1 | 4 |
| hsa-miR-330-5p | GPR83 | 1 | 1 | 1 | 1 | 4 |
| hsa-miR-330-5p | TOMM34 | 1 | 1 | 1 | 1 | 4 |
| hsa-miR-330-5p | SLC27A4 | 1 | 1 | 1 | 1 | 4 |
| hsa-miR-330-5p | RCBTB2 | 1 | 1 | 1 | 1 | 4 |
| hsa-miR-330-5p | BTN2A1 | 1 | 1 | 1 | 1 | 4 |
| hsa-miR-330-5p | ADAMTS6 | 1 | 1 | 1 | 1 | 4 |
| hsa-miR-330-5p | CDCA5 | 1 | 1 | 1 | 1 | 4 |
| hsa-miR-330-5p | SLC26A9 | 1 | 1 | 1 | 1 | 4 |
| hsa-miR-330-5p | SLC22A12 | 1 | 1 | 1 | 1 | 4 |
| hsa-miR-330-5p | CLCN6 | 1 | 1 | 1 | 1 | 4 |
| hsa-miR-330-5p | PAQR4 | 1 | 1 | 1 | 1 | 4 |
| hsa-miR-330-5p | USH1G | 1 | 1 | 1 | 1 | 4 |
| hsa-miR-330-5p | C1orf173 | 1 | 1 | 1 | 1 | 4 |
| hsa-miR-330-5p | MTPN | 1 | 1 | 1 | 1 | 4 |
| hsa-miR-330-5p | CREBBP | 1 | 1 | 1 | 1 | 4 |
| hsa-miR-330-5p | PASD1 | 1 | 1 | 1 | 1 | 4 |
| hsa-miR-330-5p | GAB3 | 1 | 1 | 1 | 1 | 4 |
| hsa-miR-330-5p | STK35 | 1 | 1 | 1 | 1 | 4 |
| hsa-miR-330-5p | VTI1A | 1 | 1 | 1 | 1 | 4 |
| hsa-miR-330-5p | SYT9 | 1 | 1 | 1 | 1 | 4 |
| hsa-miR-330-5p | UBQLNL | 1 | 1 | 1 | 1 | 4 |
| hsa-miR-330-5p | PRIMA1 | 1 | 1 | 1 | 1 | 4 |
| hsa-miR-330-5p | C15orf32 | 1 | 1 | 1 | 1 | 4 |
| hsa-miR-330-5p | NCAN | 1 | 1 | 1 | 1 | 4 |
| hsa-miR-330-5p | TOM1L2 | 1 | 1 | 1 | 1 | 4 |
| hsa-miR-330-5p | CTNS | 1 | 1 | 1 | 1 | 4 |
| hsa-miR-330-5p | IGSF5 | 1 | 1 | 1 | 1 | 4 |
| hsa-miR-330-5p | CMTM8 | 1 | 1 | 1 | 1 | 4 |
| hsa-miR-330-5p | AMOTL1 | 1 | 1 | 1 | 1 | 4 |
| hsa-miR-330-5p | DAG1 | 1 | 1 | 1 | 1 | 4 |
| hsa-miR-330-5p | LONRF2 | 1 | 1 | 1 | 1 | 4 |
| hsa-miR-330-5p | MIER3 | 1 | 1 | 1 | 1 | 4 |
| hsa-miR-330-5p | ASXL1 | 1 | 1 | 1 | 1 | 4 |
| hsa-miR-330-5p | DPYSL3 | 1 | 1 | 1 | 1 | 4 |
| hsa-miR-330-5p | E2F1 | 1 | 1 | 1 | 1 | 4 |
| hsa-miR-330-5p | EDA | 1 | 1 | 1 | 1 | 4 |
| hsa-miR-330-5p | EIF2C4 | 1 | 1 | 1 | 1 | 4 |
| hsa-miR-330-5p | EFNA3 | 1 | 1 | 1 | 1 | 4 |
| hsa-miR-330-5p | EFNB2 | 1 | 1 | 1 | 1 | 4 |
| hsa-miR-330-5p | C2orf13 | 1 | 1 | 1 | 1 | 4 |
| hsa-miR-330-5p | ZBTB7C | 1 | 1 | 1 | 1 | 4 |
| hsa-miR-330-5p | DNAJC18 | 1 | 1 | 1 | 1 | 4 |
| hsa-miR-330-5p | EPHX2 | 1 | 1 | 1 | 1 | 4 |
| hsa-miR-330-5p | ETF1 | 1 | 1 | 1 | 1 | 4 |
| hsa-miR-330-5p | PPM1E | 1 | 1 | 1 | 1 | 4 |
| hsa-miR-330-5p | LPHN1 | 1 | 1 | 1 | 1 | 4 |
| hsa-miR-330-5p | FNDC3A | 1 | 1 | 1 | 1 | 4 |
| hsa-miR-330-5p | BAHD1 | 1 | 1 | 1 | 1 | 4 |
| hsa-miR-330-5p | ZZEF1 | 1 | 1 | 1 | 1 | 4 |
| hsa-miR-330-5p | ARC | 1 | 1 | 1 | 1 | 4 |
| hsa-miR-330-5p | AFF2 | 1 | 1 | 1 | 1 | 4 |
| hsa-miR-330-5p | PRND | 1 | 1 | 1 | 1 | 4 |
| hsa-miR-330-5p | LDOC1 | 1 | 1 | 1 | 1 | 4 |
| hsa-miR-330-5p | APOL2 | 1 | 1 | 1 | 1 | 4 |
| hsa-miR-330-5p | MTCH1 | 1 | 1 | 1 | 1 | 4 |
| hsa-miR-330-5p | PANX1 | 1 | 1 | 1 | 1 | 4 |
| hsa-miR-330-5p | ALOX15B | 1 | 1 | 1 | 1 | 4 |
| hsa-miR-330-5p | ALPI | 1 | 1 | 1 | 1 | 4 |
| hsa-miR-330-5p | NSL1 | 1 | 1 | 1 | 1 | 4 |
| hsa-miR-330-5p | TIPARP | 1 | 1 | 1 | 1 | 4 |
| hsa-miR-330-5p | FBXW2 | 1 | 1 | 1 | 1 | 4 |
| hsa-miR-330-5p | FBXO10 | 1 | 1 | 1 | 1 | 4 |
| hsa-miR-330-5p | CNNM1 | 1 | 1 | 1 | 1 | 4 |
| hsa-miR-330-5p | B4GALT1 | 1 | 1 | 1 | 1 | 4 |
| hsa-miR-330-5p | GGT7 | 1 | 1 | 1 | 1 | 4 |
| hsa-miR-330-5p | KCNV1 | 1 | 1 | 1 | 1 | 4 |
| hsa-miR-330-5p | GLDC | 1 | 1 | 1 | 1 | 4 |
| hsa-miR-330-5p | BHLHB5 | 1 | 1 | 1 | 1 | 4 |
| hsa-miR-330-5p | PCDH11X | 1 | 1 | 1 | 1 | 4 |
| hsa-miR-330-5p | PTRF | 1 | 1 | 1 | 1 | 4 |
| hsa-miR-330-5p | SH3PXD2B | 1 | 1 | 1 | 1 | 4 |
| hsa-miR-330-5p | MED4 | 1 | 1 | 1 | 1 | 4 |
| hsa-miR-330-5p | GTF2H1 | 1 | 1 | 1 | 1 | 4 |
| hsa-miR-330-5p | GYS1 | 1 | 1 | 1 | 1 | 4 |
| hsa-miR-330-5p | EHD4 | 1 | 1 | 1 | 1 | 4 |
| hsa-miR-330-5p | HTR2C | 1 | 1 | 1 | 1 | 4 |
| hsa-miR-330-5p | IRF8 | 1 | 1 | 1 | 1 | 4 |
| hsa-miR-330-5p | ZKSCAN2 | 1 | 1 | 1 | 1 | 4 |
| hsa-miR-330-5p | LRRTM1 | 1 | 1 | 1 | 1 | 4 |
| hsa-miR-330-5p | IL10RA | 1 | 1 | 1 | 1 | 4 |
| hsa-miR-330-5p | KCNA7 | 1 | 1 | 1 | 1 | 4 |
| hsa-miR-330-5p | KRAS | 1 | 1 | 1 | 1 | 4 |
| hsa-miR-330-5p | SBK1 | 1 | 1 | 1 | 1 | 4 |
| hsa-miR-330-5p | FLJ34931 | 1 | 1 | 1 | 1 | 4 |
| hsa-miR-330-5p | PRAMEF12 | 1 | 1 | 1 | 1 | 4 |
| hsa-miR-330-5p | LEP | 1 | 1 | 1 | 1 | 4 |
| hsa-miR-330-5p | C10orf132 | 1 | 1 | 1 | 1 | 4 |
| hsa-miR-330-5p | ARSB | 1 | 1 | 1 | 1 | 4 |
| hsa-miR-330-5p | MAP1B | 1 | 1 | 1 | 1 | 4 |
| hsa-miR-330-5p | ABCC1 | 1 | 1 | 1 | 1 | 4 |
| hsa-miR-330-5p | MTM1 | 1 | 1 | 1 | 1 | 4 |
| hsa-miR-330-5p | MYO6 | 1 | 1 | 1 | 1 | 4 |
| hsa-miR-330-5p | NF2 | 1 | 1 | 1 | 1 | 4 |
| hsa-miR-330-5p | NGFR | 1 | 1 | 1 | 1 | 4 |
| hsa-miR-330-5p | NINJ1 | 1 | 1 | 1 | 1 | 4 |
| hsa-miR-330-5p | ATP2B2 | 1 | 1 | 1 | 1 | 4 |
| hsa-miR-330-5p | PAFAH1B1 | 1 | 1 | 1 | 1 | 4 |
| hsa-miR-330-5p | PALM | 1 | 1 | 1 | 1 | 4 |
| hsa-miR-330-5p | PCDH9 | 1 | 1 | 1 | 1 | 4 |
| hsa-miR-330-5p | C3orf18 | 1 | 1 | 1 | 1 | 4 |
| hsa-miR-330-5p | CRIM1 | 1 | 1 | 1 | 1 | 4 |
| hsa-miR-330-5p | PCTK2 | 1 | 1 | 1 | 1 | 4 |
| hsa-miR-330-5p | MEX3C | 1 | 1 | 1 | 1 | 4 |
| hsa-miR-330-5p | PEX5L | 1 | 1 | 1 | 1 | 4 |
| hsa-miR-330-5p | PDPK1 | 1 | 1 | 1 | 1 | 4 |
| hsa-miR-330-5p | NLK | 1 | 1 | 1 | 1 | 4 |
| hsa-miR-330-5p | PLXNA2 | 1 | 1 | 1 | 1 | 4 |
| hsa-miR-330-5p | CSNK1G1 | 1 | 1 | 1 | 1 | 4 |
| hsa-miR-330-5p | DNAJB12 | 1 | 1 | 1 | 1 | 4 |
| hsa-miR-330-5p | RNF43 | 1 | 1 | 1 | 1 | 4 |
| hsa-miR-330-5p | UBE2R2 | 1 | 1 | 1 | 1 | 4 |
| hsa-miR-330-5p | C9orf68 | 1 | 1 | 1 | 1 | 4 |
| hsa-miR-330-5p | SOBP | 1 | 1 | 1 | 1 | 4 |
| hsa-miR-330-5p | SAMD4B | 1 | 1 | 1 | 1 | 4 |
| hsa-miR-330-5p | BSDC1 | 1 | 1 | 1 | 1 | 4 |
| hsa-miR-330-5p | CDCA8 | 1 | 1 | 1 | 1 | 4 |
| hsa-miR-330-5p | QRSL1 | 1 | 1 | 1 | 1 | 4 |
| hsa-miR-330-5p | RHOT1 | 1 | 1 | 1 | 1 | 4 |
| hsa-miR-330-5p | SLC22A15 | 1 | 1 | 1 | 1 | 4 |
| hsa-miR-330-5p | KLHL9 | 1 | 1 | 1 | 1 | 4 |
| hsa-miR-330-5p | MAPK4 | 1 | 1 | 1 | 1 | 4 |
| hsa-miR-330-5p | RP5-1022P6.2 | 1 | 1 | 1 | 1 | 4 |
| hsa-miR-330-5p | MDM1 | 1 | 1 | 1 | 1 | 4 |
| hsa-miR-330-5p | ZNF248 | 1 | 1 | 1 | 1 | 4 |
| hsa-miR-330-5p | CASKIN1 | 1 | 1 | 1 | 1 | 4 |
| hsa-miR-330-5p | WDFY1 | 1 | 1 | 1 | 1 | 4 |
| hsa-miR-330-5p | PVRL2 | 1 | 1 | 1 | 1 | 4 |
| hsa-miR-330-5p | RIT1 | 1 | 1 | 1 | 1 | 4 |
| hsa-miR-330-5p | ABHD4 | 1 | 1 | 1 | 1 | 4 |
| hsa-miR-330-5p | UBE2O | 1 | 1 | 1 | 1 | 4 |
| hsa-miR-330-5p | GOLPH3 | 1 | 1 | 1 | 1 | 4 |
| hsa-miR-330-5p | SMOC2 | 1 | 1 | 1 | 1 | 4 |
| hsa-miR-330-5p | XPO4 | 1 | 1 | 1 | 1 | 4 |
| hsa-miR-330-5p | ACBD3 | 1 | 1 | 1 | 1 | 4 |
| hsa-miR-330-5p | CRTC3 | 1 | 1 | 1 | 1 | 4 |
| hsa-miR-330-5p | CCDC21 | 1 | 1 | 1 | 1 | 4 |
| hsa-miR-330-5p | BCL11B | 1 | 1 | 1 | 1 | 4 |
| hsa-miR-330-5p | REEP1 | 1 | 1 | 1 | 1 | 4 |
| hsa-miR-330-5p | SLC2A1 | 1 | 1 | 1 | 1 | 4 |
| hsa-miR-330-5p | SLC5A3 | 1 | 1 | 1 | 1 | 4 |
| hsa-miR-330-5p | SLC8A2 | 1 | 1 | 1 | 1 | 4 |
| hsa-miR-330-5p | TRAK2 | 1 | 1 | 1 | 1 | 4 |
| hsa-miR-330-5p | SPOCK1 | 1 | 1 | 1 | 1 | 4 |
| hsa-miR-330-5p | SRPR | 1 | 1 | 1 | 1 | 4 |
| hsa-miR-330-5p | STAU1 | 1 | 1 | 1 | 1 | 4 |
| hsa-miR-330-5p | TUFT1 | 1 | 1 | 1 | 1 | 4 |
| hsa-miR-330-5p | TYR | 1 | 1 | 1 | 1 | 4 |
| hsa-miR-330-5p | VLDLR | 1 | 1 | 1 | 1 | 4 |
| hsa-miR-330-5p | WNT7B | 1 | 1 | 1 | 1 | 4 |
| hsa-miR-330-5p | ZFY | 1 | 1 | 1 | 1 | 4 |
| hsa-miR-330-5p | PAX8 | 1 | 1 | 1 | 1 | 4 |
| hsa-miR-330-5p | CCDC86 | 1 | 1 | 1 | 1 | 4 |
| hsa-miR-330-5p | ALDH5A1 | 1 | 1 | 1 | 1 | 4 |
| hsa-miR-330-5p | ZNF343 | 1 | 1 | 1 | 1 | 4 |
| hsa-miR-330-5p | WDR32 | 1 | 1 | 1 | 1 | 4 |
| hsa-miR-330-5p | TMCO7 | 1 | 1 | 1 | 1 | 4 |
| hsa-miR-330-5p | C22orf29 | 1 | 1 | 1 | 1 | 4 |
| hsa-miR-330-5p | STEAP4 | 1 | 1 | 1 | 1 | 4 |
| hsa-miR-330-5p | ARMC5 | 1 | 1 | 1 | 1 | 4 |
| hsa-miR-330-5p | PIP4K2C | 1 | 1 | 1 | 1 | 4 |
| hsa-miR-330-5p | FLJ12529 | 1 | 1 | 1 | 1 | 4 |
| hsa-miR-330-5p | CBLL1 | 1 | 1 | 1 | 1 | 4 |
| hsa-miR-330-5p | CALCR | 1 | 1 | 1 | 1 | 4 |
| hsa-miR-330-5p | ADM2 | 1 | 1 | 1 | 1 | 4 |
| hsa-miR-330-5p | FRAS1 | 1 | 1 | 1 | 1 | 4 |
| hsa-miR-330-5p | PDGFD | 1 | 1 | 1 | 1 | 4 |
| hsa-miR-330-5p | TSPAN14 | 1 | 1 | 1 | 1 | 4 |
| hsa-miR-330-5p | PCDH11Y | 1 | 1 | 1 | 1 | 4 |
| hsa-miR-330-5p | TOMM40L | 1 | 1 | 1 | 1 | 4 |
| hsa-miR-330-5p | LZTS2 | 1 | 1 | 1 | 1 | 4 |
| hsa-miR-330-5p | HAVCR2 | 1 | 1 | 1 | 1 | 4 |
| hsa-miR-330-5p | SPRYD3 | 1 | 1 | 1 | 1 | 4 |
| hsa-miR-330-5p | FBXL20 | 1 | 1 | 1 | 1 | 4 |
| hsa-miR-330-5p | SELI | 1 | 1 | 1 | 1 | 4 |
| hsa-miR-330-5p | TCAP | 1 | 1 | 1 | 1 | 4 |
| hsa-miR-330-5p | HDAC3 | 1 | 1 | 1 | 1 | 4 |
| hsa-miR-330-5p | PER3 | 1 | 1 | 1 | 1 | 4 |
| hsa-miR-330-5p | SGPL1 | 1 | 1 | 1 | 1 | 4 |
| hsa-miR-330-5p | BTRC | 1 | 1 | 1 | 1 | 4 |
| hsa-miR-330-5p | PPP1R3F | 1 | 1 | 1 | 1 | 4 |
| hsa-miR-330-5p | KLHL6 | 1 | 1 | 1 | 1 | 4 |
| hsa-miR-330-5p | C16orf45 | 1 | 1 | 1 | 1 | 4 |
| hsa-miR-330-5p | PRPF4 | 1 | 1 | 1 | 1 | 4 |
| hsa-miR-330-5p | CD3G | 1 | 1 | 1 | 1 | 4 |
| hsa-miR-330-5p | RFT1 | 1 | 1 | 1 | 1 | 4 |
| hsa-miR-330-5p | DCLK1 | 1 | 1 | 1 | 1 | 4 |
| hsa-miR-330-5p | SNX29 | 1 | 1 | 1 | 1 | 4 |
| hsa-miR-330-5p | CD6 | 1 | 1 | 1 | 1 | 4 |
| hsa-miR-330-5p | PSCD3 | 1 | 1 | 1 | 1 | 4 |
| hsa-miR-330-5p | MRFAP1 | 1 | 1 | 1 | 1 | 4 |
| hsa-miR-330-5p | ADIPOQ | 1 | 1 | 1 | 1 | 4 |
| hsa-miR-330-5p | H6PD | 1 | 1 | 1 | 1 | 4 |
| hsa-miR-330-5p | CD47 | 1 | 1 | 1 | 1 | 4 |
| hsa-miR-330-5p | SH3PXD2A | 1 | 1 | 1 | 1 | 4 |
| hsa-miR-330-5p | SEC14L5 | 1 | 1 | 1 | 1 | 4 |
| hsa-miR-330-5p | VPRBP | 1 | 1 | 1 | 1 | 4 |
| hsa-miR-330-5p | TOX | 1 | 1 | 1 | 1 | 4 |
| hsa-miR-330-5p | KIAA0319 | 1 | 1 | 1 | 1 | 4 |
| hsa-miR-330-5p | SMG7 | 1 | 1 | 1 | 1 | 4 |
| hsa-miR-330-5p | RBM19 | 1 | 1 | 1 | 1 | 4 |
| hsa-miR-330-5p | ARNT2 | 1 | 1 | 1 | 1 | 4 |
| hsa-miR-330-5p | OXSR1 | 1 | 1 | 1 | 1 | 4 |
| hsa-miR-330-5p | SLC23A2 | 1 | 1 | 1 | 1 | 4 |
| hsa-miR-330-5p | AKT3 | 1 | 1 | 1 | 1 | 4 |
| hsa-miR-330-5p | NR2E3 | 1 | 1 | 1 | 1 | 4 |
| hsa-miR-330-5p | GNPDA1 | 1 | 1 | 1 | 1 | 4 |
| hsa-miR-330-5p | SH2B3 | 1 | 1 | 1 | 1 | 4 |
| hsa-miR-330-5p | HCN4 | 1 | 1 | 1 | 1 | 4 |
| hsa-miR-330-5p | CDH5 | 1 | 1 | 1 | 1 | 4 |
| hsa-miR-330-5p | SH2D3C | 1 | 1 | 1 | 1 | 4 |
| hsa-miR-330-5p | DNAJB6 | 1 | 1 | 1 | 1 | 4 |
| hsa-miR-330-5p | AP1M2 | 1 | 1 | 1 | 1 | 4 |
| hsa-miR-330-5p | PPIF | 1 | 1 | 1 | 1 | 4 |
| hsa-miR-330-5p | CTDSP2 | 1 | 1 | 1 | 1 | 4 |
| hsa-miR-330-5p | CDH16 | 1 | 1 | 1 | 1 | 4 |
| hsa-miR-330-5p | FAM13A1 | 1 | 1 | 1 | 1 | 4 |
| hsa-miR-330-5p | ABI2 | 1 | 1 | 1 | 1 | 4 |
| hsa-miR-330-5p | WASF2 | 1 | 1 | 1 | 1 | 4 |
| hsa-miR-330-5p | LHFPL2 | 1 | 1 | 1 | 1 | 4 |
| hsa-miR-330-5p | CALCRL | 1 | 1 | 1 | 1 | 4 |
| hsa-miR-330-5p | INADL | 1 | 1 | 1 | 1 | 4 |
| hsa-miR-330-5p | EIF1 | 1 | 1 | 1 | 1 | 4 |
| hsa-miR-330-5p | TRIB1 | 1 | 1 | 1 | 1 | 4 |
| hsa-miR-330-5p | STX6 | 1 | 1 | 1 | 1 | 4 |
| hsa-miR-330-5p | RCAN2 | 1 | 1 | 1 | 1 | 4 |
| hsa-miR-330-5p | CALCOCO2 | 1 | 1 | 1 | 1 | 4 |
| hsa-miR-330-5p | TIMM17B | 1 | 1 | 1 | 1 | 4 |
| hsa-miR-330-5p | SLC17A2 | 1 | 1 | 1 | 1 | 4 |
| hsa-miR-330-5p | CDK9 | 1 | 1 | 1 | 1 | 4 |
| hsa-miR-330-5p | UBE4B | 1 | 1 | 1 | 1 | 4 |
| hsa-miR-330-5p | CDKN2A | 1 | 1 | 1 | 1 | 4 |
| hsa-miR-330-5p | TRAIP | 1 | 1 | 1 | 1 | 4 |
| hsa-miR-330-5p | APC2 | 1 | 1 | 1 | 1 | 4 |
| hsa-miR-330-5p | LAMC3 | 1 | 1 | 1 | 1 | 4 |
| hsa-miR-330-5p | SMYD5 | 1 | 1 | 1 | 1 | 4 |
| hsa-miR-330-5p | PCGF3 | 1 | 1 | 1 | 1 | 4 |
| hsa-miR-330-5p | CCL26 | 1 | 1 | 1 | 1 | 4 |
| hsa-miR-330-5p | KLF2 | 1 | 1 | 1 | 1 | 4 |
| hsa-miR-330-5p | CORO2B | 1 | 1 | 1 | 1 | 4 |
| hsa-miR-330-5p | ATP8A1 | 1 | 1 | 1 | 1 | 4 |
| hsa-miR-330-5p | UBAC1 | 1 | 1 | 1 | 1 | 4 |
| hsa-miR-330-5p | CDC42EP2 | 1 | 1 | 1 | 1 | 4 |
| hsa-miR-330-5p | CDX1 | 1 | 1 | 1 | 1 | 4 |
| hsa-miR-330-5p | CEACAM5 | 1 | 1 | 1 | 1 | 4 |
| hsa-miR-330-5p | HOXB13 | 1 | 1 | 1 | 1 | 4 |
| hsa-miR-330-5p | CRTAP | 1 | 1 | 1 | 1 | 4 |
| hsa-miR-330-5p | STK25 | 1 | 1 | 1 | 1 | 4 |
| hsa-miR-330-5p | ENOX2 | 1 | 1 | 1 | 1 | 4 |
| hsa-miR-330-5p | SEMA6C | 1 | 1 | 1 | 1 | 4 |
| hsa-miR-330-5p | ZNF211 | 1 | 1 | 1 | 1 | 4 |
| hsa-miR-330-5p | SPTLC1 | 1 | 1 | 1 | 1 | 4 |
| hsa-miR-330-5p | SLC19A2 | 1 | 1 | 1 | 1 | 4 |
| hsa-miR-330-5p | MRPL28 | 1 | 1 | 1 | 1 | 4 |
| hsa-miR-330-5p | IFITM2 | 1 | 1 | 1 | 1 | 4 |
| hsa-miR-330-5p | CENPB | 1 | 1 | 1 | 1 | 4 |
| hsa-miR-330-5p | CDC42EP3 | 1 | 1 | 1 | 1 | 4 |
| hsa-miR-330-5p | IGF2BP1 | 1 | 1 | 1 | 1 | 4 |
| hsa-miR-330-5p | IGF2BP2 | 1 | 1 | 1 | 1 | 4 |
| hsa-miR-330-5p | PMVK | 1 | 1 | 1 | 1 | 4 |
| hsa-miR-330-5p | CUGBP2 | 1 | 1 | 1 | 1 | 4 |
| hsa-miR-330-5p | KLF1 | 1 | 1 | 1 | 1 | 4 |
| hsa-miR-330-5p | CNPY3 | 1 | 1 | 1 | 1 | 4 |
| hsa-miR-330-5p | ADCY1 | 1 | 1 | 1 | 1 | 4 |
| hsa-miR-330-5p | POLQ | 1 | 1 | 1 | 1 | 4 |
| hsa-miR-330-5p | SLC12A7 | 1 | 1 | 1 | 1 | 4 |
| hsa-miR-330-5p | MAP3K2 | 1 | 1 | 1 | 1 | 4 |
| hsa-miR-330-5p | GRAP | 1 | 1 | 1 | 1 | 4 |
| hsa-miR-330-5p | NES | 1 | 1 | 1 | 1 | 4 |
| hsa-miR-330-5p | AHCYL1 | 1 | 1 | 1 | 1 | 4 |
| hsa-miR-330-5p | ZNF273 | 1 | 1 | 1 | 1 | 4 |
| hsa-miR-330-5p | CCR9 | 1 | 1 | 1 | 1 | 4 |
| hsa-miR-330-5p | SDCCAG3 | 1 | 1 | 1 | 1 | 4 |
| hsa-miR-330-5p | FRS3 | 1 | 1 | 1 | 1 | 4 |
| hsa-miR-330-5p | C5orf3 | 1 | 1 | 1 | 1 | 4 |
| hsa-miR-330-5p | CEACAM3 | 1 | 1 | 1 | 1 | 4 |
| hsa-miR-330-5p | CD3EAP | 1 | 1 | 1 | 1 | 4 |
| hsa-miR-330-5p | ARID5A | 1 | 1 | 1 | 1 | 4 |
| hsa-miR-330-5p | TSPAN9 | 1 | 1 | 1 | 1 | 4 |
| hsa-miR-330-5p | ME3 | 1 | 1 | 1 | 1 | 4 |
| hsa-miR-330-5p | CEACAM4 | 1 | 1 | 1 | 1 | 4 |
| hsa-miR-330-5p | MMP24 | 1 | 1 | 1 | 1 | 4 |
| hsa-miR-330-5p | SPIN1 | 1 | 1 | 1 | 1 | 4 |
| hsa-miR-330-5p | AFG3L2 | 1 | 1 | 1 | 1 | 4 |
| hsa-miR-330-5p | MSL3L1 | 1 | 1 | 1 | 1 | 4 |
| hsa-miR-330-5p | KDELR1 | 1 | 1 | 1 | 1 | 4 |
| hsa-miR-330-5p | UQCR | 1 | 1 | 1 | 1 | 4 |
| hsa-miR-330-5p | SDS | 1 | 1 | 1 | 1 | 4 |
| hsa-miR-330-5p | KIF2C | 1 | 1 | 1 | 1 | 4 |
| hsa-miR-330-5p | CYB561D2 | 1 | 1 | 1 | 1 | 4 |
| hsa-miR-330-5p | TPPP | 1 | 1 | 1 | 1 | 4 |
| hsa-miR-330-5p | TRIOBP | 1 | 1 | 1 | 1 | 4 |
| hsa-miR-330-5p | C9orf7 | 1 | 1 | 1 | 1 | 4 |
| hsa-miR-330-5p | PRSS23 | 1 | 1 | 1 | 1 | 4 |
| hsa-miR-330-5p | CIT | 1 | 1 | 1 | 1 | 4 |
| hsa-miR-330-5p | EMILIN1 | 1 | 1 | 1 | 1 | 4 |
| hsa-miR-330-5p | PTPRT | 1 | 1 | 1 | 1 | 4 |
| hsa-miR-330-5p | CDC42EP1 | 1 | 1 | 1 | 1 | 4 |
| hsa-miR-330-5p | ERLIN2 | 1 | 1 | 1 | 1 | 4 |
| hsa-miR-330-5p | C14orf1 | 1 | 1 | 1 | 1 | 4 |
| hsa-miR-330-5p | FSTL1 | 1 | 1 | 1 | 1 | 4 |
| hsa-miR-330-5p | BAZ1A | 1 | 1 | 1 | 1 | 4 |
| hsa-miR-330-5p | LZTS1 | 1 | 1 | 1 | 1 | 4 |
| hsa-miR-330-5p | MAP4K5 | 1 | 1 | 1 | 1 | 4 |
| hsa-miR-330-5p | WIF1 | 1 | 1 | 1 | 1 | 4 |
| hsa-miR-330-5p | AKAP13 | 1 | 1 | 1 | 1 | 4 |
| hsa-miR-330-5p | PADI2 | 1 | 1 | 1 | 1 | 4 |
| hsa-miR-330-5p | C14orf126 | 1 | 1 | 1 | 1 | 4 |
| hsa-miR-330-5p | FAM71E1 | 1 | 1 | 1 | 1 | 4 |
| hsa-miR-330-5p | KLF12 | 1 | 1 | 1 | 1 | 4 |
| hsa-miR-330-5p | CHRM1 | 1 | 1 | 1 | 1 | 4 |
| hsa-miR-330-5p | FDX1L | 1 | 1 | 1 | 1 | 4 |
| hsa-miR-330-5p | CYP4F8 | 1 | 1 | 1 | 1 | 4 |
| hsa-miR-330-5p | BTBD14B | 1 | 1 | 1 | 1 | 4 |
| hsa-miR-330-5p | PLCD3 | 1 | 1 | 1 | 1 | 4 |
| hsa-miR-330-5p | VPS45 | 1 | 1 | 1 | 1 | 4 |
| hsa-miR-330-5p | AHNAK2 | 1 | 1 | 1 | 1 | 4 |
| hsa-miR-330-5p | MGAT4A | 1 | 1 | 1 | 1 | 4 |
| hsa-miR-330-5p | SLC46A1 | 1 | 1 | 1 | 1 | 4 |
| hsa-miR-330-5p | C20orf54 | 1 | 1 | 1 | 1 | 4 |
| hsa-miR-330-5p | TEX261 | 1 | 1 | 1 | 1 | 4 |
| hsa-miR-330-5p | CHRNA4 | 1 | 1 | 1 | 1 | 4 |
| hsa-miR-330-5p | PIK3IP1 | 1 | 1 | 1 | 1 | 4 |
| hsa-miR-330-5p | SLC35A4 | 1 | 1 | 1 | 1 | 4 |
| hsa-miR-330-5p | CHRNB2 | 1 | 1 | 1 | 1 | 4 |
| hsa-miR-330-5p | MAL2 | 1 | 1 | 1 | 1 | 4 |
| hsa-miR-330-5p | TIRAP | 1 | 1 | 1 | 1 | 4 |
| hsa-miR-330-5p | GPRIN1 | 1 | 1 | 1 | 1 | 4 |
| hsa-miR-330-5p | SLC25A25 | 1 | 1 | 1 | 1 | 4 |
| hsa-miR-330-5p | GALNT13 | 1 | 1 | 1 | 1 | 4 |
| hsa-miR-330-5p | RHPN1 | 1 | 1 | 1 | 1 | 4 |
| hsa-miR-330-5p | SMYD4 | 1 | 1 | 1 | 1 | 4 |
| hsa-miR-330-5p | OSBPL7 | 1 | 1 | 1 | 1 | 4 |
| hsa-miR-330-5p | C1QTNF1 | 1 | 1 | 1 | 1 | 4 |
| hsa-miR-330-5p | C1QTNF3 | 1 | 1 | 1 | 1 | 4 |
| hsa-miR-330-5p | C1QTNF7 | 1 | 1 | 1 | 1 | 4 |
| hsa-miR-330-5p | ADCY9 | 1 | 1 | 1 | 1 | 4 |
| hsa-miR-330-5p | NT5C3L | 1 | 1 | 1 | 1 | 4 |
| hsa-miR-330-5p | KCTD12 | 1 | 1 | 1 | 1 | 4 |
| hsa-miR-330-5p | SLC25A26 | 1 | 1 | 1 | 1 | 4 |
| hsa-miR-330-5p | FCHO2 | 1 | 1 | 1 | 1 | 4 |
| hsa-miR-330-5p | NXNL1 | 1 | 1 | 1 | 1 | 4 |
| hsa-miR-330-5p | ZNF653 | 1 | 1 | 1 | 1 | 4 |
| hsa-miR-330-5p | RNF166 | 1 | 1 | 1 | 1 | 4 |
| hsa-miR-330-5p | CMTM5 | 1 | 1 | 1 | 1 | 4 |
| hsa-miR-330-5p | LOC116236 | 1 | 1 | 1 | 1 | 4 |
| hsa-miR-330-5p | PANX3 | 1 | 1 | 1 | 1 | 4 |
| hsa-miR-330-5p | SLC26A8 | 1 | 1 | 1 | 1 | 4 |
| hsa-miR-330-5p | C1orf19 | 1 | 1 | 1 | 1 | 4 |
| hsa-miR-330-5p | HSPA12B | 1 | 1 | 1 | 1 | 4 |
| hsa-miR-330-5p | MED12L | 1 | 1 | 1 | 1 | 4 |
| hsa-miR-330-5p | SH2D1B | 1 | 1 | 1 | 1 | 4 |
| hsa-miR-330-5p | TMC1 | 1 | 1 | 1 | 1 | 4 |
| hsa-miR-330-5p | CLCN4 | 1 | 1 | 1 | 1 | 4 |
| hsa-miR-330-5p | TTC18 | 1 | 1 | 1 | 1 | 4 |
| hsa-miR-330-5p | CLCN7 | 1 | 1 | 1 | 1 | 4 |
| hsa-miR-330-5p | C10orf90 | 1 | 1 | 1 | 1 | 4 |
| hsa-miR-330-5p | ZNF488 | 1 | 1 | 1 | 1 | 4 |
| hsa-miR-330-5p | PIK3AP1 | 1 | 1 | 1 | 1 | 4 |
| hsa-miR-330-5p | C10orf4 | 1 | 1 | 1 | 1 | 4 |
| hsa-miR-330-5p | C10orf104 | 1 | 1 | 1 | 1 | 4 |
| hsa-miR-330-5p | SFXN4 | 1 | 1 | 1 | 1 | 4 |
| hsa-miR-330-5p | CLN3 | 1 | 1 | 1 | 1 | 4 |
| hsa-miR-330-5p | BTBD11 | 1 | 1 | 1 | 1 | 4 |
| hsa-miR-330-5p | FOXN4 | 1 | 1 | 1 | 1 | 4 |
| hsa-miR-330-5p | SLAIN1 | 1 | 1 | 1 | 1 | 4 |
| hsa-miR-330-5p | C14orf79 | 1 | 1 | 1 | 1 | 4 |
| hsa-miR-330-5p | CCR1 | 1 | 1 | 1 | 1 | 4 |
| hsa-miR-330-5p | SLC24A4 | 1 | 1 | 1 | 1 | 4 |
| hsa-miR-330-5p | NIPA1 | 1 | 1 | 1 | 1 | 4 |
| hsa-miR-330-5p | C16orf55 | 1 | 1 | 1 | 1 | 4 |
| hsa-miR-330-5p | ZC3H18 | 1 | 1 | 1 | 1 | 4 |
| hsa-miR-330-5p | CDYL2 | 1 | 1 | 1 | 1 | 4 |
| hsa-miR-330-5p | ANKS3 | 1 | 1 | 1 | 1 | 4 |
| hsa-miR-330-5p | CD300LB | 1 | 1 | 1 | 1 | 4 |
| hsa-miR-330-5p | ZPBP2 | 1 | 1 | 1 | 1 | 4 |
| hsa-miR-330-5p | CYB5D1 | 1 | 1 | 1 | 1 | 4 |
| hsa-miR-330-5p | KRBA2 | 1 | 1 | 1 | 1 | 4 |
| hsa-miR-330-5p | TMEM132E | 1 | 1 | 1 | 1 | 4 |
| hsa-miR-330-5p | SEZ6 | 1 | 1 | 1 | 1 | 4 |
| hsa-miR-330-5p | ANKRD13B | 1 | 1 | 1 | 1 | 4 |
| hsa-miR-330-5p | RAVER1 | 1 | 1 | 1 | 1 | 4 |
| hsa-miR-330-5p | COX6B2 | 1 | 1 | 1 | 1 | 4 |
| hsa-miR-330-5p | ZNF491 | 1 | 1 | 1 | 1 | 4 |
| hsa-miR-330-5p | LOC126075 | 1 | 1 | 1 | 1 | 4 |
| hsa-miR-330-5p | ZNF787 | 1 | 1 | 1 | 1 | 4 |
| hsa-miR-330-5p | CNN1 | 1 | 1 | 1 | 1 | 4 |
| hsa-miR-330-5p | FBXO27 | 1 | 1 | 1 | 1 | 4 |
| hsa-miR-330-5p | CNP | 1 | 1 | 1 | 1 | 4 |
| hsa-miR-330-5p | CNR2 | 1 | 1 | 1 | 1 | 4 |
| hsa-miR-330-5p | TYW3 | 1 | 1 | 1 | 1 | 4 |
| hsa-miR-330-5p | C1orf93 | 1 | 1 | 1 | 1 | 4 |
| hsa-miR-330-5p | C1orf216 | 1 | 1 | 1 | 1 | 4 |
| hsa-miR-330-5p | UBXD3 | 1 | 1 | 1 | 1 | 4 |
| hsa-miR-330-5p | ARL8A | 1 | 1 | 1 | 1 | 4 |
| hsa-miR-330-5p | IQGAP3 | 1 | 1 | 1 | 1 | 4 |
| hsa-miR-330-5p | ZNF831 | 1 | 1 | 1 | 1 | 4 |
| hsa-miR-330-5p | TBC1D20 | 1 | 1 | 1 | 1 | 4 |
| hsa-miR-330-5p | COL4A6 | 1 | 1 | 1 | 1 | 4 |
| hsa-miR-330-5p | GAB4 | 1 | 1 | 1 | 1 | 4 |
| hsa-miR-330-5p | SGSM1 | 1 | 1 | 1 | 1 | 4 |
| hsa-miR-330-5p | TAF8 | 1 | 1 | 1 | 1 | 4 |
| hsa-miR-330-5p | ICA1L | 1 | 1 | 1 | 1 | 4 |
| hsa-miR-330-5p | COL12A1 | 1 | 1 | 1 | 1 | 4 |
| hsa-miR-330-5p | PQLC3 | 1 | 1 | 1 | 1 | 4 |
| hsa-miR-330-5p | FBXO36 | 1 | 1 | 1 | 1 | 4 |
| hsa-miR-330-5p | COPA | 1 | 1 | 1 | 1 | 4 |
| hsa-miR-330-5p | DCBLD2 | 1 | 1 | 1 | 1 | 4 |
| hsa-miR-330-5p | UROC1 | 1 | 1 | 1 | 1 | 4 |
| hsa-miR-330-5p | SLC31A1 | 1 | 1 | 1 | 1 | 4 |
| hsa-miR-330-5p | TMEM207 | 1 | 1 | 1 | 1 | 4 |
| hsa-miR-330-5p | TMEM155 | 1 | 1 | 1 | 1 | 4 |
| hsa-miR-330-5p | TMPRSS11B | 1 | 1 | 1 | 1 | 4 |
| hsa-miR-330-5p | C4orf36 | 1 | 1 | 1 | 1 | 4 |
| hsa-miR-330-5p | PRRC1 | 1 | 1 | 1 | 1 | 4 |
| hsa-miR-330-5p | C5orf33 | 1 | 1 | 1 | 1 | 4 |
| hsa-miR-330-5p | CMBL | 1 | 1 | 1 | 1 | 4 |
| hsa-miR-330-5p | GRPEL2 | 1 | 1 | 1 | 1 | 4 |
| hsa-miR-330-5p | WDR36 | 1 | 1 | 1 | 1 | 4 |
| hsa-miR-330-5p | ANKRD43 | 1 | 1 | 1 | 1 | 4 |
| hsa-miR-330-5p | ADAT2 | 1 | 1 | 1 | 1 | 4 |
| hsa-miR-330-5p | ADORA2A | 1 | 1 | 1 | 1 | 4 |
| hsa-miR-330-5p | CD109 | 1 | 1 | 1 | 1 | 4 |
| hsa-miR-330-5p | C7orf34 | 1 | 1 | 1 | 1 | 4 |
| hsa-miR-330-5p | TMEM139 | 1 | 1 | 1 | 1 | 4 |
| hsa-miR-330-5p | CLDN4 | 1 | 1 | 1 | 1 | 4 |
| hsa-miR-330-5p | CPOX | 1 | 1 | 1 | 1 | 4 |
| hsa-miR-330-5p | LOC137886 | 1 | 1 | 1 | 1 | 4 |
| hsa-miR-330-5p | CRABP2 | 1 | 1 | 1 | 1 | 4 |
| hsa-miR-330-5p | CRHR1 | 1 | 1 | 1 | 1 | 4 |
| hsa-miR-330-5p | CRKL | 1 | 1 | 1 | 1 | 4 |
| hsa-miR-330-5p | ASB6 | 1 | 1 | 1 | 1 | 4 |
| hsa-miR-330-5p | GATA5 | 1 | 1 | 1 | 1 | 4 |
| hsa-miR-330-5p | ZBTB46 | 1 | 1 | 1 | 1 | 4 |
| hsa-miR-330-5p | SAMD10 | 1 | 1 | 1 | 1 | 4 |
| hsa-miR-330-5p | ADAMTS14 | 1 | 1 | 1 | 1 | 4 |
| hsa-miR-330-5p | CRY2 | 1 | 1 | 1 | 1 | 4 |
| hsa-miR-330-5p | C20orf175 | 1 | 1 | 1 | 1 | 4 |
| hsa-miR-330-5p | DUSP19 | 1 | 1 | 1 | 1 | 4 |
| hsa-miR-330-5p | CSF1 | 1 | 1 | 1 | 1 | 4 |
| hsa-miR-330-5p | TMEM86A | 1 | 1 | 1 | 1 | 4 |
| hsa-miR-330-5p | FAM101A | 1 | 1 | 1 | 1 | 4 |
| hsa-miR-330-5p | GLT1D1 | 1 | 1 | 1 | 1 | 4 |
| hsa-miR-330-5p | KRT80 | 1 | 1 | 1 | 1 | 4 |
| hsa-miR-330-5p | C13orf30 | 1 | 1 | 1 | 1 | 4 |
| hsa-miR-330-5p | CSNK1G3 | 1 | 1 | 1 | 1 | 4 |
| hsa-miR-330-5p | LYSMD4 | 1 | 1 | 1 | 1 | 4 |
| hsa-miR-330-5p | Gcom1 | 1 | 1 | 1 | 1 | 4 |
| hsa-miR-330-5p | HAPLN3 | 1 | 1 | 1 | 1 | 4 |
| hsa-miR-330-5p | ADRA1D | 1 | 1 | 1 | 1 | 4 |
| hsa-miR-330-5p | CDAN1 | 1 | 1 | 1 | 1 | 4 |
| hsa-miR-330-5p | CMTM4 | 1 | 1 | 1 | 1 | 4 |
| hsa-miR-330-5p | FBXL16 | 1 | 1 | 1 | 1 | 4 |
| hsa-miR-330-5p | GSG1L | 1 | 1 | 1 | 1 | 4 |
| hsa-miR-330-5p | CSPG4 | 1 | 1 | 1 | 1 | 4 |
| hsa-miR-330-5p | MGAT5B | 1 | 1 | 1 | 1 | 4 |
| hsa-miR-330-5p | C17orf50 | 1 | 1 | 1 | 1 | 4 |
| hsa-miR-330-5p | SLFN13 | 1 | 1 | 1 | 1 | 4 |
| hsa-miR-330-5p | CD300LG | 1 | 1 | 1 | 1 | 4 |
| hsa-miR-330-5p | KCTD11 | 1 | 1 | 1 | 1 | 4 |
| hsa-miR-330-5p | NOTUM | 1 | 1 | 1 | 1 | 4 |
| hsa-miR-330-5p | C18orf25 | 1 | 1 | 1 | 1 | 4 |
| hsa-miR-330-5p | CBLN2 | 1 | 1 | 1 | 1 | 4 |
| hsa-miR-330-5p | C19orf18 | 1 | 1 | 1 | 1 | 4 |
| hsa-miR-330-5p | SIX5 | 1 | 1 | 1 | 1 | 4 |
| hsa-miR-330-5p | FAM98C | 1 | 1 | 1 | 1 | 4 |
| hsa-miR-330-5p | CAPN12 | 1 | 1 | 1 | 1 | 4 |
| hsa-miR-330-5p | ZNF599 | 1 | 1 | 1 | 1 | 4 |
| hsa-miR-330-5p | CILP2 | 1 | 1 | 1 | 1 | 4 |
| hsa-miR-330-5p | C19orf25 | 1 | 1 | 1 | 1 | 4 |
| hsa-miR-330-5p | ATP8B3 | 1 | 1 | 1 | 1 | 4 |
| hsa-miR-330-5p | DIRAS1 | 1 | 1 | 1 | 1 | 4 |
| hsa-miR-330-5p | C1orf76 | 1 | 1 | 1 | 1 | 4 |
| hsa-miR-330-5p | SLC30A7 | 1 | 1 | 1 | 1 | 4 |
| hsa-miR-330-5p | KNCN | 1 | 1 | 1 | 1 | 4 |
| hsa-miR-330-5p | ZNF362 | 1 | 1 | 1 | 1 | 4 |
| hsa-miR-330-5p | CNIH3 | 1 | 1 | 1 | 1 | 4 |
| hsa-miR-330-5p | C1orf64 | 1 | 1 | 1 | 1 | 4 |
| hsa-miR-330-5p | C20orf186 | 1 | 1 | 1 | 1 | 4 |
| hsa-miR-330-5p | AANAT | 1 | 1 | 1 | 1 | 4 |
| hsa-miR-330-5p | CTNND1 | 1 | 1 | 1 | 1 | 4 |
| hsa-miR-330-5p | YDJC | 1 | 1 | 1 | 1 | 4 |
| hsa-miR-330-5p | CCDC117 | 1 | 1 | 1 | 1 | 4 |
| hsa-miR-330-5p | DUSP18 | 1 | 1 | 1 | 1 | 4 |
| hsa-miR-330-5p | CTPS | 1 | 1 | 1 | 1 | 4 |
| hsa-miR-330-5p | FAM109B | 1 | 1 | 1 | 1 | 4 |
| hsa-miR-330-5p | CKAP2L | 1 | 1 | 1 | 1 | 4 |
| hsa-miR-330-5p | SMYD1 | 1 | 1 | 1 | 1 | 4 |
| hsa-miR-330-5p | FBXO41 | 1 | 1 | 1 | 1 | 4 |
| hsa-miR-330-5p | CTSD | 1 | 1 | 1 | 1 | 4 |
| hsa-miR-330-5p | CTSE | 1 | 1 | 1 | 1 | 4 |
| hsa-miR-330-5p | ZNF385B | 1 | 1 | 1 | 1 | 4 |
| hsa-miR-330-5p | CTSS | 1 | 1 | 1 | 1 | 4 |
| hsa-miR-330-5p | C9orf19 | 1 | 1 | 1 | 1 | 4 |
| hsa-miR-330-5p | FGD5 | 1 | 1 | 1 | 1 | 4 |
| hsa-miR-330-5p | CX3CR1 | 1 | 1 | 1 | 1 | 4 |
| hsa-miR-330-5p | THAP6 | 1 | 1 | 1 | 1 | 4 |
| hsa-miR-330-5p | ADRB1 | 1 | 1 | 1 | 1 | 4 |
| hsa-miR-330-5p | RASGEF1B | 1 | 1 | 1 | 1 | 4 |
| hsa-miR-330-5p | DAB2IP | 1 | 1 | 1 | 1 | 4 |
| hsa-miR-330-5p | PLEKHG4B | 1 | 1 | 1 | 1 | 4 |
| hsa-miR-330-5p | CNKSR3 | 1 | 1 | 1 | 1 | 4 |
| hsa-miR-330-5p | SLC2A12 | 1 | 1 | 1 | 1 | 4 |
| hsa-miR-330-5p | CYP1A1 | 1 | 1 | 1 | 1 | 4 |
| hsa-miR-330-5p | RUNDC3B | 1 | 1 | 1 | 1 | 4 |
| hsa-miR-330-5p | CYP3A7 | 1 | 1 | 1 | 1 | 4 |
| hsa-miR-330-5p | RDH10 | 1 | 1 | 1 | 1 | 4 |
| hsa-miR-330-5p | CYP3A4 | 1 | 1 | 1 | 1 | 4 |
| hsa-miR-330-5p | CYP8B1 | 1 | 1 | 1 | 1 | 4 |
| hsa-miR-330-5p | RP13-102H20.1 | 1 | 1 | 1 | 1 | 4 |
| hsa-miR-330-5p | DAD1 | 1 | 1 | 1 | 1 | 4 |
| hsa-miR-330-5p | MGC24039 | 1 | 1 | 1 | 1 | 4 |
| hsa-miR-330-5p | GRASP | 1 | 1 | 1 | 1 | 4 |
| hsa-miR-330-5p | DAO | 1 | 1 | 1 | 1 | 4 |
| hsa-miR-330-5p | DAP | 1 | 1 | 1 | 1 | 4 |
| hsa-miR-330-5p | EXDL1 | 1 | 1 | 1 | 1 | 4 |
| hsa-miR-330-5p | RHBDL3 | 1 | 1 | 1 | 1 | 4 |
| hsa-miR-330-5p | TRPV3 | 1 | 1 | 1 | 1 | 4 |
| hsa-miR-330-5p | AP2B1 | 1 | 1 | 1 | 1 | 4 |
| hsa-miR-330-5p | ZNF781 | 1 | 1 | 1 | 1 | 4 |
| hsa-miR-330-5p | ZNF100 | 1 | 1 | 1 | 1 | 4 |
| hsa-miR-330-5p | ACE | 1 | 1 | 1 | 1 | 4 |
| hsa-miR-330-5p | IL28RA | 1 | 1 | 1 | 1 | 4 |
| hsa-miR-330-5p | CITED4 | 1 | 1 | 1 | 1 | 4 |
| hsa-miR-330-5p | C1orf177 | 1 | 1 | 1 | 1 | 4 |
| hsa-miR-330-5p | KANK4 | 1 | 1 | 1 | 1 | 4 |
| hsa-miR-330-5p | DDB1 | 1 | 1 | 1 | 1 | 4 |
| hsa-miR-330-5p | APCDD1L | 1 | 1 | 1 | 1 | 4 |
| hsa-miR-330-5p | DDB2 | 1 | 1 | 1 | 1 | 4 |
| hsa-miR-330-5p | TMPRSS6 | 1 | 1 | 1 | 1 | 4 |
| hsa-miR-330-5p | C2orf57 | 1 | 1 | 1 | 1 | 4 |
| hsa-miR-330-5p | DDX6 | 1 | 1 | 1 | 1 | 4 |
| hsa-miR-330-5p | PRICKLE2 | 1 | 1 | 1 | 1 | 4 |
| hsa-miR-330-5p | KLHDC6 | 1 | 1 | 1 | 1 | 4 |
| hsa-miR-330-5p | SPATA5 | 1 | 1 | 1 | 1 | 4 |
| hsa-miR-330-5p | DCLK2 | 1 | 1 | 1 | 1 | 4 |
| hsa-miR-330-5p | RASSF6 | 1 | 1 | 1 | 1 | 4 |
| hsa-miR-330-5p | CDC20B | 1 | 1 | 1 | 1 | 4 |
| hsa-miR-330-5p | DES | 1 | 1 | 1 | 1 | 4 |
| hsa-miR-330-5p | LIX1 | 1 | 1 | 1 | 1 | 4 |
| hsa-miR-330-5p | DFFA | 1 | 1 | 1 | 1 | 4 |
| hsa-miR-330-5p | SLC30A8 | 1 | 1 | 1 | 1 | 4 |
| hsa-miR-330-5p | COL22A1 | 1 | 1 | 1 | 1 | 4 |
| hsa-miR-330-5p | INDOL1 | 1 | 1 | 1 | 1 | 4 |
| hsa-miR-330-5p | OLFML2A | 1 | 1 | 1 | 1 | 4 |
| hsa-miR-330-5p | QSOX2 | 1 | 1 | 1 | 1 | 4 |
| hsa-miR-330-5p | GLIS3 | 1 | 1 | 1 | 1 | 4 |
| hsa-miR-330-5p | SPIN3 | 1 | 1 | 1 | 1 | 4 |
| hsa-miR-330-5p | PWWP2B | 1 | 1 | 1 | 1 | 4 |
| hsa-miR-330-5p | SPACA4 | 1 | 1 | 1 | 1 | 4 |
| hsa-miR-330-5p | DHCR24 | 1 | 1 | 1 | 1 | 4 |
| hsa-miR-330-5p | DLG3 | 1 | 1 | 1 | 1 | 4 |
| hsa-miR-330-5p | DNM1 | 1 | 1 | 1 | 1 | 4 |
| hsa-miR-330-5p | DMWD | 1 | 1 | 1 | 1 | 4 |
| hsa-miR-330-5p | DOCK2 | 1 | 1 | 1 | 1 | 4 |
| hsa-miR-330-5p | DOK1 | 1 | 1 | 1 | 1 | 4 |
| hsa-miR-330-5p | DPYSL2 | 1 | 1 | 1 | 1 | 4 |
| hsa-miR-330-5p | DRD2 | 1 | 1 | 1 | 1 | 4 |
| hsa-miR-330-5p | DRD5 | 1 | 1 | 1 | 1 | 4 |
| hsa-miR-330-5p | DSC3 | 1 | 1 | 1 | 1 | 4 |
| hsa-miR-330-5p | DSG2 | 1 | 1 | 1 | 1 | 4 |
| hsa-miR-330-5p | SLC26A2 | 1 | 1 | 1 | 1 | 4 |
| hsa-miR-330-5p | DTNB | 1 | 1 | 1 | 1 | 4 |
| hsa-miR-330-5p | DUSP9 | 1 | 1 | 1 | 1 | 4 |
| hsa-miR-330-5p | DVL3 | 1 | 1 | 1 | 1 | 4 |
| hsa-miR-330-5p | E2F2 | 1 | 1 | 1 | 1 | 4 |
| hsa-miR-330-5p | EBF1 | 1 | 1 | 1 | 1 | 4 |
| hsa-miR-330-5p | EDN2 | 1 | 1 | 1 | 1 | 4 |
| hsa-miR-330-5p | EDN3 | 1 | 1 | 1 | 1 | 4 |
| hsa-miR-330-5p | SCAMP5 | 1 | 1 | 1 | 1 | 4 |
| hsa-miR-330-5p | EFNA1 | 1 | 1 | 1 | 1 | 4 |
| hsa-miR-330-5p | EFNA5 | 1 | 1 | 1 | 1 | 4 |
| hsa-miR-330-5p | EFNB1 | 1 | 1 | 1 | 1 | 4 |
| hsa-miR-330-5p | EFNB3 | 1 | 1 | 1 | 1 | 4 |
| hsa-miR-330-5p | EGR3 | 1 | 1 | 1 | 1 | 4 |
| hsa-miR-330-5p | EGR4 | 1 | 1 | 1 | 1 | 4 |
| hsa-miR-330-5p | EIF2S1 | 1 | 1 | 1 | 1 | 4 |
| hsa-miR-330-5p | GRAMD2 | 1 | 1 | 1 | 1 | 4 |
| hsa-miR-330-5p | ELAVL1 | 1 | 1 | 1 | 1 | 4 |
| hsa-miR-330-5p | ELAVL3 | 1 | 1 | 1 | 1 | 4 |
| hsa-miR-330-5p | DAND5 | 1 | 1 | 1 | 1 | 4 |
| hsa-miR-330-5p | CADM4 | 1 | 1 | 1 | 1 | 4 |
| hsa-miR-330-5p | TMEM61 | 1 | 1 | 1 | 1 | 4 |
| hsa-miR-330-5p | ABCA2 | 1 | 1 | 1 | 1 | 4 |
| hsa-miR-330-5p | SLC5A9 | 1 | 1 | 1 | 1 | 4 |
| hsa-miR-330-5p | TXLNA | 1 | 1 | 1 | 1 | 4 |
| hsa-miR-330-5p | C1orf69 | 1 | 1 | 1 | 1 | 4 |
| hsa-miR-330-5p | ARL13B | 1 | 1 | 1 | 1 | 4 |
| hsa-miR-330-5p | DHFRL1 | 1 | 1 | 1 | 1 | 4 |
| hsa-miR-330-5p | EMD | 1 | 1 | 1 | 1 | 4 |
| hsa-miR-330-5p | LOC201164 | 1 | 1 | 1 | 1 | 4 |
| hsa-miR-330-5p | SAMD14 | 1 | 1 | 1 | 1 | 4 |
| hsa-miR-330-5p | EMP1 | 1 | 1 | 1 | 1 | 4 |
| hsa-miR-330-5p | STRA13 | 1 | 1 | 1 | 1 | 4 |
| hsa-miR-330-5p | TRIM65 | 1 | 1 | 1 | 1 | 4 |
| hsa-miR-330-5p | UNC13D | 1 | 1 | 1 | 1 | 4 |
| hsa-miR-330-5p | EMX1 | 1 | 1 | 1 | 1 | 4 |
| hsa-miR-330-5p | CTTN | 1 | 1 | 1 | 1 | 4 |
| hsa-miR-330-5p | LOC201725 | 1 | 1 | 1 | 1 | 4 |
| hsa-miR-330-5p | TMEM192 | 1 | 1 | 1 | 1 | 4 |
| hsa-miR-330-5p | ENG | 1 | 1 | 1 | 1 | 4 |
| hsa-miR-330-5p | TMEM184A | 1 | 1 | 1 | 1 | 4 |
| hsa-miR-330-5p | LGI3 | 1 | 1 | 1 | 1 | 4 |
| hsa-miR-330-5p | C9orf91 | 1 | 1 | 1 | 1 | 4 |
| hsa-miR-330-5p | C9orf25 | 1 | 1 | 1 | 1 | 4 |
| hsa-miR-330-5p | ANKS6 | 1 | 1 | 1 | 1 | 4 |
| hsa-miR-330-5p | EPAS1 | 1 | 1 | 1 | 1 | 4 |
| hsa-miR-330-5p | SLC25A43 | 1 | 1 | 1 | 1 | 4 |
| hsa-miR-330-5p | STOM | 1 | 1 | 1 | 1 | 4 |
| hsa-miR-330-5p | EPHA4 | 1 | 1 | 1 | 1 | 4 |
| hsa-miR-330-5p | EPHA8 | 1 | 1 | 1 | 1 | 4 |
| hsa-miR-330-5p | EPHB3 | 1 | 1 | 1 | 1 | 4 |
| hsa-miR-330-5p | STX2 | 1 | 1 | 1 | 1 | 4 |
| hsa-miR-330-5p | EPO | 1 | 1 | 1 | 1 | 4 |
| hsa-miR-330-5p | EPS15 | 1 | 1 | 1 | 1 | 4 |
| hsa-miR-330-5p | ERBB4 | 1 | 1 | 1 | 1 | 4 |
| hsa-miR-330-5p | ERN1 | 1 | 1 | 1 | 1 | 4 |
| hsa-miR-330-5p | ABCA3 | 1 | 1 | 1 | 1 | 4 |
| hsa-miR-330-5p | ESRRA | 1 | 1 | 1 | 1 | 4 |
| hsa-miR-330-5p | ETV6 | 1 | 1 | 1 | 1 | 4 |
| hsa-miR-330-5p | EVC | 1 | 1 | 1 | 1 | 4 |
| hsa-miR-330-5p | EXTL3 | 1 | 1 | 1 | 1 | 4 |
| hsa-miR-330-5p | EZH1 | 1 | 1 | 1 | 1 | 4 |
| hsa-miR-330-5p | ABCD1 | 1 | 1 | 1 | 1 | 4 |
| hsa-miR-330-5p | FABP2 | 1 | 1 | 1 | 1 | 4 |
| hsa-miR-330-5p | FANCE | 1 | 1 | 1 | 1 | 4 |
| hsa-miR-330-5p | ACSL1 | 1 | 1 | 1 | 1 | 4 |
| hsa-miR-330-5p | ALDH1B1 | 1 | 1 | 1 | 1 | 4 |
| hsa-miR-330-5p | UNC5B | 1 | 1 | 1 | 1 | 4 |
| hsa-miR-330-5p | TYSND1 | 1 | 1 | 1 | 1 | 4 |
| hsa-miR-330-5p | RTKN2 | 1 | 1 | 1 | 1 | 4 |
| hsa-miR-330-5p | C11orf45 | 1 | 1 | 1 | 1 | 4 |
| hsa-miR-330-5p | FBLN2 | 1 | 1 | 1 | 1 | 4 |
| hsa-miR-330-5p | ALDH1A3 | 1 | 1 | 1 | 1 | 4 |
| hsa-miR-330-5p | FBN1 | 1 | 1 | 1 | 1 | 4 |
| hsa-miR-330-5p | ORAOV1 | 1 | 1 | 1 | 1 | 4 |
| hsa-miR-330-5p | C18orf24 | 1 | 1 | 1 | 1 | 4 |
| hsa-miR-330-5p | HEPACAM | 1 | 1 | 1 | 1 | 4 |
| hsa-miR-330-5p | C10orf25 | 1 | 1 | 1 | 1 | 4 |
| hsa-miR-330-5p | ARL5B | 1 | 1 | 1 | 1 | 4 |
| hsa-miR-330-5p | C13orf3 | 1 | 1 | 1 | 1 | 4 |
| hsa-miR-330-5p | NT5DC1 | 1 | 1 | 1 | 1 | 4 |
| hsa-miR-330-5p | C6orf65 | 1 | 1 | 1 | 1 | 4 |
| hsa-miR-330-5p | KIF6 | 1 | 1 | 1 | 1 | 4 |
| hsa-miR-330-5p | C6orf89 | 1 | 1 | 1 | 1 | 4 |
| hsa-miR-330-5p | LEMD2 | 1 | 1 | 1 | 1 | 4 |
| hsa-miR-330-5p | FCGR3B | 1 | 1 | 1 | 1 | 4 |
| hsa-miR-330-5p | FOXK1 | 1 | 1 | 1 | 1 | 4 |
| hsa-miR-330-5p | MMD2 | 1 | 1 | 1 | 1 | 4 |
| hsa-miR-330-5p | C7orf41 | 1 | 1 | 1 | 1 | 4 |
| hsa-miR-330-5p | C10orf30 | 1 | 1 | 1 | 1 | 4 |
| hsa-miR-330-5p | SLC35F1 | 1 | 1 | 1 | 1 | 4 |
| hsa-miR-330-5p | KCTD20 | 1 | 1 | 1 | 1 | 4 |
| hsa-miR-330-5p | SCUBE3 | 1 | 1 | 1 | 1 | 4 |
| hsa-miR-330-5p | SLC29A4 | 1 | 1 | 1 | 1 | 4 |
| hsa-miR-330-5p | ALDH3A2 | 1 | 1 | 1 | 1 | 4 |
| hsa-miR-330-5p | FGF1 | 1 | 1 | 1 | 1 | 4 |
| hsa-miR-330-5p | FGF11 | 1 | 1 | 1 | 1 | 4 |
| hsa-miR-330-5p | FGFR1 | 1 | 1 | 1 | 1 | 4 |
| hsa-miR-330-5p | FGFR2 | 1 | 1 | 1 | 1 | 4 |
| hsa-miR-330-5p | ITGA11 | 1 | 1 | 1 | 1 | 4 |
| hsa-miR-330-5p | DLGAP4 | 1 | 1 | 1 | 1 | 4 |
| hsa-miR-330-5p | KIAA0831 | 1 | 1 | 1 | 1 | 4 |
| hsa-miR-330-5p | PLEKHA6 | 1 | 1 | 1 | 1 | 4 |
| hsa-miR-330-5p | MLXIP | 1 | 1 | 1 | 1 | 4 |
| hsa-miR-330-5p | MON1B | 1 | 1 | 1 | 1 | 4 |
| hsa-miR-330-5p | CLSTN1 | 1 | 1 | 1 | 1 | 4 |
| hsa-miR-330-5p | ABLIM3 | 1 | 1 | 1 | 1 | 4 |
| hsa-miR-330-5p | BTBD3 | 1 | 1 | 1 | 1 | 4 |
| hsa-miR-330-5p | EPN2 | 1 | 1 | 1 | 1 | 4 |
| hsa-miR-330-5p | TRAK1 | 1 | 1 | 1 | 1 | 4 |
| hsa-miR-330-5p | KLRK1 | 1 | 1 | 1 | 1 | 4 |
| hsa-miR-330-5p | CCT5 | 1 | 1 | 1 | 1 | 4 |
| hsa-miR-330-5p | SLC4A1AP | 1 | 1 | 1 | 1 | 4 |
| hsa-miR-330-5p | SCMH1 | 1 | 1 | 1 | 1 | 4 |
| hsa-miR-330-5p | DIP2C | 1 | 1 | 1 | 1 | 4 |
| hsa-miR-330-5p | PDCD11 | 1 | 1 | 1 | 1 | 4 |
| hsa-miR-330-5p | ACIN1 | 1 | 1 | 1 | 1 | 4 |
| hsa-miR-330-5p | MYH15 | 1 | 1 | 1 | 1 | 4 |
| hsa-miR-330-5p | AZI1 | 1 | 1 | 1 | 1 | 4 |
| hsa-miR-330-5p | KIAA0265 | 1 | 1 | 1 | 1 | 4 |
| hsa-miR-330-5p | RAB21 | 1 | 1 | 1 | 1 | 4 |
| hsa-miR-330-5p | FBXO21 | 1 | 1 | 1 | 1 | 4 |
| hsa-miR-330-5p | FAIM2 | 1 | 1 | 1 | 1 | 4 |
| hsa-miR-330-5p | FOXJ1 | 1 | 1 | 1 | 1 | 4 |
| hsa-miR-330-5p | ASCC3L1 | 1 | 1 | 1 | 1 | 4 |
| hsa-miR-330-5p | UNC13A | 1 | 1 | 1 | 1 | 4 |
| hsa-miR-330-5p | PHLPPL | 1 | 1 | 1 | 1 | 4 |
| hsa-miR-330-5p | FOXE1 | 1 | 1 | 1 | 1 | 4 |
| hsa-miR-330-5p | ZHX3 | 1 | 1 | 1 | 1 | 4 |
| hsa-miR-330-5p | ENDOD1 | 1 | 1 | 1 | 1 | 4 |
| hsa-miR-330-5p | ZNF609 | 1 | 1 | 1 | 1 | 4 |
| hsa-miR-330-5p | GGA2 | 1 | 1 | 1 | 1 | 4 |
| hsa-miR-330-5p | SETX | 1 | 1 | 1 | 1 | 4 |
| hsa-miR-330-5p | ARHGAP26 | 1 | 1 | 1 | 1 | 4 |
| hsa-miR-330-5p | SIPA1L3 | 1 | 1 | 1 | 1 | 4 |
| hsa-miR-330-5p | CDC2L6 | 1 | 1 | 1 | 1 | 4 |
| hsa-miR-330-5p | PARC | 1 | 1 | 1 | 1 | 4 |
| hsa-miR-330-5p | HIC2 | 1 | 1 | 1 | 1 | 4 |
| hsa-miR-330-5p | POGZ | 1 | 1 | 1 | 1 | 4 |
| hsa-miR-330-5p | PLXND1 | 1 | 1 | 1 | 1 | 4 |
| hsa-miR-330-5p | GPATCH8 | 1 | 1 | 1 | 1 | 4 |
| hsa-miR-330-5p | EPB41L3 | 1 | 1 | 1 | 1 | 4 |
| hsa-miR-330-5p | N4BP3 | 1 | 1 | 1 | 1 | 4 |
| hsa-miR-330-5p | GRAMD4 | 1 | 1 | 1 | 1 | 4 |
| hsa-miR-330-5p | CIC | 1 | 1 | 1 | 1 | 4 |
| hsa-miR-330-5p | CLCC1 | 1 | 1 | 1 | 1 | 4 |
| hsa-miR-330-5p | 6-Sep | 1 | 1 | 1 | 1 | 4 |
| hsa-miR-330-5p | MAPK8IP3 | 1 | 1 | 1 | 1 | 4 |
| hsa-miR-330-5p | GGA3 | 1 | 1 | 1 | 1 | 4 |
| hsa-miR-330-5p | METAP1 | 1 | 1 | 1 | 1 | 4 |
| hsa-miR-330-5p | ZCCHC14 | 1 | 1 | 1 | 1 | 4 |
| hsa-miR-330-5p | 7-Sep | 1 | 1 | 1 | 1 | 4 |
| hsa-miR-330-5p | RCOR1 | 1 | 1 | 1 | 1 | 4 |
| hsa-miR-330-5p | FLOT2 | 1 | 1 | 1 | 1 | 4 |
| hsa-miR-330-5p | GANAB | 1 | 1 | 1 | 1 | 4 |
| hsa-miR-330-5p | FBXL7 | 1 | 1 | 1 | 1 | 4 |
| hsa-miR-330-5p | UBXD8 | 1 | 1 | 1 | 1 | 4 |
| hsa-miR-330-5p | SYT11 | 1 | 1 | 1 | 1 | 4 |
| hsa-miR-330-5p | ZFR2 | 1 | 1 | 1 | 1 | 4 |
| hsa-miR-330-5p | DTX4 | 1 | 1 | 1 | 1 | 4 |
| hsa-miR-330-5p | RRP12 | 1 | 1 | 1 | 1 | 4 |
| hsa-miR-330-5p | SNF1LK2 | 1 | 1 | 1 | 1 | 4 |
| hsa-miR-330-5p | FLT4 | 1 | 1 | 1 | 1 | 4 |
| hsa-miR-330-5p | ASTN2 | 1 | 1 | 1 | 1 | 4 |
| hsa-miR-330-5p | KIAA0556 | 1 | 1 | 1 | 1 | 4 |
| hsa-miR-330-5p | ATP11A | 1 | 1 | 1 | 1 | 4 |
| hsa-miR-330-5p | RP1-21O18.1 | 1 | 1 | 1 | 1 | 4 |
| hsa-miR-330-5p | ZC3H7B | 1 | 1 | 1 | 1 | 4 |
| hsa-miR-330-5p | CLEC16A | 1 | 1 | 1 | 1 | 4 |
| hsa-miR-330-5p | POFUT2 | 1 | 1 | 1 | 1 | 4 |
| hsa-miR-330-5p | SMG6 | 1 | 1 | 1 | 1 | 4 |
| hsa-miR-330-5p | WSCD1 | 1 | 1 | 1 | 1 | 4 |
| hsa-miR-330-5p | ICOSLG | 1 | 1 | 1 | 1 | 4 |
| hsa-miR-330-5p | C22orf9 | 1 | 1 | 1 | 1 | 4 |
| hsa-miR-330-5p | USP22 | 1 | 1 | 1 | 1 | 4 |
| hsa-miR-330-5p | CLASP1 | 1 | 1 | 1 | 1 | 4 |
| hsa-miR-330-5p | WDR7 | 1 | 1 | 1 | 1 | 4 |
| hsa-miR-330-5p | VPS39 | 1 | 1 | 1 | 1 | 4 |
| hsa-miR-330-5p | KIAA0323 | 1 | 1 | 1 | 1 | 4 |
| hsa-miR-330-5p | ZNF629 | 1 | 1 | 1 | 1 | 4 |
| hsa-miR-330-5p | PSD3 | 1 | 1 | 1 | 1 | 4 |
| hsa-miR-330-5p | ARHGEF12 | 1 | 1 | 1 | 1 | 4 |
| hsa-miR-330-5p | SRGAP2 | 1 | 1 | 1 | 1 | 4 |
| hsa-miR-330-5p | AHCYL2 | 1 | 1 | 1 | 1 | 4 |
| hsa-miR-330-5p | KIAA0892 | 1 | 1 | 1 | 1 | 4 |
| hsa-miR-330-5p | NUDCD3 | 1 | 1 | 1 | 1 | 4 |
| hsa-miR-330-5p | MED13L | 1 | 1 | 1 | 1 | 4 |
| hsa-miR-330-5p | LARS2 | 1 | 1 | 1 | 1 | 4 |
| hsa-miR-330-5p | PIP5K1C | 1 | 1 | 1 | 1 | 4 |
| hsa-miR-330-5p | FRAT2 | 1 | 1 | 1 | 1 | 4 |
| hsa-miR-330-5p | COTL1 | 1 | 1 | 1 | 1 | 4 |
| hsa-miR-330-5p | SIRT1 | 1 | 1 | 1 | 1 | 4 |
| hsa-miR-330-5p | FREQ | 1 | 1 | 1 | 1 | 4 |
| hsa-miR-330-5p | CRB1 | 1 | 1 | 1 | 1 | 4 |
| hsa-miR-330-5p | GPR161 | 1 | 1 | 1 | 1 | 4 |
| hsa-miR-330-5p | SLC35A3 | 1 | 1 | 1 | 1 | 4 |
| hsa-miR-330-5p | SF3B3 | 1 | 1 | 1 | 1 | 4 |
| hsa-miR-330-5p | ICMT | 1 | 1 | 1 | 1 | 4 |
| hsa-miR-330-5p | GCAT | 1 | 1 | 1 | 1 | 4 |
| hsa-miR-330-5p | CBX6 | 1 | 1 | 1 | 1 | 4 |
| hsa-miR-330-5p | CES3 | 1 | 1 | 1 | 1 | 4 |
| hsa-miR-330-5p | CBX7 | 1 | 1 | 1 | 1 | 4 |
| hsa-miR-330-5p | HEY2 | 1 | 1 | 1 | 1 | 4 |
| hsa-miR-330-5p | POFUT1 | 1 | 1 | 1 | 1 | 4 |
| hsa-miR-330-5p | CENTB2 | 1 | 1 | 1 | 1 | 4 |
| hsa-miR-330-5p | FOSB | 1 | 1 | 1 | 1 | 4 |
| hsa-miR-330-5p | SEZ6L | 1 | 1 | 1 | 1 | 4 |
| hsa-miR-330-5p | WBP2 | 1 | 1 | 1 | 1 | 4 |
| hsa-miR-330-5p | C17orf81 | 1 | 1 | 1 | 1 | 4 |
| hsa-miR-330-5p | CORO1C | 1 | 1 | 1 | 1 | 4 |
| hsa-miR-330-5p | DAPK2 | 1 | 1 | 1 | 1 | 4 |
| hsa-miR-330-5p | C9orf5 | 1 | 1 | 1 | 1 | 4 |
| hsa-miR-330-5p | C15orf2 | 1 | 1 | 1 | 1 | 4 |
| hsa-miR-330-5p | BHMT2 | 1 | 1 | 1 | 1 | 4 |
| hsa-miR-330-5p | AIPL1 | 1 | 1 | 1 | 1 | 4 |
| hsa-miR-330-5p | OSBP2 | 1 | 1 | 1 | 1 | 4 |
| hsa-miR-330-5p | FKBP8 | 1 | 1 | 1 | 1 | 4 |
| hsa-miR-330-5p | BCL2L13 | 1 | 1 | 1 | 1 | 4 |
| hsa-miR-330-5p | CNPY4 | 1 | 1 | 1 | 1 | 4 |
| hsa-miR-330-5p | ATP6V1C2 | 1 | 1 | 1 | 1 | 4 |
| hsa-miR-330-5p | CNOT6L | 1 | 1 | 1 | 1 | 4 |
| hsa-miR-330-5p | STAC3 | 1 | 1 | 1 | 1 | 4 |
| hsa-miR-330-5p | PELI3 | 1 | 1 | 1 | 1 | 4 |
| hsa-miR-330-5p | ALPL | 1 | 1 | 1 | 1 | 4 |
| hsa-miR-330-5p | NR5A2 | 1 | 1 | 1 | 1 | 4 |
| hsa-miR-330-5p | NR5A1 | 1 | 1 | 1 | 1 | 4 |
| hsa-miR-330-5p | FUT2 | 1 | 1 | 1 | 1 | 4 |
| hsa-miR-330-5p | STXBP4 | 1 | 1 | 1 | 1 | 4 |
| hsa-miR-330-5p | FNDC5 | 1 | 1 | 1 | 1 | 4 |
| hsa-miR-330-5p | RICTOR | 1 | 1 | 1 | 1 | 4 |
| hsa-miR-330-5p | LASS6 | 1 | 1 | 1 | 1 | 4 |
| hsa-miR-330-5p | G6PD | 1 | 1 | 1 | 1 | 4 |
| hsa-miR-330-5p | RNF169 | 1 | 1 | 1 | 1 | 4 |
| hsa-miR-330-5p | SLC41A1 | 1 | 1 | 1 | 1 | 4 |
| hsa-miR-330-5p | C8orf46 | 1 | 1 | 1 | 1 | 4 |
| hsa-miR-330-5p | GAB1 | 1 | 1 | 1 | 1 | 4 |
| hsa-miR-330-5p | TMCO4 | 1 | 1 | 1 | 1 | 4 |
| hsa-miR-330-5p | GABRA4 | 1 | 1 | 1 | 1 | 4 |
| hsa-miR-330-5p | ZNF549 | 1 | 1 | 1 | 1 | 4 |
| hsa-miR-330-5p | GABRB3 | 1 | 1 | 1 | 1 | 4 |
| hsa-miR-330-5p | GK5 | 1 | 1 | 1 | 1 | 4 |
| hsa-miR-330-5p | SCML4 | 1 | 1 | 1 | 1 | 4 |
| hsa-miR-330-5p | TCERG1L | 1 | 1 | 1 | 1 | 4 |
| hsa-miR-330-5p | GABRP | 1 | 1 | 1 | 1 | 4 |
| hsa-miR-330-5p | MAP3K7IP3 | 1 | 1 | 1 | 1 | 4 |
| hsa-miR-330-5p | ANKS4B | 1 | 1 | 1 | 1 | 4 |
| hsa-miR-330-5p | RIPK5 | 1 | 1 | 1 | 1 | 4 |
| hsa-miR-330-5p | RAB3GAP2 | 1 | 1 | 1 | 1 | 4 |
| hsa-miR-330-5p | TMEM59L | 1 | 1 | 1 | 1 | 4 |
| hsa-miR-330-5p | NGEF | 1 | 1 | 1 | 1 | 4 |
| hsa-miR-330-5p | ZNF324 | 1 | 1 | 1 | 1 | 4 |
| hsa-miR-330-5p | ATXN10 | 1 | 1 | 1 | 1 | 4 |
| hsa-miR-330-5p | TMEM184B | 1 | 1 | 1 | 1 | 4 |
| hsa-miR-330-5p | B4GALNT1 | 1 | 1 | 1 | 1 | 4 |
| hsa-miR-330-5p | SULT4A1 | 1 | 1 | 1 | 1 | 4 |
| hsa-miR-330-5p | DKFZP564O0823 | 1 | 1 | 1 | 1 | 4 |
| hsa-miR-330-5p | DKFZP434B0335 | 1 | 1 | 1 | 1 | 4 |
| hsa-miR-330-5p | DFNB31 | 1 | 1 | 1 | 1 | 4 |
| hsa-miR-330-5p | C3orf17 | 1 | 1 | 1 | 1 | 4 |
| hsa-miR-330-5p | GALNS | 1 | 1 | 1 | 1 | 4 |
| hsa-miR-330-5p | POLR1A | 1 | 1 | 1 | 1 | 4 |
| hsa-miR-330-5p | TRIM58 | 1 | 1 | 1 | 1 | 4 |
| hsa-miR-330-5p | FAM119B | 1 | 1 | 1 | 1 | 4 |
| hsa-miR-330-5p | ALS2CL | 1 | 1 | 1 | 1 | 4 |
| hsa-miR-330-5p | SGMS1 | 1 | 1 | 1 | 1 | 4 |
| hsa-miR-330-5p | TMIE | 1 | 1 | 1 | 1 | 4 |
| hsa-miR-330-5p | MYRIP | 1 | 1 | 1 | 1 | 4 |
| hsa-miR-330-5p | C2CD2 | 1 | 1 | 1 | 1 | 4 |
| hsa-miR-330-5p | PARS2 | 1 | 1 | 1 | 1 | 4 |
| hsa-miR-330-5p | C20orf4 | 1 | 1 | 1 | 1 | 4 |
| hsa-miR-330-5p | ULK3 | 1 | 1 | 1 | 1 | 4 |
| hsa-miR-330-5p | DAK | 1 | 1 | 1 | 1 | 4 |
| hsa-miR-330-5p | OSBPL3 | 1 | 1 | 1 | 1 | 4 |
| hsa-miR-330-5p | SUSD5 | 1 | 1 | 1 | 1 | 4 |
| hsa-miR-330-5p | GLCE | 1 | 1 | 1 | 1 | 4 |
| hsa-miR-330-5p | ZNF451 | 1 | 1 | 1 | 1 | 4 |
| hsa-miR-330-5p | CHD5 | 1 | 1 | 1 | 1 | 4 |
| hsa-miR-330-5p | CNTNAP2 | 1 | 1 | 1 | 1 | 4 |
| hsa-miR-330-5p | PPP1R16B | 1 | 1 | 1 | 1 | 4 |
| hsa-miR-330-5p | FAM127B | 1 | 1 | 1 | 1 | 4 |
| hsa-miR-330-5p | SGEF | 1 | 1 | 1 | 1 | 4 |
| hsa-miR-330-5p | TOR1AIP1 | 1 | 1 | 1 | 1 | 4 |
| hsa-miR-330-5p | CCDC9 | 1 | 1 | 1 | 1 | 4 |
| hsa-miR-330-5p | CCDC69 | 1 | 1 | 1 | 1 | 4 |
| hsa-miR-330-5p | TCTN3 | 1 | 1 | 1 | 1 | 4 |
| hsa-miR-330-5p | TRPC4AP | 1 | 1 | 1 | 1 | 4 |
| hsa-miR-330-5p | SERBP1 | 1 | 1 | 1 | 1 | 4 |
| hsa-miR-330-5p | TES | 1 | 1 | 1 | 1 | 4 |
| hsa-miR-330-5p | PHF19 | 1 | 1 | 1 | 1 | 4 |
| hsa-miR-330-5p | LRRC32 | 1 | 1 | 1 | 1 | 4 |
| hsa-miR-330-5p | AMD1 | 1 | 1 | 1 | 1 | 4 |
| hsa-miR-330-5p | GMEB2 | 1 | 1 | 1 | 1 | 4 |
| hsa-miR-330-5p | PITPNC1 | 1 | 1 | 1 | 1 | 4 |
| hsa-miR-330-5p | GAS8 | 1 | 1 | 1 | 1 | 4 |
| hsa-miR-330-5p | NKX2-8 | 1 | 1 | 1 | 1 | 4 |
| hsa-miR-330-5p | GATA4 | 1 | 1 | 1 | 1 | 4 |
| hsa-miR-330-5p | TSPAN17 | 1 | 1 | 1 | 1 | 4 |
| hsa-miR-330-5p | ERAL1 | 1 | 1 | 1 | 1 | 4 |
| hsa-miR-330-5p | PTPN18 | 1 | 1 | 1 | 1 | 4 |
| hsa-miR-330-5p | SLC17A5 | 1 | 1 | 1 | 1 | 4 |
| hsa-miR-330-5p | CNNM4 | 1 | 1 | 1 | 1 | 4 |
| hsa-miR-330-5p | HEYL | 1 | 1 | 1 | 1 | 4 |
| hsa-miR-330-5p | FER1L3 | 1 | 1 | 1 | 1 | 4 |
| hsa-miR-330-5p | EIF2C1 | 1 | 1 | 1 | 1 | 4 |
| hsa-miR-330-5p | IL1F5 | 1 | 1 | 1 | 1 | 4 |
| hsa-miR-330-5p | RGS17 | 1 | 1 | 1 | 1 | 4 |
| hsa-miR-330-5p | GREM1 | 1 | 1 | 1 | 1 | 4 |
| hsa-miR-330-5p | GDF10 | 1 | 1 | 1 | 1 | 4 |
| hsa-miR-330-5p | MDGA1 | 1 | 1 | 1 | 1 | 4 |
| hsa-miR-330-5p | GFAP | 1 | 1 | 1 | 1 | 4 |
| hsa-miR-330-5p | GFER | 1 | 1 | 1 | 1 | 4 |
| hsa-miR-330-5p | GGT5 | 1 | 1 | 1 | 1 | 4 |
| hsa-miR-330-5p | GJA3 | 1 | 1 | 1 | 1 | 4 |
| hsa-miR-330-5p | GJA4 | 1 | 1 | 1 | 1 | 4 |
| hsa-miR-330-5p | MLH3 | 1 | 1 | 1 | 1 | 4 |
| hsa-miR-330-5p | SND1 | 1 | 1 | 1 | 1 | 4 |
| hsa-miR-330-5p | GHITM | 1 | 1 | 1 | 1 | 4 |
| hsa-miR-330-5p | CACNG4 | 1 | 1 | 1 | 1 | 4 |
| hsa-miR-330-5p | AMPD2 | 1 | 1 | 1 | 1 | 4 |
| hsa-miR-330-5p | TMEM28 | 1 | 1 | 1 | 1 | 4 |
| hsa-miR-330-5p | BBC3 | 1 | 1 | 1 | 1 | 4 |
| hsa-miR-330-5p | PDE7B | 1 | 1 | 1 | 1 | 4 |
| hsa-miR-330-5p | BRPF3 | 1 | 1 | 1 | 1 | 4 |
| hsa-miR-330-5p | EIF2C2 | 1 | 1 | 1 | 1 | 4 |
| hsa-miR-330-5p | IL17C | 1 | 1 | 1 | 1 | 4 |
| hsa-miR-330-5p | ARHGEF16 | 1 | 1 | 1 | 1 | 4 |
| hsa-miR-330-5p | VENTX | 1 | 1 | 1 | 1 | 4 |
| hsa-miR-330-5p | SMPDL3B | 1 | 1 | 1 | 1 | 4 |
| hsa-miR-330-5p | GLE1 | 1 | 1 | 1 | 1 | 4 |
| hsa-miR-330-5p | GLI3 | 1 | 1 | 1 | 1 | 4 |
| hsa-miR-330-5p | GLP1R | 1 | 1 | 1 | 1 | 4 |
| hsa-miR-330-5p | GLS | 1 | 1 | 1 | 1 | 4 |
| hsa-miR-330-5p | GLUD1 | 1 | 1 | 1 | 1 | 4 |
| hsa-miR-330-5p | GLUD2 | 1 | 1 | 1 | 1 | 4 |
| hsa-miR-330-5p | GMFB | 1 | 1 | 1 | 1 | 4 |
| hsa-miR-330-5p | GML | 1 | 1 | 1 | 1 | 4 |
| hsa-miR-330-5p | GNA12 | 1 | 1 | 1 | 1 | 4 |
| hsa-miR-330-5p | GNAT1 | 1 | 1 | 1 | 1 | 4 |
| hsa-miR-330-5p | GNB1 | 1 | 1 | 1 | 1 | 4 |
| hsa-miR-330-5p | GNB3 | 1 | 1 | 1 | 1 | 4 |
| hsa-miR-330-5p | GNG3 | 1 | 1 | 1 | 1 | 4 |
| hsa-miR-330-5p | GNGT2 | 1 | 1 | 1 | 1 | 4 |
| hsa-miR-330-5p | GNS | 1 | 1 | 1 | 1 | 4 |
| hsa-miR-330-5p | GOT1 | 1 | 1 | 1 | 1 | 4 |
| hsa-miR-330-5p | GPC1 | 1 | 1 | 1 | 1 | 4 |
| hsa-miR-330-5p | GPD1 | 1 | 1 | 1 | 1 | 4 |
| hsa-miR-330-5p | GPI | 1 | 1 | 1 | 1 | 4 |
| hsa-miR-330-5p | SLCO4A1 | 1 | 1 | 1 | 1 | 4 |
| hsa-miR-330-5p | P4HA3 | 1 | 1 | 1 | 1 | 4 |
| hsa-miR-330-5p | C17orf28 | 1 | 1 | 1 | 1 | 4 |
| hsa-miR-330-5p | GPR17 | 1 | 1 | 1 | 1 | 4 |
| hsa-miR-330-5p | SLC13A5 | 1 | 1 | 1 | 1 | 4 |
| hsa-miR-330-5p | GPR19 | 1 | 1 | 1 | 1 | 4 |
| hsa-miR-330-5p | GPR20 | 1 | 1 | 1 | 1 | 4 |
| hsa-miR-330-5p | ZSCAN1 | 1 | 1 | 1 | 1 | 4 |
| hsa-miR-330-5p | KLK9 | 1 | 1 | 1 | 1 | 4 |
| hsa-miR-330-5p | SLC25A42 | 1 | 1 | 1 | 1 | 4 |
| hsa-miR-330-5p | ZNF493 | 1 | 1 | 1 | 1 | 4 |
| hsa-miR-330-5p | SYPL2 | 1 | 1 | 1 | 1 | 4 |
| hsa-miR-330-5p | SLC25A34 | 1 | 1 | 1 | 1 | 4 |
| hsa-miR-330-5p | GPR26 | 1 | 1 | 1 | 1 | 4 |
| hsa-miR-330-5p | SEC14L4 | 1 | 1 | 1 | 1 | 4 |
| hsa-miR-330-5p | RABL3 | 1 | 1 | 1 | 1 | 4 |
| hsa-miR-330-5p | FRYL | 1 | 1 | 1 | 1 | 4 |
| hsa-miR-330-5p | LOC285636 | 1 | 1 | 1 | 1 | 4 |
| hsa-miR-330-5p | SFRS12IP1 | 1 | 1 | 1 | 1 | 4 |
| hsa-miR-330-5p | ANK1 | 1 | 1 | 1 | 1 | 4 |
| hsa-miR-330-5p | ZFP41 | 1 | 1 | 1 | 1 | 4 |
| hsa-miR-330-5p | GRK6 | 1 | 1 | 1 | 1 | 4 |
| hsa-miR-330-5p | GRIA2 | 1 | 1 | 1 | 1 | 4 |
| hsa-miR-330-5p | GRID1 | 1 | 1 | 1 | 1 | 4 |
| hsa-miR-330-5p | OSTM1 | 1 | 1 | 1 | 1 | 4 |
| hsa-miR-330-5p | NOB1 | 1 | 1 | 1 | 1 | 4 |
| hsa-miR-330-5p | GRIK3 | 1 | 1 | 1 | 1 | 4 |
| hsa-miR-330-5p | ABR | 1 | 1 | 1 | 1 | 4 |
| hsa-miR-330-5p | C16orf72 | 1 | 1 | 1 | 1 | 4 |
| hsa-miR-330-5p | WDR91 | 1 | 1 | 1 | 1 | 4 |
| hsa-miR-330-5p | SCG3 | 1 | 1 | 1 | 1 | 4 |
| hsa-miR-330-5p | FHOD1 | 1 | 1 | 1 | 1 | 4 |
| hsa-miR-330-5p | SAP30BP | 1 | 1 | 1 | 1 | 4 |
| hsa-miR-330-5p | GSTT1 | 1 | 1 | 1 | 1 | 4 |
| hsa-miR-330-5p | GTF2A2 | 1 | 1 | 1 | 1 | 4 |
| hsa-miR-330-5p | GTF3C1 | 1 | 1 | 1 | 1 | 4 |
| hsa-miR-330-5p | CYP2S1 | 1 | 1 | 1 | 1 | 4 |
| hsa-miR-330-5p | UCRC | 1 | 1 | 1 | 1 | 4 |
| hsa-miR-330-5p | YPEL1 | 1 | 1 | 1 | 1 | 4 |
| hsa-miR-330-5p | TFCP2L1 | 1 | 1 | 1 | 1 | 4 |
| hsa-miR-330-5p | SENP1 | 1 | 1 | 1 | 1 | 4 |
| hsa-miR-330-5p | ICOS | 1 | 1 | 1 | 1 | 4 |
| hsa-miR-330-5p | NPC1L1 | 1 | 1 | 1 | 1 | 4 |
| hsa-miR-330-5p | EEF2K | 1 | 1 | 1 | 1 | 4 |
| hsa-miR-330-5p | SEC61A1 | 1 | 1 | 1 | 1 | 4 |
| hsa-miR-330-5p | GPR132 | 1 | 1 | 1 | 1 | 4 |
| hsa-miR-330-5p | PADI1 | 1 | 1 | 1 | 1 | 4 |
| hsa-miR-330-5p | GYPC | 1 | 1 | 1 | 1 | 4 |
| hsa-miR-330-5p | TRHDE | 1 | 1 | 1 | 1 | 4 |
| hsa-miR-330-5p | SLC2A8 | 1 | 1 | 1 | 1 | 4 |
| hsa-miR-330-5p | PACSIN1 | 1 | 1 | 1 | 1 | 4 |
| hsa-miR-330-5p | EFEMP2 | 1 | 1 | 1 | 1 | 4 |
| hsa-miR-330-5p | HTT | 1 | 1 | 1 | 1 | 4 |
| hsa-miR-330-5p | HUNK | 1 | 1 | 1 | 1 | 4 |
| hsa-miR-330-5p | ST6GALNAC6 | 1 | 1 | 1 | 1 | 4 |
| hsa-miR-330-5p | KCNIP3 | 1 | 1 | 1 | 1 | 4 |
| hsa-miR-330-5p | CD209 | 1 | 1 | 1 | 1 | 4 |
| hsa-miR-330-5p | EHD3 | 1 | 1 | 1 | 1 | 4 |
| hsa-miR-330-5p | CDR2L | 1 | 1 | 1 | 1 | 4 |
| hsa-miR-330-5p | TAX1BP3 | 1 | 1 | 1 | 1 | 4 |
| hsa-miR-330-5p | ANXA6 | 1 | 1 | 1 | 1 | 4 |
| hsa-miR-330-5p | HIP1 | 1 | 1 | 1 | 1 | 4 |
| hsa-miR-330-5p | HLA-A | 1 | 1 | 1 | 1 | 4 |
| hsa-miR-330-5p | HLA-C | 1 | 1 | 1 | 1 | 4 |
| hsa-miR-330-5p | HLF | 1 | 1 | 1 | 1 | 4 |
| hsa-miR-330-5p | HLX | 1 | 1 | 1 | 1 | 4 |
| hsa-miR-330-5p | HMGB3 | 1 | 1 | 1 | 1 | 4 |
| hsa-miR-330-5p | HMGCR | 1 | 1 | 1 | 1 | 4 |
| hsa-miR-330-5p | HNF4A | 1 | 1 | 1 | 1 | 4 |
| hsa-miR-330-5p | HNRNPA1 | 1 | 1 | 1 | 1 | 4 |
| hsa-miR-330-5p | HNRNPA2B1 | 1 | 1 | 1 | 1 | 4 |
| hsa-miR-330-5p | TLX1 | 1 | 1 | 1 | 1 | 4 |
| hsa-miR-330-5p | HOXA1 | 1 | 1 | 1 | 1 | 4 |
| hsa-miR-330-5p | HOXA7 | 1 | 1 | 1 | 1 | 4 |
| hsa-miR-330-5p | HPCA | 1 | 1 | 1 | 1 | 4 |
| hsa-miR-330-5p | APBA2 | 1 | 1 | 1 | 1 | 4 |
| hsa-miR-330-5p | HOXB3 | 1 | 1 | 1 | 1 | 4 |
| hsa-miR-330-5p | HOXC10 | 1 | 1 | 1 | 1 | 4 |
| hsa-miR-330-5p | HOXC11 | 1 | 1 | 1 | 1 | 4 |
| hsa-miR-330-5p | HPS1 | 1 | 1 | 1 | 1 | 4 |
| hsa-miR-330-5p | MMAB | 1 | 1 | 1 | 1 | 4 |
| hsa-miR-330-5p | HRBL | 1 | 1 | 1 | 1 | 4 |
| hsa-miR-330-5p | HSD11B1 | 1 | 1 | 1 | 1 | 4 |
| hsa-miR-330-5p | DNAJB2 | 1 | 1 | 1 | 1 | 4 |
| hsa-miR-330-5p | XIAP | 1 | 1 | 1 | 1 | 4 |
| hsa-miR-330-5p | SNAI3 | 1 | 1 | 1 | 1 | 4 |
| hsa-miR-330-5p | HTR4 | 1 | 1 | 1 | 1 | 4 |
| hsa-miR-330-5p | HTR7 | 1 | 1 | 1 | 1 | 4 |
| hsa-miR-330-5p | ICAM1 | 1 | 1 | 1 | 1 | 4 |
| hsa-miR-330-5p | NLRP14 | 1 | 1 | 1 | 1 | 4 |
| hsa-miR-330-5p | S100A7A | 1 | 1 | 1 | 1 | 4 |
| hsa-miR-330-5p | GPIHBP1 | 1 | 1 | 1 | 1 | 4 |
| hsa-miR-330-5p | ANKRD45 | 1 | 1 | 1 | 1 | 4 |
| hsa-miR-330-5p | TFAP2E | 1 | 1 | 1 | 1 | 4 |
| hsa-miR-330-5p | ID1 | 1 | 1 | 1 | 1 | 4 |
| hsa-miR-330-5p | ZSCAN22 | 1 | 1 | 1 | 1 | 4 |
| hsa-miR-330-5p | AQP8 | 1 | 1 | 1 | 1 | 4 |
| hsa-miR-330-5p | RSPO4 | 1 | 1 | 1 | 1 | 4 |
| hsa-miR-330-5p | IFNAR1 | 1 | 1 | 1 | 1 | 4 |
| hsa-miR-330-5p | IFNGR2 | 1 | 1 | 1 | 1 | 4 |
| hsa-miR-330-5p | ZNF81 | 1 | 1 | 1 | 1 | 4 |
| hsa-miR-330-5p | IGFBP5 | 1 | 1 | 1 | 1 | 4 |
| hsa-miR-330-5p | ICHTHYIN | 1 | 1 | 1 | 1 | 4 |
| hsa-miR-330-5p | ACADS | 1 | 1 | 1 | 1 | 4 |
| hsa-miR-330-5p | KAAG1 | 1 | 1 | 1 | 1 | 4 |
| hsa-miR-330-5p | ZNF445 | 1 | 1 | 1 | 1 | 4 |
| hsa-miR-330-5p | BMP8A | 1 | 1 | 1 | 1 | 4 |
| hsa-miR-330-5p | IHH | 1 | 1 | 1 | 1 | 4 |
| hsa-miR-330-5p | IL1R1 | 1 | 1 | 1 | 1 | 4 |
| hsa-miR-330-5p | AQP2 | 1 | 1 | 1 | 1 | 4 |
| hsa-miR-330-5p | FAM101B | 1 | 1 | 1 | 1 | 4 |
| hsa-miR-330-5p | AQP3 | 1 | 1 | 1 | 1 | 4 |
| hsa-miR-330-5p | ILF3 | 1 | 1 | 1 | 1 | 4 |
| hsa-miR-330-5p | AQP4 | 1 | 1 | 1 | 1 | 4 |
| hsa-miR-330-5p | IMPA2 | 1 | 1 | 1 | 1 | 4 |
| hsa-miR-330-5p | INHBA | 1 | 1 | 1 | 1 | 4 |
| hsa-miR-330-5p | INHBC | 1 | 1 | 1 | 1 | 4 |
| hsa-miR-330-5p | ITGA3 | 1 | 1 | 1 | 1 | 4 |
| hsa-miR-330-5p | ITGAX | 1 | 1 | 1 | 1 | 4 |
| hsa-miR-330-5p | ITGB8 | 1 | 1 | 1 | 1 | 4 |
| hsa-miR-330-5p | ITPKA | 1 | 1 | 1 | 1 | 4 |
| hsa-miR-330-5p | ITPKB | 1 | 1 | 1 | 1 | 4 |
| hsa-miR-330-5p | ITPR2 | 1 | 1 | 1 | 1 | 4 |
| hsa-miR-330-5p | ARCN1 | 1 | 1 | 1 | 1 | 4 |
| hsa-miR-330-5p | KCNA1 | 1 | 1 | 1 | 1 | 4 |
| hsa-miR-330-5p | TBC1D10C | 1 | 1 | 1 | 1 | 4 |
| hsa-miR-330-5p | KCNB1 | 1 | 1 | 1 | 1 | 4 |
| hsa-miR-330-5p | KCNC4 | 1 | 1 | 1 | 1 | 4 |
| hsa-miR-330-5p | PFN4 | 1 | 1 | 1 | 1 | 4 |
| hsa-miR-330-5p | LHFPL4 | 1 | 1 | 1 | 1 | 4 |
| hsa-miR-330-5p | C7orf52 | 1 | 1 | 1 | 1 | 4 |
| hsa-miR-330-5p | C9orf165 | 1 | 1 | 1 | 1 | 4 |
| hsa-miR-330-5p | AGRN | 1 | 1 | 1 | 1 | 4 |
| hsa-miR-330-5p | SLC27A1 | 1 | 1 | 1 | 1 | 4 |
| hsa-miR-330-5p | KCNJ10 | 1 | 1 | 1 | 1 | 4 |
| hsa-miR-330-5p | KCNJ12 | 1 | 1 | 1 | 1 | 4 |
| hsa-miR-330-5p | CA13 | 1 | 1 | 1 | 1 | 4 |
| hsa-miR-330-5p | KCNK3 | 1 | 1 | 1 | 1 | 4 |
| hsa-miR-330-5p | ARF4 | 1 | 1 | 1 | 1 | 4 |
| hsa-miR-330-5p | KCNQ1 | 1 | 1 | 1 | 1 | 4 |
| hsa-miR-330-5p | ARL4D | 1 | 1 | 1 | 1 | 4 |
| hsa-miR-330-5p | KLK2 | 1 | 1 | 1 | 1 | 4 |
| hsa-miR-330-5p | KPNA1 | 1 | 1 | 1 | 1 | 4 |
| hsa-miR-330-5p | KRT13 | 1 | 1 | 1 | 1 | 4 |
| hsa-miR-330-5p | C6orf120 | 1 | 1 | 1 | 1 | 4 |
| hsa-miR-330-5p | C10orf99 | 1 | 1 | 1 | 1 | 4 |
| hsa-miR-330-5p | KRT31 | 1 | 1 | 1 | 1 | 4 |
| hsa-miR-330-5p | RNF207 | 1 | 1 | 1 | 1 | 4 |
| hsa-miR-330-5p | SLC6A17 | 1 | 1 | 1 | 1 | 4 |
| hsa-miR-330-5p | LOC388969 | 1 | 1 | 1 | 1 | 4 |
| hsa-miR-330-5p | KRT84 | 1 | 1 | 1 | 1 | 4 |
| hsa-miR-330-5p | VGLL3 | 1 | 1 | 1 | 1 | 4 |
| hsa-miR-330-5p | CCDC4 | 1 | 1 | 1 | 1 | 4 |
| hsa-miR-330-5p | FLJ41603 | 1 | 1 | 1 | 1 | 4 |
| hsa-miR-330-5p | LIN28B | 1 | 1 | 1 | 1 | 4 |
| hsa-miR-330-5p | IYD | 1 | 1 | 1 | 1 | 4 |
| hsa-miR-330-5p | L1CAM | 1 | 1 | 1 | 1 | 4 |
| hsa-miR-330-5p | LAD1 | 1 | 1 | 1 | 1 | 4 |
| hsa-miR-330-5p | LAIR1 | 1 | 1 | 1 | 1 | 4 |
| hsa-miR-330-5p | LAMP1 | 1 | 1 | 1 | 1 | 4 |
| hsa-miR-330-5p | C9orf128 | 1 | 1 | 1 | 1 | 4 |
| hsa-miR-330-5p | LASP1 | 1 | 1 | 1 | 1 | 4 |
| hsa-miR-330-5p | LDLR | 1 | 1 | 1 | 1 | 4 |
| hsa-miR-330-5p | ARHGDIA | 1 | 1 | 1 | 1 | 4 |
| hsa-miR-330-5p | LIMK1 | 1 | 1 | 1 | 1 | 4 |
| hsa-miR-330-5p | FADS1 | 1 | 1 | 1 | 1 | 4 |
| hsa-miR-330-5p | FAM102A | 1 | 1 | 1 | 1 | 4 |
| hsa-miR-330-5p | C10orf114 | 1 | 1 | 1 | 1 | 4 |
| hsa-miR-330-5p | MED11 | 1 | 1 | 1 | 1 | 4 |
| hsa-miR-330-5p | LMX1A | 1 | 1 | 1 | 1 | 4 |
| hsa-miR-330-5p | PHOX2A | 1 | 1 | 1 | 1 | 4 |
| hsa-miR-330-5p | CRIP3 | 1 | 1 | 1 | 1 | 4 |
| hsa-miR-330-5p | KLHL31 | 1 | 1 | 1 | 1 | 4 |
| hsa-miR-330-5p | SAMD12 | 1 | 1 | 1 | 1 | 4 |
| hsa-miR-330-5p | SNX30 | 1 | 1 | 1 | 1 | 4 |
| hsa-miR-330-5p | LOXL2 | 1 | 1 | 1 | 1 | 4 |
| hsa-miR-330-5p | SRRD | 1 | 1 | 1 | 1 | 4 |
| hsa-miR-330-5p | LOC402665 | 1 | 1 | 1 | 1 | 4 |
| hsa-miR-330-5p | ARL3 | 1 | 1 | 1 | 1 | 4 |
| hsa-miR-330-5p | HAPLN4 | 1 | 1 | 1 | 1 | 4 |
| hsa-miR-330-5p | CUEDC1 | 1 | 1 | 1 | 1 | 4 |
| hsa-miR-330-5p | CD180 | 1 | 1 | 1 | 1 | 4 |
| hsa-miR-330-5p | SMAD4 | 1 | 1 | 1 | 1 | 4 |
| hsa-miR-330-5p | SMAD6 | 1 | 1 | 1 | 1 | 4 |
| hsa-miR-330-5p | ACCN2 | 1 | 1 | 1 | 1 | 4 |
| hsa-miR-330-5p | MAGEB4 | 1 | 1 | 1 | 1 | 4 |
| hsa-miR-330-5p | STS | 1 | 1 | 1 | 1 | 4 |
| hsa-miR-330-5p | MAN1A1 | 1 | 1 | 1 | 1 | 4 |
| hsa-miR-330-5p | MAN2A2 | 1 | 1 | 1 | 1 | 4 |
| hsa-miR-330-5p | MAOA | 1 | 1 | 1 | 1 | 4 |
| hsa-miR-330-5p | MAP1A | 1 | 1 | 1 | 1 | 4 |
| hsa-miR-330-5p | ARSD | 1 | 1 | 1 | 1 | 4 |
| hsa-miR-330-5p | DDI1 | 1 | 1 | 1 | 1 | 4 |
| hsa-miR-330-5p | LCN10 | 1 | 1 | 1 | 1 | 4 |
| hsa-miR-330-5p | MATK | 1 | 1 | 1 | 1 | 4 |
| hsa-miR-330-5p | MBP | 1 | 1 | 1 | 1 | 4 |
| hsa-miR-330-5p | MCC | 1 | 1 | 1 | 1 | 4 |
| hsa-miR-330-5p | MCM5 | 1 | 1 | 1 | 1 | 4 |
| hsa-miR-330-5p | MDK | 1 | 1 | 1 | 1 | 4 |
| hsa-miR-330-5p | MEF2D | 1 | 1 | 1 | 1 | 4 |
| hsa-miR-330-5p | ARVCF | 1 | 1 | 1 | 1 | 4 |
| hsa-miR-330-5p | MEOX1 | 1 | 1 | 1 | 1 | 4 |
| hsa-miR-330-5p | MEOX2 | 1 | 1 | 1 | 1 | 4 |
| hsa-miR-330-5p | MEST | 1 | 1 | 1 | 1 | 4 |
| hsa-miR-330-5p | MFGE8 | 1 | 1 | 1 | 1 | 4 |
| hsa-miR-330-5p | MFI2 | 1 | 1 | 1 | 1 | 4 |
| hsa-miR-330-5p | MFNG | 1 | 1 | 1 | 1 | 4 |
| hsa-miR-330-5p | KITLG | 1 | 1 | 1 | 1 | 4 |
| hsa-miR-330-5p | CXCL9 | 1 | 1 | 1 | 1 | 4 |
| hsa-miR-330-5p | MLL | 1 | 1 | 1 | 1 | 4 |
| hsa-miR-330-5p | AFF1 | 1 | 1 | 1 | 1 | 4 |
| hsa-miR-330-5p | MMP1 | 1 | 1 | 1 | 1 | 4 |
| hsa-miR-330-5p | MMP14 | 1 | 1 | 1 | 1 | 4 |
| hsa-miR-330-5p | MMP15 | 1 | 1 | 1 | 1 | 4 |
| hsa-miR-330-5p | MMP16 | 1 | 1 | 1 | 1 | 4 |
| hsa-miR-330-5p | MNT | 1 | 1 | 1 | 1 | 4 |
| hsa-miR-330-5p | MOBP | 1 | 1 | 1 | 1 | 4 |
| hsa-miR-330-5p | MPP3 | 1 | 1 | 1 | 1 | 4 |
| hsa-miR-330-5p | MPZ | 1 | 1 | 1 | 1 | 4 |
| hsa-miR-330-5p | CCDC88C | 1 | 1 | 1 | 1 | 4 |
| hsa-miR-330-5p | TRIM67 | 1 | 1 | 1 | 1 | 4 |
| hsa-miR-330-5p | MSN | 1 | 1 | 1 | 1 | 4 |
| hsa-miR-330-5p | MTHFR | 1 | 1 | 1 | 1 | 4 |
| hsa-miR-330-5p | ASTN1 | 1 | 1 | 1 | 1 | 4 |
| hsa-miR-330-5p | MX2 | 1 | 1 | 1 | 1 | 4 |
| hsa-miR-330-5p | MYBPH | 1 | 1 | 1 | 1 | 4 |
| hsa-miR-330-5p | MYD88 | 1 | 1 | 1 | 1 | 4 |
| hsa-miR-330-5p | MYLK | 1 | 1 | 1 | 1 | 4 |
| hsa-miR-330-5p | MYO1D | 1 | 1 | 1 | 1 | 4 |
| hsa-miR-330-5p | MYO5A | 1 | 1 | 1 | 1 | 4 |
| hsa-miR-330-5p | MYT1 | 1 | 1 | 1 | 1 | 4 |
| hsa-miR-330-5p | NAB2 | 1 | 1 | 1 | 1 | 4 |
| hsa-miR-330-5p | NAGA | 1 | 1 | 1 | 1 | 4 |
| hsa-miR-330-5p | NAGLU | 1 | 1 | 1 | 1 | 4 |
| hsa-miR-330-5p | ATF3 | 1 | 1 | 1 | 1 | 4 |
| hsa-miR-330-5p | NCF4 | 1 | 1 | 1 | 1 | 4 |
| hsa-miR-330-5p | NDN | 1 | 1 | 1 | 1 | 4 |
| hsa-miR-330-5p | NDUFA2 | 1 | 1 | 1 | 1 | 4 |
| hsa-miR-330-5p | ATM | 1 | 1 | 1 | 1 | 4 |
| hsa-miR-330-5p | NEDD8 | 1 | 1 | 1 | 1 | 4 |
| hsa-miR-330-5p | NEUROD2 | 1 | 1 | 1 | 1 | 4 |
| hsa-miR-330-5p | NFIX | 1 | 1 | 1 | 1 | 4 |
| hsa-miR-330-5p | ATP1A4 | 1 | 1 | 1 | 1 | 4 |
| hsa-miR-330-5p | NID1 | 1 | 1 | 1 | 1 | 4 |
| hsa-miR-330-5p | ATP1B2 | 1 | 1 | 1 | 1 | 4 |
| hsa-miR-330-5p | NMT1 | 1 | 1 | 1 | 1 | 4 |
| hsa-miR-330-5p | NOV | 1 | 1 | 1 | 1 | 4 |
| hsa-miR-330-5p | NPTX1 | 1 | 1 | 1 | 1 | 4 |
| hsa-miR-330-5p | NPTX2 | 1 | 1 | 1 | 1 | 4 |
| hsa-miR-330-5p | ATP2A3 | 1 | 1 | 1 | 1 | 4 |
| hsa-miR-330-5p | NRAP | 1 | 1 | 1 | 1 | 4 |
| hsa-miR-330-5p | NRAS | 1 | 1 | 1 | 1 | 4 |
| hsa-miR-330-5p | NSF | 1 | 1 | 1 | 1 | 4 |
| hsa-miR-330-5p | ATP2B3 | 1 | 1 | 1 | 1 | 4 |
| hsa-miR-330-5p | NTSR1 | 1 | 1 | 1 | 1 | 4 |
| hsa-miR-330-5p | NUCB1 | 1 | 1 | 1 | 1 | 4 |
| hsa-miR-330-5p | ATP4B | 1 | 1 | 1 | 1 | 4 |
| hsa-miR-330-5p | OLR1 | 1 | 1 | 1 | 1 | 4 |
| hsa-miR-330-5p | OVOL1 | 1 | 1 | 1 | 1 | 4 |
| hsa-miR-330-5p | P2RX4 | 1 | 1 | 1 | 1 | 4 |
| hsa-miR-330-5p | COL5A3 | 1 | 1 | 1 | 1 | 4 |
| hsa-miR-330-5p | PAPPA | 1 | 1 | 1 | 1 | 4 |
| hsa-miR-330-5p | DDEF1 | 1 | 1 | 1 | 1 | 4 |
| hsa-miR-330-5p | COPS7A | 1 | 1 | 1 | 1 | 4 |
| hsa-miR-330-5p | TRAT1 | 1 | 1 | 1 | 1 | 4 |
| hsa-miR-330-5p | STMN3 | 1 | 1 | 1 | 1 | 4 |
| hsa-miR-330-5p | PBX3 | 1 | 1 | 1 | 1 | 4 |
| hsa-miR-330-5p | CDON | 1 | 1 | 1 | 1 | 4 |
| hsa-miR-330-5p | PDE11A | 1 | 1 | 1 | 1 | 4 |
| hsa-miR-330-5p | SOST | 1 | 1 | 1 | 1 | 4 |
| hsa-miR-330-5p | ACOX1 | 1 | 1 | 1 | 1 | 4 |
| hsa-miR-330-5p | ASCC1 | 1 | 1 | 1 | 1 | 4 |
| hsa-miR-330-5p | VPS36 | 1 | 1 | 1 | 1 | 4 |
| hsa-miR-330-5p | FAM152A | 1 | 1 | 1 | 1 | 4 |
| hsa-miR-330-5p | ZBTB7B | 1 | 1 | 1 | 1 | 4 |
| hsa-miR-330-5p | ST8SIA3 | 1 | 1 | 1 | 1 | 4 |
| hsa-miR-330-5p | ZNF691 | 1 | 1 | 1 | 1 | 4 |
| hsa-miR-330-5p | YBX2 | 1 | 1 | 1 | 1 | 4 |
| hsa-miR-330-5p | C1orf66 | 1 | 1 | 1 | 1 | 4 |
| hsa-miR-330-5p | SH3GLB1 | 1 | 1 | 1 | 1 | 4 |
| hsa-miR-330-5p | METTL9 | 1 | 1 | 1 | 1 | 4 |
| hsa-miR-330-5p | PHF11 | 1 | 1 | 1 | 1 | 4 |
| hsa-miR-330-5p | MYO15A | 1 | 1 | 1 | 1 | 4 |
| hsa-miR-330-5p | LEF1 | 1 | 1 | 1 | 1 | 4 |
| hsa-miR-330-5p | CPA4 | 1 | 1 | 1 | 1 | 4 |
| hsa-miR-330-5p | PCSK1 | 1 | 1 | 1 | 1 | 4 |
| hsa-miR-330-5p | PHF20 | 1 | 1 | 1 | 1 | 4 |
| hsa-miR-330-5p | PCSK2 | 1 | 1 | 1 | 1 | 4 |
| hsa-miR-330-5p | UBAP1 | 1 | 1 | 1 | 1 | 4 |
| hsa-miR-330-5p | KLF3 | 1 | 1 | 1 | 1 | 4 |
| hsa-miR-330-5p | RBJ | 1 | 1 | 1 | 1 | 4 |
| hsa-miR-330-5p | IER5 | 1 | 1 | 1 | 1 | 4 |
| hsa-miR-330-5p | GOLM1 | 1 | 1 | 1 | 1 | 4 |
| hsa-miR-330-5p | RASL12 | 1 | 1 | 1 | 1 | 4 |
| hsa-miR-330-5p | GCNT4 | 1 | 1 | 1 | 1 | 4 |
| hsa-miR-330-5p | TLR8 | 1 | 1 | 1 | 1 | 4 |
| hsa-miR-330-5p | SLC25A37 | 1 | 1 | 1 | 1 | 4 |
| hsa-miR-330-5p | PHF21A | 1 | 1 | 1 | 1 | 4 |
| hsa-miR-330-5p | C8orf55 | 1 | 1 | 1 | 1 | 4 |
| hsa-miR-330-5p | DACT1 | 1 | 1 | 1 | 1 | 4 |
| hsa-miR-330-5p | CRNKL1 | 1 | 1 | 1 | 1 | 4 |
| hsa-miR-330-5p | ZBTB7A | 1 | 1 | 1 | 1 | 4 |
| hsa-miR-330-5p | WNT16 | 1 | 1 | 1 | 1 | 4 |
| hsa-miR-330-5p | BIN2 | 1 | 1 | 1 | 1 | 4 |
| hsa-miR-330-5p | SCARA3 | 1 | 1 | 1 | 1 | 4 |
| hsa-miR-330-5p | PCYOX1 | 1 | 1 | 1 | 1 | 4 |
| hsa-miR-330-5p | PRRX2 | 1 | 1 | 1 | 1 | 4 |
| hsa-miR-330-5p | RHCG | 1 | 1 | 1 | 1 | 4 |
| hsa-miR-330-5p | ANKFY1 | 1 | 1 | 1 | 1 | 4 |
| hsa-miR-330-5p | PDE6G | 1 | 1 | 1 | 1 | 4 |
| hsa-miR-330-5p | PTPLAD1 | 1 | 1 | 1 | 1 | 4 |
| hsa-miR-330-5p | C20orf111 | 1 | 1 | 1 | 1 | 4 |
| hsa-miR-330-5p | PDE1B | 1 | 1 | 1 | 1 | 4 |
| hsa-miR-330-5p | PDGFA | 1 | 1 | 1 | 1 | 4 |
| hsa-miR-330-5p | SIRT6 | 1 | 1 | 1 | 1 | 4 |
| hsa-miR-330-5p | RAB6B | 1 | 1 | 1 | 1 | 4 |
| hsa-miR-330-5p | HDAC7 | 1 | 1 | 1 | 1 | 4 |
| hsa-miR-330-5p | GDE1 | 1 | 1 | 1 | 1 | 4 |
| hsa-miR-330-5p | PDGFRB | 1 | 1 | 1 | 1 | 4 |
| hsa-miR-330-5p | KIAA0859 | 1 | 1 | 1 | 1 | 4 |
| hsa-miR-330-5p | KLF13 | 1 | 1 | 1 | 1 | 4 |
| hsa-miR-330-5p | PDK1 | 1 | 1 | 1 | 1 | 4 |
| hsa-miR-330-5p | PDK2 | 1 | 1 | 1 | 1 | 4 |
| hsa-miR-330-5p | TMBIM4 | 1 | 1 | 1 | 1 | 4 |
| hsa-miR-330-5p | ASB2 | 1 | 1 | 1 | 1 | 4 |
| hsa-miR-330-5p | SUFU | 1 | 1 | 1 | 1 | 4 |
| hsa-miR-330-5p | SELT | 1 | 1 | 1 | 1 | 4 |
| hsa-miR-330-5p | FBXO40 | 1 | 1 | 1 | 1 | 4 |
| hsa-miR-330-5p | UPB1 | 1 | 1 | 1 | 1 | 4 |
| hsa-miR-330-5p | CD244 | 1 | 1 | 1 | 1 | 4 |
| hsa-miR-330-5p | RP6-213H19.1 | 1 | 1 | 1 | 1 | 4 |
| hsa-miR-330-5p | JMJD1B | 1 | 1 | 1 | 1 | 4 |
| hsa-miR-330-5p | CECR1 | 1 | 1 | 1 | 1 | 4 |
| hsa-miR-330-5p | PER1 | 1 | 1 | 1 | 1 | 4 |
| hsa-miR-330-5p | ATP8B1 | 1 | 1 | 1 | 1 | 4 |
| hsa-miR-330-5p | PFKFB2 | 1 | 1 | 1 | 1 | 4 |
| hsa-miR-330-5p | PFKFB4 | 1 | 1 | 1 | 1 | 4 |
| hsa-miR-330-5p | ATP6V1A | 1 | 1 | 1 | 1 | 4 |
| hsa-miR-330-5p | PHF2 | 1 | 1 | 1 | 1 | 4 |
| hsa-miR-330-5p | PI3 | 1 | 1 | 1 | 1 | 4 |
| hsa-miR-330-5p | SERPINB5 | 1 | 1 | 1 | 1 | 4 |
| hsa-miR-330-5p | ATP6V1C1 | 1 | 1 | 1 | 1 | 4 |
| hsa-miR-330-5p | PIGR | 1 | 1 | 1 | 1 | 4 |
| hsa-miR-330-5p | ACP2 | 1 | 1 | 1 | 1 | 4 |
| hsa-miR-330-5p | PKD1 | 1 | 1 | 1 | 1 | 4 |
| hsa-miR-330-5p | CPXCR1 | 1 | 1 | 1 | 1 | 4 |
| hsa-miR-330-5p | BTBD1 | 1 | 1 | 1 | 1 | 4 |
| hsa-miR-330-5p | PLD2 | 1 | 1 | 1 | 1 | 4 |
| hsa-miR-330-5p | PLEC1 | 1 | 1 | 1 | 1 | 4 |
| hsa-miR-330-5p | PLEK | 1 | 1 | 1 | 1 | 4 |
| hsa-miR-330-5p | PLOD1 | 1 | 1 | 1 | 1 | 4 |
| hsa-miR-330-5p | PLS1 | 1 | 1 | 1 | 1 | 4 |
| hsa-miR-330-5p | PLXNA1 | 1 | 1 | 1 | 1 | 4 |
| hsa-miR-330-5p | EDG8 | 1 | 1 | 1 | 1 | 4 |
| hsa-miR-330-5p | PLXNB1 | 1 | 1 | 1 | 1 | 4 |
| hsa-miR-330-5p | PML | 1 | 1 | 1 | 1 | 4 |
| hsa-miR-330-5p | PMM1 | 1 | 1 | 1 | 1 | 4 |
| hsa-miR-330-5p | SLC37A1 | 1 | 1 | 1 | 1 | 4 |
| hsa-miR-330-5p | C21orf29 | 1 | 1 | 1 | 1 | 4 |
| hsa-miR-330-5p | SETD4 | 1 | 1 | 1 | 1 | 4 |
| hsa-miR-330-5p | CLIC6 | 1 | 1 | 1 | 1 | 4 |
| hsa-miR-330-5p | GPR88 | 1 | 1 | 1 | 1 | 4 |
| hsa-miR-330-5p | 4-Sep | 1 | 1 | 1 | 1 | 4 |
| hsa-miR-330-5p | POLE | 1 | 1 | 1 | 1 | 4 |
| hsa-miR-330-5p | POLR2C | 1 | 1 | 1 | 1 | 4 |
| hsa-miR-330-5p | GNG2 | 1 | 1 | 1 | 1 | 4 |
| hsa-miR-330-5p | POLR2E | 1 | 1 | 1 | 1 | 4 |
| hsa-miR-330-5p | WNT4 | 1 | 1 | 1 | 1 | 4 |
| hsa-miR-330-5p | SLC38A2 | 1 | 1 | 1 | 1 | 4 |
| hsa-miR-330-5p | SASH3 | 1 | 1 | 1 | 1 | 4 |
| hsa-miR-330-5p | SMCR7L | 1 | 1 | 1 | 1 | 4 |
| hsa-miR-330-5p | RNF216 | 1 | 1 | 1 | 1 | 4 |
| hsa-miR-330-5p | FAM64A | 1 | 1 | 1 | 1 | 4 |
| hsa-miR-330-5p | TXNDC10 | 1 | 1 | 1 | 1 | 4 |
| hsa-miR-330-5p | RBM47 | 1 | 1 | 1 | 1 | 4 |
| hsa-miR-330-5p | ANKRD16 | 1 | 1 | 1 | 1 | 4 |
| hsa-miR-330-5p | ROBO4 | 1 | 1 | 1 | 1 | 4 |
| hsa-miR-330-5p | CRCT1 | 1 | 1 | 1 | 1 | 4 |
| hsa-miR-330-5p | EPB41L4B | 1 | 1 | 1 | 1 | 4 |
| hsa-miR-330-5p | DLL4 | 1 | 1 | 1 | 1 | 4 |
| hsa-miR-330-5p | POU4F1 | 1 | 1 | 1 | 1 | 4 |
| hsa-miR-330-5p | UGT1A6 | 1 | 1 | 1 | 1 | 4 |
| hsa-miR-330-5p | INOC1 | 1 | 1 | 1 | 1 | 4 |
| hsa-miR-330-5p | HES2 | 1 | 1 | 1 | 1 | 4 |
| hsa-miR-330-5p | TBC1D13 | 1 | 1 | 1 | 1 | 4 |
| hsa-miR-330-5p | PPARD | 1 | 1 | 1 | 1 | 4 |
| hsa-miR-330-5p | GTPBP2 | 1 | 1 | 1 | 1 | 4 |
| hsa-miR-330-5p | SLC35F2 | 1 | 1 | 1 | 1 | 4 |
| hsa-miR-330-5p | IL17RD | 1 | 1 | 1 | 1 | 4 |
| hsa-miR-330-5p | PCSK4 | 1 | 1 | 1 | 1 | 4 |
| hsa-miR-330-5p | BNC2 | 1 | 1 | 1 | 1 | 4 |
| hsa-miR-330-5p | KLHL24 | 1 | 1 | 1 | 1 | 4 |
| hsa-miR-330-5p | CNNM2 | 1 | 1 | 1 | 1 | 4 |
| hsa-miR-330-5p | ZNF586 | 1 | 1 | 1 | 1 | 4 |
| hsa-miR-330-5p | QPCTL | 1 | 1 | 1 | 1 | 4 |
| hsa-miR-330-5p | NDE1 | 1 | 1 | 1 | 1 | 4 |
| hsa-miR-330-5p | BCAS3 | 1 | 1 | 1 | 1 | 4 |
| hsa-miR-330-5p | GDAP2 | 1 | 1 | 1 | 1 | 4 |
| hsa-miR-330-5p | SYTL2 | 1 | 1 | 1 | 1 | 4 |
| hsa-miR-330-5p | PGPEP1 | 1 | 1 | 1 | 1 | 4 |
| hsa-miR-330-5p | C9orf167 | 1 | 1 | 1 | 1 | 4 |
| hsa-miR-330-5p | TMEM104 | 1 | 1 | 1 | 1 | 4 |
| hsa-miR-330-5p | UHRF1BP1 | 1 | 1 | 1 | 1 | 4 |
| hsa-miR-330-5p | NSUN2 | 1 | 1 | 1 | 1 | 4 |
| hsa-miR-330-5p | CASZ1 | 1 | 1 | 1 | 1 | 4 |
| hsa-miR-330-5p | MKS1 | 1 | 1 | 1 | 1 | 4 |
| hsa-miR-330-5p | CYP2W1 | 1 | 1 | 1 | 1 | 4 |
| hsa-miR-330-5p | RPP25 | 1 | 1 | 1 | 1 | 4 |
| hsa-miR-330-5p | LIME1 | 1 | 1 | 1 | 1 | 4 |
| hsa-miR-330-5p | CHCHD3 | 1 | 1 | 1 | 1 | 4 |
| hsa-miR-330-5p | DUSP23 | 1 | 1 | 1 | 1 | 4 |
| hsa-miR-330-5p | C1orf27 | 1 | 1 | 1 | 1 | 4 |
| hsa-miR-330-5p | FAM120C | 1 | 1 | 1 | 1 | 4 |
| hsa-miR-330-5p | C1orf109 | 1 | 1 | 1 | 1 | 4 |
| hsa-miR-330-5p | PARP16 | 1 | 1 | 1 | 1 | 4 |
| hsa-miR-330-5p | C1orf56 | 1 | 1 | 1 | 1 | 4 |
| hsa-miR-330-5p | PIGX | 1 | 1 | 1 | 1 | 4 |
| hsa-miR-330-5p | TMEM70 | 1 | 1 | 1 | 1 | 4 |
| hsa-miR-330-5p | SLC25A38 | 1 | 1 | 1 | 1 | 4 |
| hsa-miR-330-5p | C2orf18 | 1 | 1 | 1 | 1 | 4 |
| hsa-miR-330-5p | CLN6 | 1 | 1 | 1 | 1 | 4 |
| hsa-miR-330-5p | AUP1 | 1 | 1 | 1 | 1 | 4 |
| hsa-miR-330-5p | VPS37C | 1 | 1 | 1 | 1 | 4 |
| hsa-miR-330-5p | C14orf102 | 1 | 1 | 1 | 1 | 4 |
| hsa-miR-330-5p | ATG16L1 | 1 | 1 | 1 | 1 | 4 |
| hsa-miR-330-5p | DET1 | 1 | 1 | 1 | 1 | 4 |
| hsa-miR-330-5p | IFT57 | 1 | 1 | 1 | 1 | 4 |
| hsa-miR-330-5p | PPP1R3D | 1 | 1 | 1 | 1 | 4 |
| hsa-miR-330-5p | AGGF1 | 1 | 1 | 1 | 1 | 4 |
| hsa-miR-330-5p | TRIM68 | 1 | 1 | 1 | 1 | 4 |
| hsa-miR-330-5p | ELP3 | 1 | 1 | 1 | 1 | 4 |
| hsa-miR-330-5p | PLEKHG6 | 1 | 1 | 1 | 1 | 4 |
| hsa-miR-330-5p | LGI2 | 1 | 1 | 1 | 1 | 4 |
| hsa-miR-330-5p | EXDL2 | 1 | 1 | 1 | 1 | 4 |
| hsa-miR-330-5p | LRRC20 | 1 | 1 | 1 | 1 | 4 |
| hsa-miR-330-5p | TRIM62 | 1 | 1 | 1 | 1 | 4 |
| hsa-miR-330-5p | PANK4 | 1 | 1 | 1 | 1 | 4 |
| hsa-miR-330-5p | C14orf115 | 1 | 1 | 1 | 1 | 4 |
| hsa-miR-330-5p | SLC38A7 | 1 | 1 | 1 | 1 | 4 |
| hsa-miR-330-5p | C7orf43 | 1 | 1 | 1 | 1 | 4 |
| hsa-miR-330-5p | C22orf26 | 1 | 1 | 1 | 1 | 4 |
| hsa-miR-330-5p | PPP2R5D | 1 | 1 | 1 | 1 | 4 |
| hsa-miR-330-5p | TMEM140 | 1 | 1 | 1 | 1 | 4 |
| hsa-miR-330-5p | C4orf19 | 1 | 1 | 1 | 1 | 4 |
| hsa-miR-330-5p | SPTLC3 | 1 | 1 | 1 | 1 | 4 |
| hsa-miR-330-5p | RSAD1 | 1 | 1 | 1 | 1 | 4 |
| hsa-miR-330-5p | C5orf22 | 1 | 1 | 1 | 1 | 4 |
| hsa-miR-330-5p | AGPAT5 | 1 | 1 | 1 | 1 | 4 |
| hsa-miR-330-5p | LIN7C | 1 | 1 | 1 | 1 | 4 |
| hsa-miR-330-5p | PPP3R2 | 1 | 1 | 1 | 1 | 4 |
| hsa-miR-330-5p | STK32B | 1 | 1 | 1 | 1 | 4 |
| hsa-miR-330-5p | PPP5C | 1 | 1 | 1 | 1 | 4 |
| hsa-miR-330-5p | IMPACT | 1 | 1 | 1 | 1 | 4 |
| hsa-miR-330-5p | MCM10 | 1 | 1 | 1 | 1 | 4 |
| hsa-miR-330-5p | SIRPG | 1 | 1 | 1 | 1 | 4 |
| hsa-miR-330-5p | HES6 | 1 | 1 | 1 | 1 | 4 |
| hsa-miR-330-5p | TNFRSF19 | 1 | 1 | 1 | 1 | 4 |
| hsa-miR-330-5p | TMEM55A | 1 | 1 | 1 | 1 | 4 |
| hsa-miR-330-5p | SVOP | 1 | 1 | 1 | 1 | 4 |
| hsa-miR-330-5p | ELMOD1 | 1 | 1 | 1 | 1 | 4 |
| hsa-miR-330-5p | GALNT10 | 1 | 1 | 1 | 1 | 4 |
| hsa-miR-330-5p | MED29 | 1 | 1 | 1 | 1 | 4 |
| hsa-miR-330-5p | OTUD5 | 1 | 1 | 1 | 1 | 4 |
| hsa-miR-330-5p | ZNF280C | 1 | 1 | 1 | 1 | 4 |
| hsa-miR-330-5p | FERMT1 | 1 | 1 | 1 | 1 | 4 |
| hsa-miR-330-5p | MTMR8 | 1 | 1 | 1 | 1 | 4 |
| hsa-miR-330-5p | AMBRA1 | 1 | 1 | 1 | 1 | 4 |
| hsa-miR-330-5p | BTBD2 | 1 | 1 | 1 | 1 | 4 |
| hsa-miR-330-5p | PRKAB2 | 1 | 1 | 1 | 1 | 4 |
| hsa-miR-330-5p | FLJ20489 | 1 | 1 | 1 | 1 | 4 |
| hsa-miR-330-5p | TMEM127 | 1 | 1 | 1 | 1 | 4 |
| hsa-miR-330-5p | PRKACA | 1 | 1 | 1 | 1 | 4 |
| hsa-miR-330-5p | LIMS2 | 1 | 1 | 1 | 1 | 4 |
| hsa-miR-330-5p | PRKACG | 1 | 1 | 1 | 1 | 4 |
| hsa-miR-330-5p | RUFY2 | 1 | 1 | 1 | 1 | 4 |
| hsa-miR-330-5p | PACS1 | 1 | 1 | 1 | 1 | 4 |
| hsa-miR-330-5p | FLJ10357 | 1 | 1 | 1 | 1 | 4 |
| hsa-miR-330-5p | IPO9 | 1 | 1 | 1 | 1 | 4 |
| hsa-miR-330-5p | DOK4 | 1 | 1 | 1 | 1 | 4 |
| hsa-miR-330-5p | ASF1B | 1 | 1 | 1 | 1 | 4 |
| hsa-miR-330-5p | BTBD7 | 1 | 1 | 1 | 1 | 4 |
| hsa-miR-330-5p | ZFP64 | 1 | 1 | 1 | 1 | 4 |
| hsa-miR-330-5p | DNAJC11 | 1 | 1 | 1 | 1 | 4 |
| hsa-miR-330-5p | ENAH | 1 | 1 | 1 | 1 | 4 |
| hsa-miR-330-5p | PARVA | 1 | 1 | 1 | 1 | 4 |
| hsa-miR-330-5p | C14orf108 | 1 | 1 | 1 | 1 | 4 |
| hsa-miR-330-5p | PRKAR2A | 1 | 1 | 1 | 1 | 4 |
| hsa-miR-330-5p | MBD5 | 1 | 1 | 1 | 1 | 4 |
| hsa-miR-330-5p | C14orf131 | 1 | 1 | 1 | 1 | 4 |
| hsa-miR-330-5p | PRKCE | 1 | 1 | 1 | 1 | 4 |
| hsa-miR-330-5p | PRKCG | 1 | 1 | 1 | 1 | 4 |
| hsa-miR-330-5p | PAG1 | 1 | 1 | 1 | 1 | 4 |
| hsa-miR-330-5p | WWC3 | 1 | 1 | 1 | 1 | 4 |
| hsa-miR-330-5p | TEX2 | 1 | 1 | 1 | 1 | 4 |
| hsa-miR-330-5p | PKN2 | 1 | 1 | 1 | 1 | 4 |
| hsa-miR-330-5p | SLC22A11 | 1 | 1 | 1 | 1 | 4 |
| hsa-miR-330-5p | ASH1L | 1 | 1 | 1 | 1 | 4 |
| hsa-miR-330-5p | ZNF395 | 1 | 1 | 1 | 1 | 4 |
| hsa-miR-330-5p | KIAA1166 | 1 | 1 | 1 | 1 | 4 |
| hsa-miR-330-5p | APOB48R | 1 | 1 | 1 | 1 | 4 |
| hsa-miR-330-5p | ERBB2IP | 1 | 1 | 1 | 1 | 4 |
| hsa-miR-330-5p | MAPK1 | 1 | 1 | 1 | 1 | 4 |
| hsa-miR-330-5p | AJAP1 | 1 | 1 | 1 | 1 | 4 |
| hsa-miR-330-5p | NSFL1C | 1 | 1 | 1 | 1 | 4 |
| hsa-miR-330-5p | MAPK11 | 1 | 1 | 1 | 1 | 4 |
| hsa-miR-330-5p | C19orf61 | 1 | 1 | 1 | 1 | 4 |
| hsa-miR-330-5p | PDGFC | 1 | 1 | 1 | 1 | 4 |
| hsa-miR-330-5p | MLZE | 1 | 1 | 1 | 1 | 4 |
| hsa-miR-330-5p | SUSD2 | 1 | 1 | 1 | 1 | 4 |
| hsa-miR-330-5p | PRODH | 1 | 1 | 1 | 1 | 4 |
| hsa-miR-330-5p | TXNDC13 | 1 | 1 | 1 | 1 | 4 |
| hsa-miR-330-5p | CABP5 | 1 | 1 | 1 | 1 | 4 |
| hsa-miR-330-5p | INPP5E | 1 | 1 | 1 | 1 | 4 |
| hsa-miR-330-5p | EIF5A2 | 1 | 1 | 1 | 1 | 4 |
| hsa-miR-330-5p | TMPRSS4 | 1 | 1 | 1 | 1 | 4 |
| hsa-miR-330-5p | PANX2 | 1 | 1 | 1 | 1 | 4 |
| hsa-miR-330-5p | MUC13 | 1 | 1 | 1 | 1 | 4 |
| hsa-miR-330-5p | NRIP3 | 1 | 1 | 1 | 1 | 4 |
| hsa-miR-330-5p | PSKH1 | 1 | 1 | 1 | 1 | 4 |
| hsa-miR-330-5p | AGPAT3 | 1 | 1 | 1 | 1 | 4 |
| hsa-miR-330-5p | AGPAT4 | 1 | 1 | 1 | 1 | 4 |
| hsa-miR-330-5p | DHX33 | 1 | 1 | 1 | 1 | 4 |
| hsa-miR-330-5p | NCLN | 1 | 1 | 1 | 1 | 4 |
| hsa-miR-330-5p | NT5M | 1 | 1 | 1 | 1 | 4 |
| hsa-miR-330-5p | RGMA | 1 | 1 | 1 | 1 | 4 |
| hsa-miR-330-5p | PRDM10 | 1 | 1 | 1 | 1 | 4 |
| hsa-miR-330-5p | KTELC1 | 1 | 1 | 1 | 1 | 4 |
| hsa-miR-330-5p | TULP4 | 1 | 1 | 1 | 1 | 4 |
| hsa-miR-330-5p | AGTRAP | 1 | 1 | 1 | 1 | 4 |
| hsa-miR-330-5p | ENTPD7 | 1 | 1 | 1 | 1 | 4 |
| hsa-miR-330-5p | PCNP | 1 | 1 | 1 | 1 | 4 |
| hsa-miR-330-5p | C1orf128 | 1 | 1 | 1 | 1 | 4 |
| hsa-miR-330-5p | PARP11 | 1 | 1 | 1 | 1 | 4 |
| hsa-miR-330-5p | TMEM16B | 1 | 1 | 1 | 1 | 4 |
| hsa-miR-330-5p | NAT14 | 1 | 1 | 1 | 1 | 4 |
| hsa-miR-330-5p | REXO4 | 1 | 1 | 1 | 1 | 4 |
| hsa-miR-330-5p | LYRM4 | 1 | 1 | 1 | 1 | 4 |
| hsa-miR-330-5p | C20orf3 | 1 | 1 | 1 | 1 | 4 |
| hsa-miR-330-5p | ADCK1 | 1 | 1 | 1 | 1 | 4 |
| hsa-miR-330-5p | KIAA1219 | 1 | 1 | 1 | 1 | 4 |
| hsa-miR-330-5p | TMEM63C | 1 | 1 | 1 | 1 | 4 |
| hsa-miR-330-5p | JPH2 | 1 | 1 | 1 | 1 | 4 |
| hsa-miR-330-5p | PELI2 | 1 | 1 | 1 | 1 | 4 |
| hsa-miR-330-5p | GJC2 | 1 | 1 | 1 | 1 | 4 |
| hsa-miR-330-5p | ASPHD2 | 1 | 1 | 1 | 1 | 4 |
| hsa-miR-330-5p | DOLPP1 | 1 | 1 | 1 | 1 | 4 |
| hsa-miR-330-5p | CORO1B | 1 | 1 | 1 | 1 | 4 |
| hsa-miR-330-5p | ZMIZ1 | 1 | 1 | 1 | 1 | 4 |
| hsa-miR-330-5p | ACTR3B | 1 | 1 | 1 | 1 | 4 |
| hsa-miR-330-5p | KIAA1147 | 1 | 1 | 1 | 1 | 4 |
| hsa-miR-330-5p | ATP8B2 | 1 | 1 | 1 | 1 | 4 |
| hsa-miR-330-5p | ATP10D | 1 | 1 | 1 | 1 | 4 |
| hsa-miR-330-5p | KIAA1199 | 1 | 1 | 1 | 1 | 4 |
| hsa-miR-330-5p | VANGL2 | 1 | 1 | 1 | 1 | 4 |
| hsa-miR-330-5p | LOC57228 | 1 | 1 | 1 | 1 | 4 |
| hsa-miR-330-5p | RCN3 | 1 | 1 | 1 | 1 | 4 |
| hsa-miR-330-5p | PTGFR | 1 | 1 | 1 | 1 | 4 |
| hsa-miR-330-5p | MRS2 | 1 | 1 | 1 | 1 | 4 |
| hsa-miR-330-5p | PTGIR | 1 | 1 | 1 | 1 | 4 |
| hsa-miR-330-5p | PTGIS | 1 | 1 | 1 | 1 | 4 |
| hsa-miR-330-5p | RAB22A | 1 | 1 | 1 | 1 | 4 |
| hsa-miR-330-5p | ABHD6 | 1 | 1 | 1 | 1 | 4 |
| hsa-miR-330-5p | SLC24A3 | 1 | 1 | 1 | 1 | 4 |
| hsa-miR-330-5p | PTGS1 | 1 | 1 | 1 | 1 | 4 |
| hsa-miR-330-5p | PTGS2 | 1 | 1 | 1 | 1 | 4 |
| hsa-miR-330-5p | GALNTL1 | 1 | 1 | 1 | 1 | 4 |
| hsa-miR-330-5p | REXO1 | 1 | 1 | 1 | 1 | 4 |
| hsa-miR-330-5p | KIAA1161 | 1 | 1 | 1 | 1 | 4 |
| hsa-miR-330-5p | CNOT6 | 1 | 1 | 1 | 1 | 4 |
| hsa-miR-330-5p | ZNF512B | 1 | 1 | 1 | 1 | 4 |
| hsa-miR-330-5p | ZNF490 | 1 | 1 | 1 | 1 | 4 |
| hsa-miR-330-5p | PLEKHH1 | 1 | 1 | 1 | 1 | 4 |
| hsa-miR-330-5p | SHROOM4 | 1 | 1 | 1 | 1 | 4 |
| hsa-miR-330-5p | RNF150 | 1 | 1 | 1 | 1 | 4 |
| hsa-miR-330-5p | FAM62B | 1 | 1 | 1 | 1 | 4 |
| hsa-miR-330-5p | AHRR | 1 | 1 | 1 | 1 | 4 |
| hsa-miR-330-5p | KIAA1257 | 1 | 1 | 1 | 1 | 4 |
| hsa-miR-330-5p | ZNF608 | 1 | 1 | 1 | 1 | 4 |
| hsa-miR-330-5p | MTUS1 | 1 | 1 | 1 | 1 | 4 |
| hsa-miR-330-5p | PCDH19 | 1 | 1 | 1 | 1 | 4 |
| hsa-miR-330-5p | KIAA1328 | 1 | 1 | 1 | 1 | 4 |
| hsa-miR-330-5p | SORCS2 | 1 | 1 | 1 | 1 | 4 |
| hsa-miR-330-5p | ALPK3 | 1 | 1 | 1 | 1 | 4 |
| hsa-miR-330-5p | PTCHD2 | 1 | 1 | 1 | 1 | 4 |
| hsa-miR-330-5p | NLGN2 | 1 | 1 | 1 | 1 | 4 |
| hsa-miR-330-5p | KLHL14 | 1 | 1 | 1 | 1 | 4 |
| hsa-miR-330-5p | ZNF319 | 1 | 1 | 1 | 1 | 4 |
| hsa-miR-330-5p | TRMT5 | 1 | 1 | 1 | 1 | 4 |
| hsa-miR-330-5p | 4-Mar | 1 | 1 | 1 | 1 | 4 |
| hsa-miR-330-5p | KIF17 | 1 | 1 | 1 | 1 | 4 |
| hsa-miR-330-5p | KIAA1409 | 1 | 1 | 1 | 1 | 4 |
| hsa-miR-330-5p | PITPNM2 | 1 | 1 | 1 | 1 | 4 |
| hsa-miR-330-5p | RANBP10 | 1 | 1 | 1 | 1 | 4 |
| hsa-miR-330-5p | EP400 | 1 | 1 | 1 | 1 | 4 |
| hsa-miR-330-5p | ZSWIM5 | 1 | 1 | 1 | 1 | 4 |
| hsa-miR-330-5p | DHX37 | 1 | 1 | 1 | 1 | 4 |
| hsa-miR-330-5p | EPB41L5 | 1 | 1 | 1 | 1 | 4 |
| hsa-miR-330-5p | KIAA1553 | 1 | 1 | 1 | 1 | 4 |
| hsa-miR-330-5p | CACHD1 | 1 | 1 | 1 | 1 | 4 |
| hsa-miR-330-5p | DENND1A | 1 | 1 | 1 | 1 | 4 |
| hsa-miR-330-5p | SEMA4G | 1 | 1 | 1 | 1 | 4 |
| hsa-miR-330-5p | GPR107 | 1 | 1 | 1 | 1 | 4 |
| hsa-miR-330-5p | KIAA1632 | 1 | 1 | 1 | 1 | 4 |
| hsa-miR-330-5p | ZFYVE28 | 1 | 1 | 1 | 1 | 4 |
| hsa-miR-330-5p | PTPN3 | 1 | 1 | 1 | 1 | 4 |
| hsa-miR-330-5p | TRIB3 | 1 | 1 | 1 | 1 | 4 |
| hsa-miR-330-5p | RAB40C | 1 | 1 | 1 | 1 | 4 |
| hsa-miR-330-5p | POLD4 | 1 | 1 | 1 | 1 | 4 |
| hsa-miR-330-5p | SLAMF7 | 1 | 1 | 1 | 1 | 4 |
| hsa-miR-330-5p | HMHB1 | 1 | 1 | 1 | 1 | 4 |
| hsa-miR-330-5p | PTPRD | 1 | 1 | 1 | 1 | 4 |
| hsa-miR-330-5p | PTPRM | 1 | 1 | 1 | 1 | 4 |
| hsa-miR-330-5p | PTPRN | 1 | 1 | 1 | 1 | 4 |
| hsa-miR-330-5p | PTPRN2 | 1 | 1 | 1 | 1 | 4 |
| hsa-miR-330-5p | PTX3 | 1 | 1 | 1 | 1 | 4 |
| hsa-miR-330-5p | NGB | 1 | 1 | 1 | 1 | 4 |
| hsa-miR-330-5p | PVR | 1 | 1 | 1 | 1 | 4 |
| hsa-miR-330-5p | PVRL1 | 1 | 1 | 1 | 1 | 4 |
| hsa-miR-330-5p | WFDC1 | 1 | 1 | 1 | 1 | 4 |
| hsa-miR-330-5p | CTDSP1 | 1 | 1 | 1 | 1 | 4 |
| hsa-miR-330-5p | BBS1 | 1 | 1 | 1 | 1 | 4 |
| hsa-miR-330-5p | PYGB | 1 | 1 | 1 | 1 | 4 |
| hsa-miR-330-5p | ENOPH1 | 1 | 1 | 1 | 1 | 4 |
| hsa-miR-330-5p | RHOU | 1 | 1 | 1 | 1 | 4 |
| hsa-miR-330-5p | TRAPPC1 | 1 | 1 | 1 | 1 | 4 |
| hsa-miR-330-5p | PRUNE | 1 | 1 | 1 | 1 | 4 |
| hsa-miR-330-5p | C19orf29 | 1 | 1 | 1 | 1 | 4 |
| hsa-miR-330-5p | RBM25 | 1 | 1 | 1 | 1 | 4 |
| hsa-miR-330-5p | BCAT1 | 1 | 1 | 1 | 1 | 4 |
| hsa-miR-330-5p | RAB5B | 1 | 1 | 1 | 1 | 4 |
| hsa-miR-330-5p | RAD9A | 1 | 1 | 1 | 1 | 4 |
| hsa-miR-330-5p | RAG1 | 1 | 1 | 1 | 1 | 4 |
| hsa-miR-330-5p | ENPP5 | 1 | 1 | 1 | 1 | 4 |
| hsa-miR-330-5p | RAP2B | 1 | 1 | 1 | 1 | 4 |
| hsa-miR-330-5p | ACE2 | 1 | 1 | 1 | 1 | 4 |
| hsa-miR-330-5p | CACNG7 | 1 | 1 | 1 | 1 | 4 |
| hsa-miR-330-5p | CACNG6 | 1 | 1 | 1 | 1 | 4 |
| hsa-miR-330-5p | RBBP5 | 1 | 1 | 1 | 1 | 4 |
| hsa-miR-330-5p | TRPV4 | 1 | 1 | 1 | 1 | 4 |
| hsa-miR-330-5p | GNB4 | 1 | 1 | 1 | 1 | 4 |
| hsa-miR-330-5p | LGR6 | 1 | 1 | 1 | 1 | 4 |
| hsa-miR-330-5p | CCND1 | 1 | 1 | 1 | 1 | 4 |
| hsa-miR-330-5p | BCL2 | 1 | 1 | 1 | 1 | 4 |
| hsa-miR-330-5p | DPF2 | 1 | 1 | 1 | 1 | 4 |
| hsa-miR-330-5p | RET | 1 | 1 | 1 | 1 | 4 |
| hsa-miR-330-5p | BCL2L1 | 1 | 1 | 1 | 1 | 4 |
| hsa-miR-330-5p | RFC1 | 1 | 1 | 1 | 1 | 4 |
| hsa-miR-330-5p | BCL2L2 | 1 | 1 | 1 | 1 | 4 |
| hsa-miR-330-5p | RGS3 | 1 | 1 | 1 | 1 | 4 |
| hsa-miR-330-5p | RGS16 | 1 | 1 | 1 | 1 | 4 |
| hsa-miR-330-5p | BCL3 | 1 | 1 | 1 | 1 | 4 |
| hsa-miR-330-5p | RNASEL | 1 | 1 | 1 | 1 | 4 |
| hsa-miR-330-5p | TGIF2 | 1 | 1 | 1 | 1 | 4 |
| hsa-miR-330-5p | RNF2 | 1 | 1 | 1 | 1 | 4 |
| hsa-miR-330-5p | BACH2 | 1 | 1 | 1 | 1 | 4 |
| hsa-miR-330-5p | PPCDC | 1 | 1 | 1 | 1 | 4 |
| hsa-miR-330-5p | NYX | 1 | 1 | 1 | 1 | 4 |
| hsa-miR-330-5p | RIC8A | 1 | 1 | 1 | 1 | 4 |
| hsa-miR-330-5p | RORC | 1 | 1 | 1 | 1 | 4 |
| hsa-miR-330-5p | RPE65 | 1 | 1 | 1 | 1 | 4 |
| hsa-miR-330-5p | BCR | 1 | 1 | 1 | 1 | 4 |
| hsa-miR-330-5p | RPL10 | 1 | 1 | 1 | 1 | 4 |
| hsa-miR-330-5p | RPS6KA3 | 1 | 1 | 1 | 1 | 4 |
| hsa-miR-330-5p | RPS6KB2 | 1 | 1 | 1 | 1 | 4 |
| hsa-miR-330-5p | BDKRB2 | 1 | 1 | 1 | 1 | 4 |
| hsa-miR-330-5p | RS1 | 1 | 1 | 1 | 1 | 4 |
| hsa-miR-330-5p | RXRA | 1 | 1 | 1 | 1 | 4 |
| hsa-miR-330-5p | S100A3 | 1 | 1 | 1 | 1 | 4 |
| hsa-miR-330-5p | SCN2B | 1 | 1 | 1 | 1 | 4 |
| hsa-miR-330-5p | SCN3A | 1 | 1 | 1 | 1 | 4 |
| hsa-miR-330-5p | SCN4A | 1 | 1 | 1 | 1 | 4 |
| hsa-miR-330-5p | BGN | 1 | 1 | 1 | 1 | 4 |
| hsa-miR-330-5p | SCN4B | 1 | 1 | 1 | 1 | 4 |
| hsa-miR-330-5p | SCNN1B | 1 | 1 | 1 | 1 | 4 |
| hsa-miR-330-5p | SCNN1G | 1 | 1 | 1 | 1 | 4 |
| hsa-miR-330-5p | SRL | 1 | 1 | 1 | 1 | 4 |
| hsa-miR-330-5p | BHMT | 1 | 1 | 1 | 1 | 4 |
| hsa-miR-330-5p | CCL21 | 1 | 1 | 1 | 1 | 4 |
| hsa-miR-330-5p | CXCL5 | 1 | 1 | 1 | 1 | 4 |
| hsa-miR-330-5p | CX3CL1 | 1 | 1 | 1 | 1 | 4 |
| hsa-miR-330-5p | CXCL12 | 1 | 1 | 1 | 1 | 4 |
| hsa-miR-330-5p | PKNOX2 | 1 | 1 | 1 | 1 | 4 |
| hsa-miR-330-5p | RNF123 | 1 | 1 | 1 | 1 | 4 |
| hsa-miR-330-5p | SDHC | 1 | 1 | 1 | 1 | 4 |
| hsa-miR-330-5p | MUTED | 1 | 1 | 1 | 1 | 4 |
| hsa-miR-330-5p | DMRTB1 | 1 | 1 | 1 | 1 | 4 |
| hsa-miR-330-5p | SEC14L1 | 1 | 1 | 1 | 1 | 4 |
| hsa-miR-330-5p | P53AIP1 | 1 | 1 | 1 | 1 | 4 |
| hsa-miR-330-5p | KIF13A | 1 | 1 | 1 | 1 | 4 |
| hsa-miR-330-5p | PRDM16 | 1 | 1 | 1 | 1 | 4 |
| hsa-miR-330-5p | TMEM16C | 1 | 1 | 1 | 1 | 4 |
| hsa-miR-330-5p | SMOC1 | 1 | 1 | 1 | 1 | 4 |
| hsa-miR-330-5p | TMBIM1 | 1 | 1 | 1 | 1 | 4 |
| hsa-miR-330-5p | C10orf54 | 1 | 1 | 1 | 1 | 4 |
| hsa-miR-330-5p | NOD2 | 1 | 1 | 1 | 1 | 4 |
| hsa-miR-330-5p | XYLT1 | 1 | 1 | 1 | 1 | 4 |
| hsa-miR-330-5p | SFRP1 | 1 | 1 | 1 | 1 | 4 |
| hsa-miR-330-5p | ABCG5 | 1 | 1 | 1 | 1 | 4 |
| hsa-miR-330-5p | SFRP5 | 1 | 1 | 1 | 1 | 4 |
| hsa-miR-330-5p | PAPD5 | 1 | 1 | 1 | 1 | 4 |
| hsa-miR-330-5p | FBRS | 1 | 1 | 1 | 1 | 4 |
| hsa-miR-330-5p | ZFP106 | 1 | 1 | 1 | 1 | 4 |
| hsa-miR-330-5p | MPP5 | 1 | 1 | 1 | 1 | 4 |
| hsa-miR-330-5p | CDH22 | 1 | 1 | 1 | 1 | 4 |
| hsa-miR-330-5p | RGS18 | 1 | 1 | 1 | 1 | 4 |
| hsa-miR-330-5p | WBSCR17 | 1 | 1 | 1 | 1 | 4 |
| hsa-miR-330-5p | KLHL25 | 1 | 1 | 1 | 1 | 4 |
| hsa-miR-330-5p | GZF1 | 1 | 1 | 1 | 1 | 4 |
| hsa-miR-330-5p | MRPS25 | 1 | 1 | 1 | 1 | 4 |
| hsa-miR-330-5p | NOM1 | 1 | 1 | 1 | 1 | 4 |
| hsa-miR-330-5p | C15orf56 | 1 | 1 | 1 | 1 | 4 |
| hsa-miR-330-5p | SH3BGR | 1 | 1 | 1 | 1 | 4 |
| hsa-miR-330-5p | PABPC1L2B | 1 | 1 | 1 | 1 | 4 |
| hsa-miR-330-5p | PLA2G2F | 1 | 1 | 1 | 1 | 4 |
| hsa-miR-330-5p | SHB | 1 | 1 | 1 | 1 | 4 |
| hsa-miR-330-5p | AXUD1 | 1 | 1 | 1 | 1 | 4 |
| hsa-miR-330-5p | FBXW4 | 1 | 1 | 1 | 1 | 4 |
| hsa-miR-330-5p | COPS7B | 1 | 1 | 1 | 1 | 4 |
| hsa-miR-330-5p | ATPAF1 | 1 | 1 | 1 | 1 | 4 |
| hsa-miR-330-5p | RAI16 | 1 | 1 | 1 | 1 | 4 |
| hsa-miR-330-5p | CREB3L2 | 1 | 1 | 1 | 1 | 4 |
| hsa-miR-330-5p | S100PBP | 1 | 1 | 1 | 1 | 4 |
| hsa-miR-330-5p | LMF1 | 1 | 1 | 1 | 1 | 4 |
| hsa-miR-330-5p | KLC2 | 1 | 1 | 1 | 1 | 4 |
| hsa-miR-330-5p | FNDC4 | 1 | 1 | 1 | 1 | 4 |
| hsa-miR-330-5p | FAM129B | 1 | 1 | 1 | 1 | 4 |
| hsa-miR-330-5p | VWA1 | 1 | 1 | 1 | 1 | 4 |
| hsa-miR-330-5p | ST3GAL3 | 1 | 1 | 1 | 1 | 4 |
| hsa-miR-330-5p | C12orf43 | 1 | 1 | 1 | 1 | 4 |
| hsa-miR-330-5p | AGXT2 | 1 | 1 | 1 | 1 | 4 |
| hsa-miR-330-5p | LRRC19 | 1 | 1 | 1 | 1 | 4 |
| hsa-miR-330-5p | SLC1A4 | 1 | 1 | 1 | 1 | 4 |
| hsa-miR-330-5p | INTS3 | 1 | 1 | 1 | 1 | 4 |
| hsa-miR-330-5p | SLC2A4 | 1 | 1 | 1 | 1 | 4 |
| hsa-miR-330-5p | SLC4A1 | 1 | 1 | 1 | 1 | 4 |
| hsa-miR-330-5p | SPATS2 | 1 | 1 | 1 | 1 | 4 |
| hsa-miR-330-5p | PYCRL | 1 | 1 | 1 | 1 | 4 |
| hsa-miR-330-5p | ZBTB8 | 1 | 1 | 1 | 1 | 4 |
| hsa-miR-330-5p | LOC653319 | 1 | 1 | 1 | 1 | 4 |
| hsa-miR-330-5p | SLC7A1 | 1 | 1 | 1 | 1 | 4 |
| hsa-miR-330-5p | SLC7A2 | 1 | 1 | 1 | 1 | 4 |
| hsa-miR-330-5p | SLC7A4 | 1 | 1 | 1 | 1 | 4 |
| hsa-miR-330-5p | SLC9A1 | 1 | 1 | 1 | 1 | 4 |
| hsa-miR-330-5p | SLC9A5 | 1 | 1 | 1 | 1 | 4 |
| hsa-miR-330-5p | SLC12A2 | 1 | 1 | 1 | 1 | 4 |
| hsa-miR-330-5p | SLC15A2 | 1 | 1 | 1 | 1 | 4 |
| hsa-miR-330-5p | SLC20A2 | 1 | 1 | 1 | 1 | 4 |
| hsa-miR-330-5p | KCTD14 | 1 | 1 | 1 | 1 | 4 |
| hsa-miR-330-5p | ZNF747 | 1 | 1 | 1 | 1 | 4 |
| hsa-miR-330-5p | LRRC61 | 1 | 1 | 1 | 1 | 4 |
| hsa-miR-330-5p | CHID1 | 1 | 1 | 1 | 1 | 4 |
| hsa-miR-330-5p | MTMR9 | 1 | 1 | 1 | 1 | 4 |
| hsa-miR-330-5p | SMO | 1 | 1 | 1 | 1 | 4 |
| hsa-miR-330-5p | SNAPC3 | 1 | 1 | 1 | 1 | 4 |
| hsa-miR-330-5p | FSCN1 | 1 | 1 | 1 | 1 | 4 |
| hsa-miR-330-5p | SNRPA | 1 | 1 | 1 | 1 | 4 |
| hsa-miR-330-5p | SNTA1 | 1 | 1 | 1 | 1 | 4 |
| hsa-miR-330-5p | SNTB1 | 1 | 1 | 1 | 1 | 4 |
| hsa-miR-330-5p | SNX1 | 1 | 1 | 1 | 1 | 4 |
| hsa-miR-330-5p | SOD3 | 1 | 1 | 1 | 1 | 4 |
| hsa-miR-330-5p | SOLH | 1 | 1 | 1 | 1 | 4 |
| hsa-miR-330-5p | SOX10 | 1 | 1 | 1 | 1 | 4 |
| hsa-miR-330-5p | SOX12 | 1 | 1 | 1 | 1 | 4 |
| hsa-miR-330-5p | SPI1 | 1 | 1 | 1 | 1 | 4 |
| hsa-miR-330-5p | SPN | 1 | 1 | 1 | 1 | 4 |
| hsa-miR-330-5p | SPR | 1 | 1 | 1 | 1 | 4 |
| hsa-miR-330-5p | SRD5A2 | 1 | 1 | 1 | 1 | 4 |
| hsa-miR-330-5p | SRF | 1 | 1 | 1 | 1 | 4 |
| hsa-miR-330-5p | SSR1 | 1 | 1 | 1 | 1 | 4 |
| hsa-miR-330-5p | SSTR1 | 1 | 1 | 1 | 1 | 4 |
| hsa-miR-330-5p | STAT2 | 1 | 1 | 1 | 1 | 4 |
| hsa-miR-330-5p | STAT5B | 1 | 1 | 1 | 1 | 4 |
| hsa-miR-330-5p | STC1 | 1 | 1 | 1 | 1 | 4 |
| hsa-miR-330-5p | ELOVL4 | 1 | 1 | 1 | 1 | 4 |
| hsa-miR-330-5p | STIM1 | 1 | 1 | 1 | 1 | 4 |
| hsa-miR-330-5p | STK4 | 1 | 1 | 1 | 1 | 4 |
| hsa-miR-330-5p | STK10 | 1 | 1 | 1 | 1 | 4 |
| hsa-miR-330-5p | STK11 | 1 | 1 | 1 | 1 | 4 |
| hsa-miR-330-5p | STX3 | 1 | 1 | 1 | 1 | 4 |
| hsa-miR-330-5p | SUPT4H1 | 1 | 1 | 1 | 1 | 4 |
| hsa-miR-330-5p | MED22 | 1 | 1 | 1 | 1 | 4 |
| hsa-miR-330-5p | SUV39H1 | 1 | 1 | 1 | 1 | 4 |
| hsa-miR-330-5p | SYN1 | 1 | 1 | 1 | 1 | 4 |
| hsa-miR-330-5p | SYT4 | 1 | 1 | 1 | 1 | 4 |
| hsa-miR-330-5p | T | 1 | 1 | 1 | 1 | 4 |
| hsa-miR-330-5p | TAF12 | 1 | 1 | 1 | 1 | 4 |
| hsa-miR-330-5p | TAL1 | 1 | 1 | 1 | 1 | 4 |
| hsa-miR-330-5p | CNTN2 | 1 | 1 | 1 | 1 | 4 |
| hsa-miR-330-5p | TBX2 | 1 | 1 | 1 | 1 | 4 |
| hsa-miR-330-5p | TBX15 | 1 | 1 | 1 | 1 | 4 |
| hsa-miR-330-5p | TCF4 | 1 | 1 | 1 | 1 | 4 |
| hsa-miR-330-5p | C2orf3 | 1 | 1 | 1 | 1 | 4 |
| hsa-miR-330-5p | TCF21 | 1 | 1 | 1 | 1 | 4 |
| hsa-miR-330-5p | TEAD1 | 1 | 1 | 1 | 1 | 4 |
| hsa-miR-330-5p | TEC | 1 | 1 | 1 | 1 | 4 |
| hsa-miR-330-5p | TEF | 1 | 1 | 1 | 1 | 4 |
| hsa-miR-330-5p | TEGT | 1 | 1 | 1 | 1 | 4 |
| hsa-miR-330-5p | TEP1 | 1 | 1 | 1 | 1 | 4 |
| hsa-miR-330-5p | TFAP4 | 1 | 1 | 1 | 1 | 4 |
| hsa-miR-330-5p | TFF1 | 1 | 1 | 1 | 1 | 4 |
| hsa-miR-330-5p | TGFA | 1 | 1 | 1 | 1 | 4 |
| hsa-miR-330-5p | TGFBI | 1 | 1 | 1 | 1 | 4 |
| hsa-miR-330-5p | TGM4 | 1 | 1 | 1 | 1 | 4 |
| hsa-miR-330-5p | TGM3 | 1 | 1 | 1 | 1 | 4 |
| hsa-miR-330-5p | THBS3 | 1 | 1 | 1 | 1 | 4 |
| hsa-miR-330-5p | THY1 | 1 | 1 | 1 | 1 | 4 |
| hsa-miR-330-5p | TIMP2 | 1 | 1 | 1 | 1 | 4 |
| hsa-miR-330-5p | TK2 | 1 | 1 | 1 | 1 | 4 |
| hsa-miR-330-5p | TLOC1 | 1 | 1 | 1 | 1 | 4 |
| hsa-miR-330-5p | TMF1 | 1 | 1 | 1 | 1 | 4 |
| hsa-miR-330-5p | TNFAIP1 | 1 | 1 | 1 | 1 | 4 |
| hsa-miR-330-5p | TNFAIP2 | 1 | 1 | 1 | 1 | 4 |
| hsa-miR-330-5p | TNFAIP3 | 1 | 1 | 1 | 1 | 4 |
| hsa-miR-330-5p | TNFRSF1B | 1 | 1 | 1 | 1 | 4 |
| hsa-miR-330-5p | TNNI1 | 1 | 1 | 1 | 1 | 4 |
| hsa-miR-330-5p | TNP1 | 1 | 1 | 1 | 1 | 4 |
| hsa-miR-330-5p | TNR | 1 | 1 | 1 | 1 | 4 |
| hsa-miR-330-5p | TP73 | 1 | 1 | 1 | 1 | 4 |
| hsa-miR-330-5p | TPMT | 1 | 1 | 1 | 1 | 4 |
| hsa-miR-330-5p | NR2C2 | 1 | 1 | 1 | 1 | 4 |
| hsa-miR-330-5p | TRH | 1 | 1 | 1 | 1 | 4 |
| hsa-miR-330-5p | TSHR | 1 | 1 | 1 | 1 | 4 |
| hsa-miR-330-5p | CAPN5 | 1 | 1 | 1 | 1 | 4 |
| hsa-miR-330-5p | TUB | 1 | 1 | 1 | 1 | 4 |
| hsa-miR-330-5p | TULP1 | 1 | 1 | 1 | 1 | 4 |
| hsa-miR-330-5p | TWIST1 | 1 | 1 | 1 | 1 | 4 |
| hsa-miR-330-5p | C7 | 1 | 1 | 1 | 1 | 4 |
| hsa-miR-330-5p | TYRO3 | 1 | 1 | 1 | 1 | 4 |
| hsa-miR-330-5p | UBE2G1 | 1 | 1 | 1 | 1 | 4 |
| hsa-miR-330-5p | UBE2H | 1 | 1 | 1 | 1 | 4 |
| hsa-miR-330-5p | C8G | 1 | 1 | 1 | 1 | 4 |
| hsa-miR-330-5p | UPK1B | 1 | 1 | 1 | 1 | 4 |
| hsa-miR-330-5p | UCN | 1 | 1 | 1 | 1 | 4 |
| hsa-miR-330-5p | UCP3 | 1 | 1 | 1 | 1 | 4 |
| hsa-miR-330-5p | COL14A1 | 1 | 1 | 1 | 1 | 4 |
| hsa-miR-330-5p | UQCRC2 | 1 | 1 | 1 | 1 | 4 |
| hsa-miR-330-5p | USF1 | 1 | 1 | 1 | 1 | 4 |
| hsa-miR-330-5p | USH2A | 1 | 1 | 1 | 1 | 4 |
| hsa-miR-330-5p | UVRAG | 1 | 1 | 1 | 1 | 4 |
| hsa-miR-330-5p | VCL | 1 | 1 | 1 | 1 | 4 |
| hsa-miR-330-5p | VGF | 1 | 1 | 1 | 1 | 4 |
| hsa-miR-330-5p | VIPR2 | 1 | 1 | 1 | 1 | 4 |
| hsa-miR-330-5p | CLIP2 | 1 | 1 | 1 | 1 | 4 |
| hsa-miR-330-5p | WHSC1 | 1 | 1 | 1 | 1 | 4 |
| hsa-miR-330-5p | WNT5A | 1 | 1 | 1 | 1 | 4 |
| hsa-miR-330-5p | XDH | 1 | 1 | 1 | 1 | 4 |
| hsa-miR-330-5p | ZFX | 1 | 1 | 1 | 1 | 4 |
| hsa-miR-330-5p | ZNF3 | 1 | 1 | 1 | 1 | 4 |
| hsa-miR-330-5p | ZNF12 | 1 | 1 | 1 | 1 | 4 |
| hsa-miR-330-5p | ZNF20 | 1 | 1 | 1 | 1 | 4 |
| hsa-miR-330-5p | ZNF43 | 1 | 1 | 1 | 1 | 4 |
| hsa-miR-330-5p | ZNF70 | 1 | 1 | 1 | 1 | 4 |
| hsa-miR-330-5p | ZNF79 | 1 | 1 | 1 | 1 | 4 |
| hsa-miR-330-5p | ZNF80 | 1 | 1 | 1 | 1 | 4 |
| hsa-miR-330-5p | ZNF135 | 1 | 1 | 1 | 1 | 4 |
| hsa-miR-330-5p | ZNF140 | 1 | 1 | 1 | 1 | 4 |
| hsa-miR-330-5p | TRIM25 | 1 | 1 | 1 | 1 | 4 |
| hsa-miR-330-5p | ZNF185 | 1 | 1 | 1 | 1 | 4 |
| hsa-miR-330-5p | ZNF208 | 1 | 1 | 1 | 1 | 4 |
| hsa-miR-330-5p | CACNA1E | 1 | 1 | 1 | 1 | 4 |
| hsa-miR-330-5p | SLC30A4 | 1 | 1 | 1 | 1 | 4 |
| hsa-miR-330-5p | PRDM2 | 1 | 1 | 1 | 1 | 4 |
| hsa-miR-330-5p | LAPTM5 | 1 | 1 | 1 | 1 | 4 |
| hsa-miR-330-5p | CACNB1 | 1 | 1 | 1 | 1 | 4 |
| hsa-miR-330-5p | CACNB3 | 1 | 1 | 1 | 1 | 4 |
| hsa-miR-330-5p | FZD5 | 1 | 1 | 1 | 1 | 4 |
| hsa-miR-330-5p | CACNG1 | 1 | 1 | 1 | 1 | 4 |
| hsa-miR-330-5p | BRPF1 | 1 | 1 | 1 | 1 | 4 |
| hsa-miR-330-5p | CUEDC2 | 1 | 1 | 1 | 1 | 4 |
| hsa-miR-330-5p | DDA1 | 1 | 1 | 1 | 1 | 4 |
| hsa-miR-330-5p | C17orf39 | 1 | 1 | 1 | 1 | 4 |
| hsa-miR-330-5p | KCTD15 | 1 | 1 | 1 | 1 | 4 |
| hsa-miR-330-5p | DSCC1 | 1 | 1 | 1 | 1 | 4 |
| hsa-miR-330-5p | C19orf42 | 1 | 1 | 1 | 1 | 4 |
| hsa-miR-330-5p | CARD14 | 1 | 1 | 1 | 1 | 4 |
| hsa-miR-330-5p | TRIM48 | 1 | 1 | 1 | 1 | 4 |
| hsa-miR-330-5p | JOSD3 | 1 | 1 | 1 | 1 | 4 |
| hsa-miR-330-5p | MBOAT7 | 1 | 1 | 1 | 1 | 4 |
| hsa-miR-330-5p | PLEKHF1 | 1 | 1 | 1 | 1 | 4 |
| hsa-miR-330-5p | ATAD4 | 1 | 1 | 1 | 1 | 4 |
| hsa-miR-330-5p | BRCC3 | 1 | 1 | 1 | 1 | 4 |
| hsa-miR-330-5p | TMEM43 | 1 | 1 | 1 | 1 | 4 |
| hsa-miR-330-5p | NSBP1 | 1 | 1 | 1 | 1 | 4 |
| hsa-miR-330-5p | EPS8L3 | 1 | 1 | 1 | 1 | 4 |
| hsa-miR-330-5p | C10orf76 | 1 | 1 | 1 | 1 | 4 |
| hsa-miR-330-5p | RNF219 | 1 | 1 | 1 | 1 | 4 |
| hsa-miR-330-5p | LASS4 | 1 | 1 | 1 | 1 | 4 |
| hsa-miR-330-5p | RIC3 | 1 | 1 | 1 | 1 | 4 |
| hsa-miR-330-5p | TNFAIP8L2 | 1 | 1 | 1 | 1 | 4 |
| hsa-miR-330-5p | ARMC7 | 1 | 1 | 1 | 1 | 4 |
| hsa-miR-330-5p | ARSJ | 1 | 1 | 1 | 1 | 4 |
| hsa-miR-330-5p | C16orf57 | 1 | 1 | 1 | 1 | 4 |
| hsa-miR-330-5p | HECTD3 | 1 | 1 | 1 | 1 | 4 |
| hsa-miR-330-5p | OGFOD2 | 1 | 1 | 1 | 1 | 4 |
| hsa-miR-330-5p | LRRK1 | 1 | 1 | 1 | 1 | 4 |
| hsa-miR-330-5p | WDR59 | 1 | 1 | 1 | 1 | 4 |
| hsa-miR-330-5p | LIN28 | 1 | 1 | 1 | 1 | 4 |
| hsa-miR-330-5p | ZBBX | 1 | 1 | 1 | 1 | 4 |
| hsa-miR-330-5p | ZNF419 | 1 | 1 | 1 | 1 | 4 |
| hsa-miR-330-5p | MAFK | 1 | 1 | 1 | 1 | 4 |
| hsa-miR-330-5p | C1orf115 | 1 | 1 | 1 | 1 | 4 |
| hsa-miR-330-5p | NAT11 | 1 | 1 | 1 | 1 | 4 |
| hsa-miR-330-5p | SGK269 | 1 | 1 | 1 | 1 | 4 |
| hsa-miR-330-5p | ZDHHC11 | 1 | 1 | 1 | 1 | 4 |
| hsa-miR-330-5p | TMEM180 | 1 | 1 | 1 | 1 | 4 |
| hsa-miR-330-5p | THSD4 | 1 | 1 | 1 | 1 | 4 |
| hsa-miR-330-5p | ZNF212 | 1 | 1 | 1 | 1 | 4 |
| hsa-miR-330-5p | HDAC11 | 1 | 1 | 1 | 1 | 4 |
| hsa-miR-330-5p | RIN3 | 1 | 1 | 1 | 1 | 4 |
| hsa-miR-330-5p | CYBRD1 | 1 | 1 | 1 | 1 | 4 |
| hsa-miR-330-5p | CNTD2 | 1 | 1 | 1 | 1 | 4 |
| hsa-miR-330-5p | ZNF696 | 1 | 1 | 1 | 1 | 4 |
| hsa-miR-330-5p | C20orf39 | 1 | 1 | 1 | 1 | 4 |
| hsa-miR-330-5p | FRMD1 | 1 | 1 | 1 | 1 | 4 |
| hsa-miR-330-5p | PSCA | 1 | 1 | 1 | 1 | 4 |
| hsa-miR-330-5p | DOCK5 | 1 | 1 | 1 | 1 | 4 |
| hsa-miR-330-5p | PHC3 | 1 | 1 | 1 | 1 | 4 |
| hsa-miR-330-5p | SLC24A6 | 1 | 1 | 1 | 1 | 4 |
| hsa-miR-330-5p | FAM130A2 | 1 | 1 | 1 | 1 | 4 |
| hsa-miR-330-5p | ATF7IP2 | 1 | 1 | 1 | 1 | 4 |
| hsa-miR-330-5p | C2orf37 | 1 | 1 | 1 | 1 | 4 |
| hsa-miR-330-5p | ZFP2 | 1 | 1 | 1 | 1 | 4 |
| hsa-miR-330-5p | LRRC8E | 1 | 1 | 1 | 1 | 4 |
| hsa-miR-330-5p | ZC3H12A | 1 | 1 | 1 | 1 | 4 |
| hsa-miR-330-5p | MYCT1 | 1 | 1 | 1 | 1 | 4 |
| hsa-miR-330-5p | C16orf59 | 1 | 1 | 1 | 1 | 4 |
| hsa-miR-330-5p | OPA3 | 1 | 1 | 1 | 1 | 4 |
| hsa-miR-330-5p | RAB11FIP1 | 1 | 1 | 1 | 1 | 4 |
| hsa-miR-330-5p | CEP63 | 1 | 1 | 1 | 1 | 4 |
| hsa-miR-330-5p | CCDC6 | 1 | 1 | 1 | 1 | 4 |
| hsa-miR-330-5p | C2orf44 | 1 | 1 | 1 | 1 | 4 |
| hsa-miR-330-5p | SP6 | 1 | 1 | 1 | 1 | 4 |
| hsa-miR-330-5p | ULBP1 | 1 | 1 | 1 | 1 | 4 |
| hsa-miR-330-5p | ADAM33 | 1 | 1 | 1 | 1 | 4 |
| hsa-miR-330-5p | WDR82 | 1 | 1 | 1 | 1 | 4 |
| hsa-miR-330-5p | CD276 | 1 | 1 | 1 | 1 | 4 |
| hsa-miR-330-5p | AKNA | 1 | 1 | 1 | 1 | 4 |
| hsa-miR-330-5p | SNIP | 1 | 1 | 1 | 1 | 4 |
| hsa-miR-330-5p | TTYH3 | 1 | 1 | 1 | 1 | 4 |
| hsa-miR-330-5p | FGF23 | 1 | 1 | 1 | 1 | 4 |
| hsa-miR-330-5p | ASXL3 | 1 | 1 | 1 | 1 | 4 |
| hsa-miR-330-5p | JHDM1D | 1 | 1 | 1 | 1 | 4 |
| hsa-miR-330-5p | SETD7 | 1 | 1 | 1 | 1 | 4 |
| hsa-miR-330-5p | SLC2A10 | 1 | 1 | 1 | 1 | 4 |
| hsa-miR-330-5p | SLC25A32 | 1 | 1 | 1 | 1 | 4 |
| hsa-miR-330-5p | CALR | 1 | 1 | 1 | 1 | 4 |
| hsa-miR-330-5p | GPR68 | 1 | 1 | 1 | 1 | 4 |
| hsa-miR-330-5p | ST8SIA2 | 1 | 1 | 1 | 1 | 4 |
| hsa-miR-330-5p | SLC7A5 | 1 | 1 | 1 | 1 | 4 |
| hsa-miR-330-5p | OR2C3 | 1 | 1 | 1 | 1 | 4 |
| hsa-miR-330-5p | PTDSS2 | 1 | 1 | 1 | 1 | 4 |
| hsa-miR-330-5p | SYNC1 | 1 | 1 | 1 | 1 | 4 |
| hsa-miR-330-5p | CAMK2A | 1 | 1 | 1 | 1 | 4 |
| hsa-miR-330-5p | STMN4 | 1 | 1 | 1 | 1 | 4 |
| hsa-miR-330-5p | C1orf21 | 1 | 1 | 1 | 1 | 4 |
| hsa-miR-330-5p | NDEL1 | 1 | 1 | 1 | 1 | 4 |
| hsa-miR-330-5p | TRIM8 | 1 | 1 | 1 | 1 | 4 |
| hsa-miR-330-5p | URM1 | 1 | 1 | 1 | 1 | 4 |
| hsa-miR-330-5p | SNX27 | 1 | 1 | 1 | 1 | 4 |
| hsa-miR-330-5p | ANP32E | 1 | 1 | 1 | 1 | 4 |
| hsa-miR-330-5p | UNC93B1 | 1 | 1 | 1 | 1 | 4 |
| hsa-miR-330-5p | PPP1R14C | 1 | 1 | 1 | 1 | 4 |
| hsa-miR-330-5p | ELL | 1 | 1 | 1 | 1 | 4 |
| hsa-miR-330-5p | VANGL1 | 1 | 1 | 1 | 1 | 4 |
| hsa-miR-330-5p | ARPC5L | 1 | 1 | 1 | 1 | 4 |
| hsa-miR-330-5p | RAB1B | 1 | 1 | 1 | 1 | 4 |
| hsa-miR-330-5p | CABLES2 | 1 | 1 | 1 | 1 | 4 |
| hsa-miR-330-5p | NCOA3 | 1 | 1 | 1 | 1 | 4 |
| hsa-miR-330-5p | DGCR14 | 1 | 1 | 1 | 1 | 4 |
| hsa-miR-330-5p | USP11 | 1 | 1 | 1 | 1 | 4 |
| hsa-miR-330-5p | RBM10 | 1 | 1 | 1 | 1 | 4 |
| hsa-miR-330-5p | UBL4A | 1 | 1 | 1 | 1 | 4 |
| hsa-miR-330-5p | CAPS | 1 | 1 | 1 | 1 | 4 |
| hsa-miR-330-5p | USP9Y | 1 | 1 | 1 | 1 | 4 |
| hsa-miR-330-5p | FZD4 | 1 | 1 | 1 | 1 | 4 |
| hsa-miR-330-5p | ELOVL3 | 1 | 1 | 1 | 1 | 4 |
| hsa-miR-330-5p | TCF7L1 | 1 | 1 | 1 | 1 | 4 |
| hsa-miR-330-5p | RAB33B | 1 | 1 | 1 | 1 | 4 |
| hsa-miR-330-5p | PLVAP | 1 | 1 | 1 | 1 | 4 |
| hsa-miR-330-5p | RTBDN | 1 | 1 | 1 | 1 | 4 |
| hsa-miR-330-5p | RASSF5 | 1 | 1 | 1 | 1 | 4 |
| hsa-miR-330-5p | SOX7 | 1 | 1 | 1 | 1 | 4 |
| hsa-miR-330-5p | ZMIZ2 | 1 | 1 | 1 | 1 | 4 |
| hsa-miR-330-5p | FAM107B | 1 | 1 | 1 | 1 | 4 |
| hsa-miR-330-5p | CCDC3 | 1 | 1 | 1 | 1 | 4 |
| hsa-miR-330-5p | C8orf13 | 1 | 1 | 1 | 1 | 4 |
| hsa-miR-330-5p | DYNLRB1 | 1 | 1 | 1 | 1 | 4 |
| hsa-miR-330-5p | SESN2 | 1 | 1 | 1 | 1 | 4 |
| hsa-miR-330-5p | CD99L2 | 1 | 1 | 1 | 1 | 4 |
| hsa-miR-330-5p | HSDL1 | 1 | 1 | 1 | 1 | 4 |
| hsa-miR-330-5p | JAM3 | 1 | 1 | 1 | 1 | 4 |
| hsa-miR-330-5p | CRISPLD2 | 1 | 1 | 1 | 1 | 4 |
| hsa-miR-330-5p | SLC25A18 | 1 | 1 | 1 | 1 | 4 |
| hsa-miR-330-5p | GRWD1 | 1 | 1 | 1 | 1 | 4 |
| hsa-miR-330-5p | LONP2 | 1 | 1 | 1 | 1 | 4 |
| hsa-miR-330-5p | KLF16 | 1 | 1 | 1 | 1 | 4 |
| hsa-miR-330-5p | STK40 | 1 | 1 | 1 | 1 | 4 |
| hsa-miR-330-5p | TMEM133 | 1 | 1 | 1 | 1 | 4 |
| hsa-miR-330-5p | RASSF4 | 1 | 1 | 1 | 1 | 4 |
| hsa-miR-330-5p | EIF2A | 1 | 1 | 1 | 1 | 4 |
| hsa-miR-330-5p | TSSK1B | 1 | 1 | 1 | 1 | 4 |
| hsa-miR-330-5p | PIP4K2B | 1 | 1 | 1 | 1 | 4 |
| hsa-miR-330-5p | TMPRSS13 | 1 | 1 | 1 | 1 | 4 |
| hsa-miR-330-5p | HDHD2 | 1 | 1 | 1 | 1 | 4 |
| hsa-miR-330-5p | C1orf160 | 1 | 1 | 1 | 1 | 4 |
| hsa-miR-330-5p | TAGLN2 | 1 | 1 | 1 | 1 | 4 |
| hsa-miR-330-5p | ARID5B | 1 | 1 | 1 | 1 | 4 |
| hsa-miR-330-5p | C19orf44 | 1 | 1 | 1 | 1 | 4 |
| hsa-miR-330-5p | ANTXR1 | 1 | 1 | 1 | 1 | 4 |
| hsa-miR-330-5p | RBED1 | 1 | 1 | 1 | 1 | 4 |
| hsa-miR-330-5p | ZDHHC18 | 1 | 1 | 1 | 1 | 4 |
| hsa-miR-330-5p | LDOC1L | 1 | 1 | 1 | 1 | 4 |
| hsa-miR-330-5p | COQ5 | 1 | 1 | 1 | 1 | 4 |
| hsa-miR-330-5p | STK24 | 1 | 1 | 1 | 1 | 4 |
| hsa-miR-330-5p | TMEM175 | 1 | 1 | 1 | 1 | 4 |
| hsa-miR-330-5p | ING5 | 1 | 1 | 1 | 1 | 4 |
| hsa-miR-330-5p | CASP10 | 1 | 1 | 1 | 1 | 4 |
| hsa-miR-330-5p | PCGF5 | 1 | 1 | 1 | 1 | 4 |
| hsa-miR-330-5p | ELOF1 | 1 | 1 | 1 | 1 | 4 |
| hsa-miR-330-5p | ARFGAP2 | 1 | 1 | 1 | 1 | 4 |
| hsa-miR-330-5p | RASAL1 | 1 | 1 | 1 | 1 | 4 |
| hsa-miR-330-5p | PROK1 | 1 | 1 | 1 | 1 | 4 |
| hsa-miR-330-5p | KIAA1826 | 1 | 1 | 1 | 1 | 4 |
| hsa-miR-330-5p | MAML2 | 1 | 1 | 1 | 1 | 4 |
| hsa-miR-330-5p | DYRK2 | 1 | 1 | 1 | 1 | 4 |
| hsa-miR-330-5p | LCOR | 1 | 1 | 1 | 1 | 4 |
| hsa-miR-330-5p | KIAA1787 | 1 | 1 | 1 | 1 | 4 |
| hsa-miR-330-5p | FBN3 | 1 | 1 | 1 | 1 | 4 |
| hsa-miR-330-5p | DOC2A | 1 | 1 | 1 | 1 | 4 |
| hsa-miR-330-5p | ACRBP | 1 | 1 | 1 | 1 | 4 |
| hsa-miR-330-5p | KIAA1853 | 1 | 1 | 1 | 1 | 4 |
| hsa-miR-330-5p | ATRN | 1 | 1 | 1 | 1 | 4 |
| hsa-miR-330-5p | MAP1LC3A | 1 | 1 | 1 | 1 | 4 |
| hsa-miR-330-5p | CASR | 1 | 1 | 1 | 1 | 4 |
| hsa-miR-330-5p | KLF11 | 1 | 1 | 1 | 1 | 4 |
| hsa-miR-330-5p | ZNF594 | 1 | 1 | 1 | 1 | 4 |
| hsa-miR-330-5p | FNDC1 | 1 | 1 | 1 | 1 | 4 |
| hsa-miR-330-5p | N-PAC | 1 | 1 | 1 | 1 | 4 |
| hsa-miR-330-5p | RNASE7 | 1 | 1 | 1 | 1 | 4 |
| hsa-miR-330-5p | USP32 | 1 | 1 | 1 | 1 | 4 |
| hsa-miR-330-5p | FBXL10 | 1 | 1 | 1 | 1 | 4 |
| hsa-miR-330-5p | FAM71F1 | 1 | 1 | 1 | 1 | 4 |
| hsa-miR-330-5p | GPT2 | 1 | 1 | 1 | 1 | 4 |
| hsa-miR-330-5p | CBX2 | 1 | 1 | 1 | 1 | 4 |
| hsa-miR-330-5p | C1orf90 | 1 | 1 | 1 | 1 | 4 |
| hsa-miR-330-5p | ZNF577 | 1 | 1 | 1 | 1 | 4 |
| hsa-miR-330-5p | ZNF607 | 1 | 1 | 1 | 1 | 4 |
| hsa-miR-330-5p | SUV420H2 | 1 | 1 | 1 | 1 | 4 |
| hsa-miR-330-5p | PPAPDC3 | 1 | 1 | 1 | 1 | 4 |
| hsa-miR-330-5p | LMNB2 | 1 | 1 | 1 | 1 | 4 |
| hsa-miR-330-5p | FCRLA | 1 | 1 | 1 | 1 | 4 |
| hsa-miR-330-5p | RAX2 | 1 | 1 | 1 | 1 | 4 |
| hsa-miR-330-5p | PHF5A | 1 | 1 | 1 | 1 | 4 |
| hsa-miR-330-5p | TMEM25 | 1 | 1 | 1 | 1 | 4 |
| hsa-miR-330-5p | ZC3H10 | 1 | 1 | 1 | 1 | 4 |
| hsa-miR-330-5p | FAM73B | 1 | 1 | 1 | 1 | 4 |
| hsa-miR-330-5p | ZNF341 | 1 | 1 | 1 | 1 | 4 |
| hsa-miR-330-5p | C9orf3 | 1 | 1 | 1 | 1 | 4 |
| hsa-miR-330-5p | ZNF382 | 1 | 1 | 1 | 1 | 4 |
| hsa-miR-330-5p | SLC35B4 | 1 | 1 | 1 | 1 | 4 |
| hsa-miR-330-5p | FAM104A | 1 | 1 | 1 | 1 | 4 |
| hsa-miR-330-5p | FIBCD1 | 1 | 1 | 1 | 1 | 4 |
| hsa-miR-330-5p | PRPF38A | 1 | 1 | 1 | 1 | 4 |
| hsa-miR-330-5p | UBASH3B | 1 | 1 | 1 | 1 | 4 |
| hsa-miR-330-5p | ARHGAP19 | 1 | 1 | 1 | 1 | 4 |
| hsa-miR-330-5p | PPFIA1 | 1 | 1 | 1 | 1 | 4 |
| hsa-miR-330-5p | RERG | 1 | 1 | 1 | 1 | 4 |
| hsa-miR-330-5p | C18orf45 | 1 | 1 | 1 | 1 | 4 |
| hsa-miR-330-5p | TMEM60 | 1 | 1 | 1 | 1 | 4 |
| hsa-miR-330-5p | PIK3R3 | 1 | 1 | 1 | 1 | 4 |
| hsa-miR-330-5p | NIPSNAP1 | 1 | 1 | 1 | 1 | 4 |
| hsa-miR-330-5p | ZCCHC3 | 1 | 1 | 1 | 1 | 4 |
| hsa-miR-330-5p | EAF1 | 1 | 1 | 1 | 1 | 4 |
| hsa-miR-330-5p | NKD1 | 1 | 1 | 1 | 1 | 4 |
| hsa-miR-330-5p | ZIC5 | 1 | 1 | 1 | 1 | 4 |
| hsa-miR-330-5p | KIAA1754 | 1 | 1 | 1 | 1 | 4 |
| hsa-miR-330-5p | UNK | 1 | 1 | 1 | 1 | 4 |
| hsa-miR-330-5p | ZC3H12C | 1 | 1 | 1 | 1 | 4 |
| hsa-miR-330-5p | SSH2 | 1 | 1 | 1 | 1 | 4 |
| hsa-miR-330-5p | TSLP | 1 | 1 | 1 | 1 | 4 |
| hsa-miR-330-5p | BHLHB2 | 1 | 1 | 1 | 1 | 4 |
| hsa-miR-330-5p | KMO | 1 | 1 | 1 | 1 | 4 |
| hsa-miR-330-5p | PDXK | 1 | 1 | 1 | 1 | 4 |
| hsa-miR-330-5p | RRP1 | 1 | 1 | 1 | 1 | 4 |
| hsa-miR-330-5p | MKNK1 | 1 | 1 | 1 | 1 | 4 |
| hsa-miR-330-5p | KHSRP | 1 | 1 | 1 | 1 | 4 |
| hsa-miR-330-5p | PDLIM4 | 1 | 1 | 1 | 1 | 4 |
| hsa-miR-330-5p | STC2 | 1 | 1 | 1 | 1 | 4 |
| hsa-miR-330-5p | CBFA2T3 | 1 | 1 | 1 | 1 | 4 |
| hsa-miR-330-5p | KCNK5 | 1 | 1 | 1 | 1 | 4 |
| hsa-miR-330-5p | CBFB | 1 | 1 | 1 | 1 | 4 |
| hsa-miR-330-5p | NUMB | 1 | 1 | 1 | 1 | 4 |
| hsa-miR-330-5p | PDE5A | 1 | 1 | 1 | 1 | 4 |
| hsa-miR-330-5p | TNKS | 1 | 1 | 1 | 1 | 4 |
| hsa-miR-330-5p | ALDH4A1 | 1 | 1 | 1 | 1 | 4 |
| hsa-miR-330-5p | EIF3I | 1 | 1 | 1 | 1 | 4 |
| hsa-miR-330-5p | CBL | 1 | 1 | 1 | 1 | 4 |
| hsa-miR-330-5p | SLC4A4 | 1 | 1 | 1 | 1 | 4 |
| hsa-miR-330-5p | B3GALT1 | 1 | 1 | 1 | 1 | 4 |
| hsa-miR-330-5p | TNK1 | 1 | 1 | 1 | 1 | 4 |
| hsa-miR-330-5p | MBTPS1 | 1 | 1 | 1 | 1 | 4 |
| hsa-miR-330-5p | ADAM19 | 1 | 1 | 1 | 1 | 4 |
| hsa-miR-330-5p | RNGTT | 1 | 1 | 1 | 1 | 4 |
| hsa-miR-330-5p | RIPK1 | 1 | 1 | 1 | 1 | 4 |
| hsa-miR-330-5p | HRK | 1 | 1 | 1 | 1 | 4 |
| hsa-miR-330-5p | TNFRSF14 | 1 | 1 | 1 | 1 | 4 |
| hsa-miR-330-5p | RIPK2 | 1 | 1 | 1 | 1 | 4 |
| hsa-miR-330-5p | MTMR1 | 1 | 1 | 1 | 1 | 4 |
| hsa-miR-330-5p | RIOK3 | 1 | 1 | 1 | 1 | 4 |
| hsa-miR-330-5p | TNFRSF11A | 1 | 1 | 1 | 1 | 4 |
| hsa-miR-330-5p | WDR22 | 1 | 1 | 1 | 1 | 4 |
| hsa-miR-330-5p | FGF18 | 1 | 1 | 1 | 1 | 4 |
| hsa-miR-330-5p | NRP1 | 1 | 1 | 1 | 1 | 4 |
| hsa-miR-330-5p | SYNGAP1 | 1 | 1 | 1 | 1 | 4 |
| hsa-miR-330-5p | WISP1 | 1 | 1 | 1 | 1 | 4 |
| hsa-miR-330-5p | CCK | 1 | 1 | 1 | 1 | 4 |
| hsa-miR-330-5p | CDK5R1 | 1 | 1 | 1 | 1 | 4 |
| hsa-miR-330-5p | VNN1 | 1 | 1 | 1 | 1 | 4 |
| hsa-miR-330-5p | MTMR3 | 1 | 1 | 1 | 1 | 4 |
| hsa-miR-330-5p | GYG2 | 1 | 1 | 1 | 1 | 4 |
| hsa-miR-330-5p | P11 | 1 | 1 | 1 | 1 | 4 |
| hsa-miR-330-5p | BSN | 1 | 1 | 1 | 1 | 4 |
| hsa-miR-330-5p | CCND3 | 1 | 1 | 1 | 1 | 4 |
| hsa-miR-330-5p | NAV3 | 1 | 1 | 1 | 1 | 4 |
| hsa-miR-330-5p | NAV1 | 1 | 1 | 1 | 1 | 4 |
| hsa-miR-330-5p | NAV2 | 1 | 1 | 1 | 1 | 4 |
| hsa-miR-330-5p | FAM125B | 1 | 1 | 1 | 1 | 4 |
| hsa-miR-330-5p | SLAMF9 | 1 | 1 | 1 | 1 | 4 |
| hsa-miR-330-5p | CCNF | 1 | 1 | 1 | 1 | 4 |
| hsa-miR-330-5p | LIMD1 | 1 | 1 | 1 | 1 | 4 |
| hsa-miR-330-5p | CDKL2 | 1 | 1 | 1 | 1 | 4 |
| hsa-miR-330-5p | MIDN | 1 | 1 | 1 | 1 | 4 |
| hsa-miR-330-5p | C14orf152 | 1 | 1 | 1 | 1 | 4 |
| hsa-miR-330-5p | TAF1B | 1 | 1 | 1 | 1 | 4 |
| hsa-miR-330-5p | MPZL1 | 1 | 1 | 1 | 1 | 4 |
| hsa-miR-330-5p | MAP3K14 | 1 | 1 | 1 | 1 | 4 |
| hsa-miR-330-5p | UNC5A | 1 | 1 | 1 | 1 | 4 |
| hsa-miR-330-5p | RNF8 | 1 | 1 | 1 | 1 | 4 |
| hsa-miR-330-5p | FAM105B | 1 | 1 | 1 | 1 | 4 |
| hsa-miR-330-5p | CEACAM21 | 1 | 1 | 1 | 1 | 4 |
| hsa-miR-330-5p | C3orf25 | 1 | 1 | 1 | 1 | 4 |
| hsa-miR-330-5p | PSTPIP2 | 1 | 1 | 1 | 1 | 4 |
| hsa-miR-330-5p | GPRC5A | 1 | 1 | 1 | 1 | 4 |
| hsa-miR-330-5p | SHF | 1 | 1 | 1 | 1 | 4 |
| hsa-miR-330-5p | C1orf201 | 1 | 1 | 1 | 1 | 4 |
| hsa-miR-330-5p | CLDN12 | 1 | 1 | 1 | 1 | 4 |
| hsa-miR-330-5p | CLDN8 | 1 | 1 | 1 | 1 | 4 |
| hsa-miR-330-5p | DIRAS3 | 1 | 1 | 1 | 1 | 4 |
| hsa-miR-330-5p | PYGO2 | 1 | 1 | 1 | 1 | 4 |
| hsa-miR-330-5p | ANGEL2 | 1 | 1 | 1 | 1 | 4 |
| hsa-miR-330-5p | HN1L | 1 | 1 | 1 | 1 | 4 |
| hsa-miR-330-5p | DNAJA3 | 1 | 1 | 1 | 1 | 4 |
| hsa-miR-330-5p | TBX19 | 1 | 1 | 1 | 1 | 4 |
| hsa-miR-330-5p | ACVR1B | 1 | 1 | 1 | 1 | 4 |
| hsa-miR-330-5p | FMNL3 | 1 | 1 | 1 | 1 | 4 |
| hsa-miR-330-5p | CD1C | 1 | 1 | 1 | 1 | 4 |
| hsa-miR-330-5p | ATP6V0D1 | 1 | 1 | 1 | 1 | 4 |
| hsa-miR-330-5p | INA | 1 | 1 | 1 | 1 | 4 |
| hsa-miR-330-5p | P2RX6 | 1 | 1 | 1 | 1 | 4 |
| hsa-miR-330-5p | CD1E | 1 | 1 | 1 | 1 | 4 |
| hsa-miR-330-5p | SYNGR3 | 1 | 1 | 1 | 1 | 4 |
| hsa-miR-330-5p | SYNGR2 | 1 | 1 | 1 | 1 | 4 |
| hsa-miR-330-5p | NEURL | 1 | 1 | 1 | 1 | 4 |
| hsa-miR-330-5p | COL23A1 | 1 | 1 | 1 | 1 | 4 |
| hsa-miR-330-5p | PCSK7 | 1 | 1 | 1 | 1 | 4 |
| hsa-miR-330-5p | RASL10B | 1 | 1 | 1 | 1 | 4 |
| hsa-miR-330-5p | KIAA1919 | 1 | 1 | 1 | 1 | 4 |
| hsa-miR-330-5p | CHMP7 | 1 | 1 | 1 | 1 | 4 |
| hsa-miR-330-5p | DDX21 | 1 | 1 | 1 | 1 | 4 |
| hsa-miR-330-5p | CD247 | 1 | 1 | 1 | 1 | 4 |
| hsa-miR-330-5p | ZC3HAV1L | 1 | 1 | 1 | 1 | 4 |
| hsa-miR-330-5p | ABBA-1 | 1 | 1 | 1 | 1 | 4 |
| hsa-miR-330-5p | VAPA | 1 | 1 | 1 | 1 | 4 |
| hsa-miR-330-5p | DLGAP2 | 1 | 1 | 1 | 1 | 4 |
| hsa-miR-330-5p | GLYATL1 | 1 | 1 | 1 | 1 | 4 |
| hsa-miR-330-5p | LYK5 | 1 | 1 | 1 | 1 | 4 |
| hsa-miR-330-5p | C1orf105 | 1 | 1 | 1 | 1 | 4 |
| hsa-miR-330-5p | CCDC64 | 1 | 1 | 1 | 1 | 4 |
| hsa-miR-330-5p | BZRAP1 | 1 | 1 | 1 | 1 | 4 |
| hsa-miR-330-5p | ZNF764 | 1 | 1 | 1 | 1 | 4 |
| hsa-miR-330-5p | TIMM50 | 1 | 1 | 1 | 1 | 4 |
| hsa-miR-330-5p | MAPKAPK2 | 1 | 1 | 1 | 1 | 4 |
| hsa-miR-330-5p | TIFA | 1 | 1 | 1 | 1 | 4 |
| hsa-miR-330-5p | C20orf72 | 1 | 1 | 1 | 1 | 4 |
| hsa-miR-330-5p | GPR55 | 1 | 1 | 1 | 1 | 4 |
| hsa-miR-330-5p | 9-Mar | 1 | 1 | 1 | 1 | 4 |
| hsa-miR-330-5p | ZBTB47 | 1 | 1 | 1 | 1 | 4 |
| hsa-miR-330-5p | ACVR2B | 1 | 1 | 1 | 1 | 4 |
| hsa-miR-330-5p | PIGM | 1 | 1 | 1 | 1 | 4 |
| hsa-miR-330-5p | PERLD1 | 1 | 1 | 1 | 1 | 4 |
| hsa-miR-330-5p | GTF3C4 | 1 | 1 | 1 | 1 | 4 |
| hsa-miR-330-5p | CNOT8 | 1 | 1 | 1 | 1 | 4 |
| hsa-miR-330-5p | VAMP3 | 1 | 1 | 1 | 1 | 4 |
| hsa-miR-330-5p | EFTUD2 | 1 | 1 | 1 | 1 | 4 |
| hsa-miR-330-5p | MAPK1IP1L | 1 | 1 | 1 | 1 | 4 |
| hsa-miR-330-5p | TXNL1 | 1 | 1 | 1 | 1 | 4 |
| hsa-miR-330-5p | SLC22A8 | 1 | 1 | 1 | 1 | 4 |
| hsa-miR-330-5p | NRXN1 | 1 | 1 | 1 | 1 | 4 |
| hsa-miR-330-5p | OTOF | 1 | 1 | 1 | 1 | 4 |
| hsa-miR-330-5p | ACTR8 | 1 | 1 | 1 | 1 | 4 |
| hsa-miR-330-5p | HTRA3 | 1 | 1 | 1 | 1 | 4 |
| hsa-miR-330-5p | TMPRSS11D | 1 | 1 | 1 | 1 | 4 |
| hsa-miR-330-5p | ORMDL3 | 1 | 1 | 1 | 1 | 4 |
| hsa-miR-330-5p | CRIPT | 1 | 1 | 1 | 1 | 4 |
| hsa-miR-330-5p | ZNF264 | 1 | 1 | 1 | 1 | 4 |
| hsa-miR-330-5p | OPN4 | 1 | 1 | 1 | 1 | 4 |
| hsa-miR-330-5p | TP53INP1 | 1 | 1 | 1 | 1 | 4 |
| hsa-miR-330-5p | CHST3 | 1 | 1 | 1 | 1 | 4 |
| hsa-miR-330-5p | CD34 | 1 | 1 | 1 | 1 | 4 |
| hsa-miR-330-5p | ONECUT2 | 1 | 1 | 1 | 1 | 4 |
| hsa-miR-330-5p | PSMF1 | 1 | 1 | 1 | 1 | 4 |
| hsa-miR-330-5p | KIF23 | 1 | 1 | 1 | 1 | 4 |
| hsa-miR-330-5p | SPTLC2 | 1 | 1 | 1 | 1 | 4 |
| hsa-miR-330-5p | CD38 | 1 | 1 | 1 | 1 | 4 |
| hsa-miR-330-5p | BAG5 | 1 | 1 | 1 | 1 | 4 |
| hsa-miR-330-5p | NRG2 | 1 | 1 | 1 | 1 | 4 |
| hsa-miR-330-5p | ENTPD6 | 1 | 1 | 1 | 1 | 4 |
| hsa-miR-330-5p | ENTPD3 | 1 | 1 | 1 | 1 | 4 |
| hsa-miR-330-5p | GTPBP1 | 1 | 1 | 1 | 1 | 4 |
| hsa-miR-330-5p | GABBR2 | 1 | 1 | 1 | 1 | 4 |
| hsa-miR-330-5p | CD40 | 1 | 1 | 1 | 1 | 4 |
| hsa-miR-330-5p | CD40LG | 1 | 1 | 1 | 1 | 4 |
| hsa-miR-330-5p | CD44 | 1 | 1 | 1 | 1 | 4 |
| hsa-miR-330-5p | PDIA4 | 1 | 1 | 1 | 1 | 4 |
| hsa-miR-330-5p | TRAF4 | 1 | 1 | 1 | 1 | 4 |
| hsa-miR-330-5p | CELSR1 | 1 | 1 | 1 | 1 | 4 |
| hsa-miR-330-5p | KLK4 | 1 | 1 | 1 | 1 | 4 |
| hsa-miR-330-5p | PPM1F | 1 | 1 | 1 | 1 | 4 |
| hsa-miR-330-5p | RALGPS1 | 1 | 1 | 1 | 1 | 4 |
| hsa-miR-330-5p | HS2ST1 | 1 | 1 | 1 | 1 | 4 |
| hsa-miR-330-5p | LPIN2 | 1 | 1 | 1 | 1 | 4 |
| hsa-miR-330-5p | SLC25A44 | 1 | 1 | 1 | 1 | 4 |
| hsa-miR-330-5p | EDEM1 | 1 | 1 | 1 | 1 | 4 |
| hsa-miR-330-5p | TRAM2 | 1 | 1 | 1 | 1 | 4 |
| hsa-miR-330-5p | KIAA0100 | 1 | 1 | 1 | 1 | 4 |
| hsa-miR-330-5p | KIAA0355 | 1 | 1 | 1 | 1 | 4 |
| hsa-miR-330-5p | RAB11FIP3 | 1 | 1 | 1 | 1 | 4 |
| hsa-miR-330-5p | KIAA0562 | 1 | 1 | 1 | 1 | 4 |
| hsa-miR-330-5p | CD81 | 1 | 1 | 1 | 1 | 4 |
| hsa-miR-330-5p | PCDHA9 | 1 | 1 | 1 | 1 | 4 |
| hsa-miR-330-5p | TBKBP1 | 1 | 1 | 1 | 1 | 4 |
| hsa-miR-330-5p | HDAC4 | 1 | 1 | 1 | 1 | 4 |
| hsa-miR-330-5p | KIAA0513 | 1 | 1 | 1 | 1 | 4 |
| hsa-miR-330-5p | KIAA0247 | 1 | 1 | 1 | 1 | 4 |
| hsa-miR-330-5p | TM9SF4 | 1 | 1 | 1 | 1 | 4 |
| hsa-miR-330-5p | SNX17 | 1 | 1 | 1 | 1 | 4 |
| hsa-miR-330-5p | SERTAD2 | 1 | 1 | 1 | 1 | 4 |
| hsa-miR-330-5p | MRPL19 | 1 | 1 | 1 | 1 | 4 |
| hsa-miR-330-5p | IHPK1 | 1 | 1 | 1 | 1 | 4 |
| hsa-miR-330-5p | KIAA0141 | 1 | 1 | 1 | 1 | 4 |
| hsa-miR-330-5p | KIAA0494 | 1 | 1 | 1 | 1 | 4 |
| hsa-miR-330-5p | TRIM14 | 1 | 1 | 1 | 1 | 4 |
| hsa-miR-330-5p | GINS1 | 1 | 1 | 1 | 1 | 4 |
| hsa-miR-330-5p | ZNF518A | 1 | 1 | 1 | 1 | 4 |
| hsa-miR-330-5p | TMEM24 | 1 | 1 | 1 | 1 | 4 |
| hsa-miR-330-5p | FARP2 | 1 | 1 | 1 | 1 | 4 |
| hsa-miR-330-5p | CEP350 | 1 | 1 | 1 | 1 | 4 |
| hsa-miR-330-5p | KIAA0317 | 1 | 1 | 1 | 1 | 4 |
| hsa-miR-330-5p | ZBTB39 | 1 | 1 | 1 | 1 | 4 |
| hsa-miR-330-5p | KIAA0329 | 1 | 1 | 1 | 1 | 4 |
| hsa-miR-330-5p | SRGAP3 | 1 | 1 | 1 | 1 | 4 |
| hsa-miR-330-5p | MRC2 | 1 | 1 | 1 | 1 | 4 |
| hsa-miR-330-5p | KLHL21 | 1 | 1 | 1 | 1 | 4 |
| hsa-miR-330-5p | TMCC2 | 1 | 1 | 1 | 1 | 4 |
| hsa-miR-330-5p | SEC16A | 1 | 1 | 1 | 1 | 4 |
| hsa-miR-330-5p | JOSD1 | 1 | 1 | 1 | 1 | 4 |
| hsa-miR-330-5p | ARHGAP25 | 1 | 1 | 1 | 1 | 4 |
| hsa-miR-330-5p | XYLB | 1 | 1 | 1 | 1 | 4 |
| hsa-miR-330-5p | HS3ST2 | 1 | 1 | 1 | 1 | 4 |
| hsa-miR-330-5p | USP3 | 1 | 1 | 1 | 1 | 4 |
| hsa-miR-330-5p | MED12 | 1 | 1 | 1 | 1 | 4 |
| hsa-miR-330-5p | PPP4R1 | 1 | 1 | 1 | 1 | 4 |
| hsa-miR-330-5p | CDH1 | 1 | 1 | 1 | 1 | 4 |
| hsa-miR-330-5p | DGCR2 | 1 | 1 | 1 | 1 | 4 |
| hsa-miR-330-5p | CHL1 | 1 | 1 | 1 | 1 | 4 |
| hsa-miR-330-5p | C1orf83 | 1 | 1 | 1 | 1 | 4 |
| hsa-miR-330-5p | LRRC15 | 1 | 1 | 1 | 1 | 4 |
| hsa-miR-330-5p | CSRP1 | 1 | 1 | 1 | 1 | 4 |
| hsa-miR-330-5p | DGKG | 1 | 1 | 1 | 1 | 4 |
| hsa-miR-330-5p | XIRP1 | 1 | 1 | 1 | 1 | 4 |
| hsa-miR-330-5p | DLX1 | 1 | 1 | 1 | 1 | 4 |
| hsa-miR-330-5p | MEGF6 | 1 | 1 | 1 | 1 | 4 |
| hsa-miR-330-5p | C7orf33 | 1 | 1 | 1 | 1 | 4 |
| hsa-miR-330-5p | SARM1 | 1 | 1 | 1 | 1 | 4 |
| hsa-miR-330-5p | PLCB1 | 1 | 1 | 1 | 1 | 4 |
| hsa-miR-330-5p | PHLPP | 1 | 1 | 1 | 1 | 4 |
| hsa-miR-330-5p | C3orf27 | 1 | 1 | 1 | 1 | 4 |
| hsa-miR-330-5p | SLC7A11 | 1 | 1 | 1 | 1 | 4 |
| hsa-miR-330-5p | PGDS | 1 | 1 | 1 | 1 | 4 |
| hsa-miR-330-5p | HLA-DOA | 1 | 1 | 1 | 1 | 4 |
| hsa-miR-330-5p | ZC3H12B | 1 | 1 | 1 | 1 | 4 |
| hsa-miR-330-5p | ITGA5 | 1 | 1 | 1 | 1 | 4 |
| hsa-miR-330-5p | KCNS1 | 1 | 1 | 1 | 1 | 4 |
| hsa-miR-330-5p | FLG2 | 1 | 1 | 1 | 1 | 4 |
| hsa-miR-330-5p | SMAD3 | 1 | 1 | 1 | 1 | 4 |
| hsa-miR-330-5p | NHS | 1 | 1 | 1 | 1 | 4 |
| hsa-miR-330-5p | NCKIPSD | 1 | 1 | 1 | 1 | 4 |
| hsa-miR-330-5p | PPIL1 | 1 | 1 | 1 | 1 | 4 |
| hsa-miR-330-5p | RFWD3 | 1 | 1 | 1 | 1 | 4 |
| hsa-miR-330-5p | PPP2R5B | 1 | 1 | 1 | 1 | 4 |
| hsa-miR-330-5p | DPYSL5 | 1 | 1 | 1 | 1 | 4 |
| hsa-miR-330-5p | GATAD2B | 1 | 1 | 1 | 1 | 4 |
| hsa-miR-330-5p | KLHL1 | 1 | 1 | 1 | 1 | 4 |
| hsa-miR-330-5p | RARG | 1 | 1 | 1 | 1 | 4 |
| hsa-miR-330-5p | RORB | 1 | 1 | 1 | 1 | 4 |
| hsa-miR-330-5p | PAPOLG | 1 | 1 | 1 | 1 | 4 |
| hsa-miR-330-5p | ADAM17 | 1 | 1 | 1 | 1 | 4 |
| hsa-miR-330-5p | CA12 | 1 | 1 | 1 | 1 | 4 |
| hsa-miR-330-5p | FYCO1 | 1 | 1 | 1 | 1 | 4 |
| hsa-miR-330-5p | CCNJL | 1 | 1 | 1 | 1 | 4 |
| hsa-miR-330-5p | TREML2 | 1 | 1 | 1 | 1 | 4 |
| hsa-miR-330-5p | ALDH1A2 | 1 | 1 | 1 | 1 | 4 |
| hsa-miR-330-5p | CALML4 | 1 | 1 | 1 | 1 | 4 |
| hsa-miR-330-5p | UBE4A | 1 | 1 | 1 | 1 | 4 |
| hsa-miR-330-5p | TNFRSF8 | 1 | 1 | 1 | 1 | 4 |
| hsa-miR-330-5p | CHD1L | 1 | 1 | 1 | 1 | 4 |
| hsa-miR-330-5p | FAM53B | 1 | 1 | 1 | 1 | 4 |
| hsa-miR-330-5p | N4BP1 | 1 | 1 | 1 | 1 | 4 |
| hsa-miR-330-5p | CDH3 | 1 | 1 | 1 | 1 | 4 |
| hsa-miR-330-5p | BCL2L11 | 1 | 1 | 1 | 1 | 4 |
| hsa-miR-330-5p | MBOAT5 | 1 | 1 | 1 | 1 | 4 |
| **hsa-miR-330-5p** | **SPRY2** | **1** | **1** | **1** | **1** | **4** |
| hsa-miR-330-5p | BET1 | 1 | 1 | 1 | 1 | 4 |
| hsa-miR-330-5p | SPEG | 1 | 1 | 1 | 1 | 4 |
| hsa-miR-330-5p | SF3A1 | 1 | 1 | 1 | 1 | 4 |
| hsa-miR-330-5p | B3GNT3 | 1 | 1 | 1 | 1 | 4 |
| hsa-miR-330-5p | BTN2A2 | 1 | 1 | 1 | 1 | 4 |
| hsa-miR-330-5p | MYL9 | 1 | 1 | 1 | 1 | 4 |
| hsa-miR-330-5p | SPON1 | 1 | 1 | 1 | 1 | 4 |
| hsa-miR-330-5p | ZER1 | 1 | 1 | 1 | 1 | 4 |
| hsa-miR-330-5p | MAP3K7IP1 | 1 | 1 | 1 | 1 | 4 |
| hsa-miR-330-5p | SEMA4D | 1 | 1 | 1 | 1 | 4 |
| hsa-miR-330-5p | CAMKK2 | 1 | 1 | 1 | 1 | 4 |
| hsa-miR-330-5p | DLL3 | 1 | 1 | 1 | 1 | 4 |
| hsa-miR-330-5p | RAI1 | 1 | 1 | 1 | 1 | 4 |
| hsa-miR-330-5p | TOB2 | 1 | 1 | 1 | 1 | 4 |
| hsa-miR-330-5p | FTCD | 1 | 1 | 1 | 1 | 4 |
| hsa-miR-330-5p | USP20 | 1 | 1 | 1 | 1 | 4 |
| hsa-miR-330-5p | HNRPUL1 | 1 | 1 | 1 | 1 | 4 |
| hsa-miR-330-5p | ZWINT | 1 | 1 | 1 | 1 | 4 |
| hsa-miR-330-5p | LDB3 | 1 | 1 | 1 | 1 | 4 |
| hsa-miR-330-5p | RASSF1 | 1 | 1 | 1 | 1 | 4 |
| hsa-miR-330-5p | CEP250 | 1 | 1 | 1 | 1 | 4 |
| hsa-miR-330-5p | CHML | 1 | 1 | 1 | 1 | 4 |
| hsa-miR-330-5p | SEC63 | 1 | 1 | 1 | 1 | 4 |
| hsa-miR-330-5p | C1QTNF6 | 1 | 1 | 1 | 1 | 4 |
| hsa-miR-330-5p | RASGRP4 | 1 | 1 | 1 | 1 | 4 |
| hsa-miR-330-5p | CKM | 1 | 1 | 1 | 1 | 4 |
| hsa-miR-330-5p | FOXP4 | 1 | 1 | 1 | 1 | 4 |
| hsa-miR-330-5p | GRIN3A | 1 | 1 | 1 | 1 | 4 |
| hsa-miR-330-5p | ZNF641 | 1 | 1 | 1 | 1 | 4 |
| hsa-miR-330-5p | C15orf27 | 1 | 1 | 1 | 1 | 4 |
| hsa-miR-330-5p | CCR7 | 1 | 1 | 1 | 1 | 4 |
| hsa-miR-330-5p | TBC1D16 | 1 | 1 | 1 | 1 | 4 |
| hsa-miR-330-5p | C18orf51 | 1 | 1 | 1 | 1 | 4 |
| hsa-miR-330-5p | TDRD10 | 1 | 1 | 1 | 1 | 4 |
| hsa-miR-330-5p | KLHDC7A | 1 | 1 | 1 | 1 | 4 |
| hsa-miR-330-5p | COL4A1 | 1 | 1 | 1 | 1 | 4 |
| hsa-miR-330-5p | COL4A3 | 1 | 1 | 1 | 1 | 4 |
| hsa-miR-330-5p | COL4A4 | 1 | 1 | 1 | 1 | 4 |
| hsa-miR-330-5p | COL6A2 | 1 | 1 | 1 | 1 | 4 |
| hsa-miR-330-5p | UGT3A1 | 1 | 1 | 1 | 1 | 4 |
| hsa-miR-330-5p | EMID2 | 1 | 1 | 1 | 1 | 4 |
| hsa-miR-330-5p | CXorf41 | 1 | 1 | 1 | 1 | 4 |
| hsa-miR-330-5p | HDX | 1 | 1 | 1 | 1 | 4 |
| hsa-miR-330-5p | MYO3B | 1 | 1 | 1 | 1 | 4 |
| hsa-miR-330-5p | XRRA1 | 1 | 1 | 1 | 1 | 4 |
| hsa-miR-330-5p | BEST3 | 1 | 1 | 1 | 1 | 4 |
| hsa-miR-330-5p | A2ML1 | 1 | 1 | 1 | 1 | 4 |
| hsa-miR-330-5p | FAM109A | 1 | 1 | 1 | 1 | 4 |
| hsa-miR-330-5p | SLC25A10 | 1 | 1 | 1 | 1 | 4 |
| hsa-miR-330-5p | APCDD1 | 1 | 1 | 1 | 1 | 4 |
| hsa-miR-330-5p | RC3H1 | 1 | 1 | 1 | 1 | 4 |
| hsa-miR-330-5p | FAM71A | 1 | 1 | 1 | 1 | 4 |
| hsa-miR-330-5p | ZXDB | 1 | 1 | 1 | 1 | 4 |
| hsa-miR-330-5p | DACH1 | 1 | 1 | 1 | 1 | 4 |
| hsa-miR-330-5p | AP1B1 | 1 | 1 | 1 | 1 | 4 |
| hsa-miR-330-5p | NAGS | 1 | 1 | 1 | 1 | 4 |
| hsa-miR-330-5p | FAM134C | 1 | 1 | 1 | 1 | 4 |
| hsa-miR-330-5p | C1orf55 | 1 | 1 | 1 | 1 | 4 |
| hsa-miR-330-5p | C1orf71 | 1 | 1 | 1 | 1 | 4 |
| hsa-miR-330-5p | CABP7 | 1 | 1 | 1 | 1 | 4 |
| hsa-miR-330-5p | ZNF509 | 1 | 1 | 1 | 1 | 4 |
| hsa-miR-330-5p | DLST | 1 | 1 | 1 | 1 | 4 |
| hsa-miR-330-5p | DNM2 | 1 | 1 | 1 | 1 | 4 |
| hsa-miR-330-5p | TRDMT1 | 1 | 1 | 1 | 1 | 4 |
| hsa-miR-330-5p | DOCK3 | 1 | 1 | 1 | 1 | 4 |
| hsa-miR-330-5p | ATN1 | 1 | 1 | 1 | 1 | 4 |
| hsa-miR-330-5p | CELSR3 | 1 | 1 | 1 | 1 | 4 |
| hsa-miR-330-5p | SLFNL1 | 1 | 1 | 1 | 1 | 4 |
| hsa-miR-330-5p | LOC201175 | 1 | 1 | 1 | 1 | 4 |
| hsa-miR-330-5p | EPB41L1 | 1 | 1 | 1 | 1 | 4 |
| hsa-miR-330-5p | EPB41L2 | 1 | 1 | 1 | 1 | 4 |
| hsa-miR-330-5p | CLN8 | 1 | 1 | 1 | 1 | 4 |
| hsa-miR-330-5p | KIAA2018 | 1 | 1 | 1 | 1 | 4 |
| hsa-miR-330-5p | FAT2 | 1 | 1 | 1 | 1 | 4 |
| hsa-miR-330-5p | GPR114 | 1 | 1 | 1 | 1 | 4 |
| hsa-miR-330-5p | ZNF498 | 1 | 1 | 1 | 1 | 4 |
| hsa-miR-330-5p | FGFR4 | 1 | 1 | 1 | 1 | 4 |
| hsa-miR-330-5p | RNF44 | 1 | 1 | 1 | 1 | 4 |
| hsa-miR-330-5p | VASH1 | 1 | 1 | 1 | 1 | 4 |
| hsa-miR-330-5p | ZNF510 | 1 | 1 | 1 | 1 | 4 |
| hsa-miR-330-5p | ZHX2 | 1 | 1 | 1 | 1 | 4 |
| hsa-miR-330-5p | CEP164 | 1 | 1 | 1 | 1 | 4 |
| hsa-miR-330-5p | HABP4 | 1 | 1 | 1 | 1 | 4 |
| hsa-miR-330-5p | FOXI1 | 1 | 1 | 1 | 1 | 4 |
| hsa-miR-330-5p | FOXL1 | 1 | 1 | 1 | 1 | 4 |
| hsa-miR-330-5p | TMCC1 | 1 | 1 | 1 | 1 | 4 |
| hsa-miR-330-5p | KIF21B | 1 | 1 | 1 | 1 | 4 |
| hsa-miR-330-5p | KIAA0082 | 1 | 1 | 1 | 1 | 4 |
| hsa-miR-330-5p | ATP10B | 1 | 1 | 1 | 1 | 4 |
| hsa-miR-330-5p | FLNC | 1 | 1 | 1 | 1 | 4 |
| hsa-miR-330-5p | NUP210 | 1 | 1 | 1 | 1 | 4 |
| hsa-miR-330-5p | PACS2 | 1 | 1 | 1 | 1 | 4 |
| hsa-miR-330-5p | MGRN1 | 1 | 1 | 1 | 1 | 4 |
| hsa-miR-330-5p | KIF13B | 1 | 1 | 1 | 1 | 4 |
| hsa-miR-330-5p | DPY19L1 | 1 | 1 | 1 | 1 | 4 |
| hsa-miR-330-5p | KIAA1045 | 1 | 1 | 1 | 1 | 4 |
| hsa-miR-330-5p | ANGEL1 | 1 | 1 | 1 | 1 | 4 |
| hsa-miR-330-5p | SLC7A8 | 1 | 1 | 1 | 1 | 4 |
| hsa-miR-330-5p | OTP | 1 | 1 | 1 | 1 | 4 |
| hsa-miR-330-5p | ANGPTL2 | 1 | 1 | 1 | 1 | 4 |
| hsa-miR-330-5p | DAAM2 | 1 | 1 | 1 | 1 | 4 |
| hsa-miR-330-5p | CABIN1 | 1 | 1 | 1 | 1 | 4 |
| hsa-miR-330-5p | HMHA1 | 1 | 1 | 1 | 1 | 4 |
| hsa-miR-330-5p | PSD4 | 1 | 1 | 1 | 1 | 4 |
| hsa-miR-330-5p | CDC42EP4 | 1 | 1 | 1 | 1 | 4 |
| hsa-miR-330-5p | DDX58 | 1 | 1 | 1 | 1 | 4 |
| hsa-miR-330-5p | TRIM29 | 1 | 1 | 1 | 1 | 4 |
| hsa-miR-330-5p | LYPLA3 | 1 | 1 | 1 | 1 | 4 |
| hsa-miR-330-5p | SH3BP4 | 1 | 1 | 1 | 1 | 4 |
| hsa-miR-330-5p | GABARAPL1 | 1 | 1 | 1 | 1 | 4 |
| hsa-miR-330-5p | SHPK | 1 | 1 | 1 | 1 | 4 |
| hsa-miR-330-5p | FUCA1 | 1 | 1 | 1 | 1 | 4 |
| hsa-miR-330-5p | ZNF396 | 1 | 1 | 1 | 1 | 4 |
| hsa-miR-330-5p | RASGEF1C | 1 | 1 | 1 | 1 | 4 |
| hsa-miR-330-5p | COL29A1 | 1 | 1 | 1 | 1 | 4 |
| hsa-miR-330-5p | TMEM151A | 1 | 1 | 1 | 1 | 4 |
| hsa-miR-330-5p | C15orf55 | 1 | 1 | 1 | 1 | 4 |
| hsa-miR-330-5p | IFFO | 1 | 1 | 1 | 1 | 4 |
| hsa-miR-330-5p | CLIC4 | 1 | 1 | 1 | 1 | 4 |
| hsa-miR-330-5p | GPR124 | 1 | 1 | 1 | 1 | 4 |
| hsa-miR-330-5p | SNED1 | 1 | 1 | 1 | 1 | 4 |
| hsa-miR-330-5p | LRIG1 | 1 | 1 | 1 | 1 | 4 |
| hsa-miR-330-5p | APPL1 | 1 | 1 | 1 | 1 | 4 |
| hsa-miR-330-5p | TANC2 | 1 | 1 | 1 | 1 | 4 |
| hsa-miR-330-5p | RSL1D1 | 1 | 1 | 1 | 1 | 4 |
| hsa-miR-330-5p | GATA2 | 1 | 1 | 1 | 1 | 4 |
| hsa-miR-330-5p | NR6A1 | 1 | 1 | 1 | 1 | 4 |
| hsa-miR-330-5p | C1orf107 | 1 | 1 | 1 | 1 | 4 |
| hsa-miR-330-5p | PSCD4 | 1 | 1 | 1 | 1 | 4 |
| hsa-miR-330-5p | STK36 | 1 | 1 | 1 | 1 | 4 |
| hsa-miR-330-5p | GLS2 | 1 | 1 | 1 | 1 | 4 |
| hsa-miR-330-5p | CSDC2 | 1 | 1 | 1 | 1 | 4 |
| hsa-miR-330-5p | MOCS3 | 1 | 1 | 1 | 1 | 4 |
| hsa-miR-330-5p | SGSM3 | 1 | 1 | 1 | 1 | 4 |
| hsa-miR-330-5p | GLI2 | 1 | 1 | 1 | 1 | 4 |
| hsa-miR-330-5p | SLC9A9 | 1 | 1 | 1 | 1 | 4 |
| hsa-miR-330-5p | GPR35 | 1 | 1 | 1 | 1 | 4 |
| hsa-miR-330-5p | XKR6 | 1 | 1 | 1 | 1 | 4 |
| hsa-miR-330-5p | CRB2 | 1 | 1 | 1 | 1 | 4 |
| hsa-miR-330-5p | TUSC5 | 1 | 1 | 1 | 1 | 4 |
| hsa-miR-330-5p | KLF15 | 1 | 1 | 1 | 1 | 4 |
| hsa-miR-330-5p | ANKRD11 | 1 | 1 | 1 | 1 | 4 |
| hsa-miR-330-5p | GSTM5 | 1 | 1 | 1 | 1 | 4 |
| hsa-miR-330-5p | BRF1 | 1 | 1 | 1 | 1 | 4 |
| hsa-miR-330-5p | HCFC1 | 1 | 1 | 1 | 1 | 4 |
| hsa-miR-330-5p | HLA-DQB1 | 1 | 1 | 1 | 1 | 4 |
| hsa-miR-330-5p | TNC | 1 | 1 | 1 | 1 | 4 |
| hsa-miR-330-5p | LOC338809 | 1 | 1 | 1 | 1 | 4 |
| hsa-miR-330-5p | KY | 1 | 1 | 1 | 1 | 4 |
| hsa-miR-330-5p | PLCXD3 | 1 | 1 | 1 | 1 | 4 |
| hsa-miR-330-5p | AMIGO2 | 1 | 1 | 1 | 1 | 4 |
| hsa-miR-330-5p | IGF1R | 1 | 1 | 1 | 1 | 4 |
| hsa-miR-330-5p | RBPMS2 | 1 | 1 | 1 | 1 | 4 |
| hsa-miR-330-5p | IGFBP4 | 1 | 1 | 1 | 1 | 4 |
| hsa-miR-330-5p | WDR86 | 1 | 1 | 1 | 1 | 4 |
| hsa-miR-330-5p | IL16 | 1 | 1 | 1 | 1 | 4 |
| hsa-miR-330-5p | AQP6 | 1 | 1 | 1 | 1 | 4 |
| hsa-miR-330-5p | ITGA2 | 1 | 1 | 1 | 1 | 4 |
| hsa-miR-330-5p | ITGA4 | 1 | 1 | 1 | 1 | 4 |
| hsa-miR-330-5p | KCNMA1 | 1 | 1 | 1 | 1 | 4 |
| hsa-miR-330-5p | RHOA | 1 | 1 | 1 | 1 | 4 |
| hsa-miR-330-5p | LOC388272 | 1 | 1 | 1 | 1 | 4 |
| hsa-miR-330-5p | ARHGAP1 | 1 | 1 | 1 | 1 | 4 |
| hsa-miR-330-5p | LGALS8 | 1 | 1 | 1 | 1 | 4 |
| hsa-miR-330-5p | LIF | 1 | 1 | 1 | 1 | 4 |
| hsa-miR-330-5p | SNX19 | 1 | 1 | 1 | 1 | 4 |
| hsa-miR-330-5p | SH2D5 | 1 | 1 | 1 | 1 | 4 |
| hsa-miR-330-5p | SOHLH1 | 1 | 1 | 1 | 1 | 4 |
| hsa-miR-330-5p | MC2R | 1 | 1 | 1 | 1 | 4 |
| hsa-miR-330-5p | MCM2 | 1 | 1 | 1 | 1 | 4 |
| hsa-miR-330-5p | ADAM11 | 1 | 1 | 1 | 1 | 4 |
| hsa-miR-330-5p | MDFI | 1 | 1 | 1 | 1 | 4 |
| hsa-miR-330-5p | MLLT6 | 1 | 1 | 1 | 1 | 4 |
| hsa-miR-330-5p | MMP2 | 1 | 1 | 1 | 1 | 4 |
| hsa-miR-330-5p | NEFH | 1 | 1 | 1 | 1 | 4 |
| hsa-miR-330-5p | NFX1 | 1 | 1 | 1 | 1 | 4 |
| hsa-miR-330-5p | NOTCH2 | 1 | 1 | 1 | 1 | 4 |
| hsa-miR-330-5p | NP | 1 | 1 | 1 | 1 | 4 |
| hsa-miR-330-5p | NTRK3 | 1 | 1 | 1 | 1 | 4 |
| hsa-miR-330-5p | NUMA1 | 1 | 1 | 1 | 1 | 4 |
| hsa-miR-330-5p | NUP98 | 1 | 1 | 1 | 1 | 4 |
| hsa-miR-330-5p | ATP2B4 | 1 | 1 | 1 | 1 | 4 |
| hsa-miR-330-5p | RNF165 | 1 | 1 | 1 | 1 | 4 |
| hsa-miR-330-5p | RDH8 | 1 | 1 | 1 | 1 | 4 |
| hsa-miR-330-5p | F11R | 1 | 1 | 1 | 1 | 4 |
| hsa-miR-330-5p | PCDH1 | 1 | 1 | 1 | 1 | 4 |
| hsa-miR-330-5p | TMED5 | 1 | 1 | 1 | 1 | 4 |
| hsa-miR-330-5p | SERPINA5 | 1 | 1 | 1 | 1 | 4 |
| hsa-miR-330-5p | MLXIPL | 1 | 1 | 1 | 1 | 4 |
| hsa-miR-330-5p | GLTP | 1 | 1 | 1 | 1 | 4 |
| hsa-miR-330-5p | C3orf19 | 1 | 1 | 1 | 1 | 4 |
| hsa-miR-330-5p | GMIP | 1 | 1 | 1 | 1 | 4 |
| hsa-miR-330-5p | HEMK1 | 1 | 1 | 1 | 1 | 4 |
| hsa-miR-330-5p | PDGFRA | 1 | 1 | 1 | 1 | 4 |
| hsa-miR-330-5p | ASB1 | 1 | 1 | 1 | 1 | 4 |
| hsa-miR-330-5p | PGF | 1 | 1 | 1 | 1 | 4 |
| hsa-miR-330-5p | PGK1 | 1 | 1 | 1 | 1 | 4 |
| hsa-miR-330-5p | PIGA | 1 | 1 | 1 | 1 | 4 |
| hsa-miR-330-5p | PLA2G5 | 1 | 1 | 1 | 1 | 4 |
| hsa-miR-330-5p | PLD1 | 1 | 1 | 1 | 1 | 4 |
| hsa-miR-330-5p | SERPINF2 | 1 | 1 | 1 | 1 | 4 |
| hsa-miR-330-5p | ATP7B | 1 | 1 | 1 | 1 | 4 |
| hsa-miR-330-5p | BRWD1 | 1 | 1 | 1 | 1 | 4 |
| hsa-miR-330-5p | DDIT4 | 1 | 1 | 1 | 1 | 4 |
| hsa-miR-330-5p | KIF1A | 1 | 1 | 1 | 1 | 4 |
| hsa-miR-330-5p | PPP1R12C | 1 | 1 | 1 | 1 | 4 |
| hsa-miR-330-5p | BSPRY | 1 | 1 | 1 | 1 | 4 |
| hsa-miR-330-5p | CC2D1A | 1 | 1 | 1 | 1 | 4 |
| hsa-miR-330-5p | SSH3 | 1 | 1 | 1 | 1 | 4 |
| hsa-miR-330-5p | FLJ20699 | 1 | 1 | 1 | 1 | 4 |
| hsa-miR-330-5p | PDPR | 1 | 1 | 1 | 1 | 4 |
| hsa-miR-330-5p | ANKZF1 | 1 | 1 | 1 | 1 | 4 |
| hsa-miR-330-5p | MRPS10 | 1 | 1 | 1 | 1 | 4 |
| hsa-miR-330-5p | VPS13D | 1 | 1 | 1 | 1 | 4 |
| hsa-miR-330-5p | KLHDC8A | 1 | 1 | 1 | 1 | 4 |
| hsa-miR-330-5p | HJURP | 1 | 1 | 1 | 1 | 4 |
| hsa-miR-330-5p | TRPV6 | 1 | 1 | 1 | 1 | 4 |
| hsa-miR-330-5p | SMPD3 | 1 | 1 | 1 | 1 | 4 |
| hsa-miR-330-5p | ZNF821 | 1 | 1 | 1 | 1 | 4 |
| hsa-miR-330-5p | PRIM2 | 1 | 1 | 1 | 1 | 4 |
| hsa-miR-330-5p | PCDHGB7 | 1 | 1 | 1 | 1 | 4 |
| hsa-miR-330-5p | PCDHGA11 | 1 | 1 | 1 | 1 | 4 |
| hsa-miR-330-5p | PCDHGA10 | 1 | 1 | 1 | 1 | 4 |
| hsa-miR-330-5p | SEMA3G | 1 | 1 | 1 | 1 | 4 |
| hsa-miR-330-5p | OTUD7B | 1 | 1 | 1 | 1 | 4 |
| hsa-miR-330-5p | CYSLTR2 | 1 | 1 | 1 | 1 | 4 |
| hsa-miR-330-5p | NPAL3 | 1 | 1 | 1 | 1 | 4 |
| hsa-miR-330-5p | ATP10A | 1 | 1 | 1 | 1 | 4 |
| hsa-miR-330-5p | TTC7A | 1 | 1 | 1 | 1 | 4 |
| hsa-miR-330-5p | PTBP1 | 1 | 1 | 1 | 1 | 4 |
| hsa-miR-330-5p | TBC1D24 | 1 | 1 | 1 | 1 | 4 |
| hsa-miR-330-5p | PLEKHG1 | 1 | 1 | 1 | 1 | 4 |
| hsa-miR-330-5p | KIAA1303 | 1 | 1 | 1 | 1 | 4 |
| hsa-miR-330-5p | KIAA1407 | 1 | 1 | 1 | 1 | 4 |
| hsa-miR-330-5p | KCNT1 | 1 | 1 | 1 | 1 | 4 |
| hsa-miR-330-5p | CRAMP1L | 1 | 1 | 1 | 1 | 4 |
| hsa-miR-330-5p | SYT13 | 1 | 1 | 1 | 1 | 4 |
| hsa-miR-330-5p | KIAA1462 | 1 | 1 | 1 | 1 | 4 |
| hsa-miR-330-5p | GATAD1 | 1 | 1 | 1 | 1 | 4 |
| hsa-miR-330-5p | PTPN9 | 1 | 1 | 1 | 1 | 4 |
| hsa-miR-330-5p | G6PC2 | 1 | 1 | 1 | 1 | 4 |
| hsa-miR-330-5p | PEX19 | 1 | 1 | 1 | 1 | 4 |
| hsa-miR-330-5p | RAC2 | 1 | 1 | 1 | 1 | 4 |
| hsa-miR-330-5p | REN | 1 | 1 | 1 | 1 | 4 |
| hsa-miR-330-5p | ELAC2 | 1 | 1 | 1 | 1 | 4 |
| hsa-miR-330-5p | RP5-1077B9.4 | 1 | 1 | 1 | 1 | 4 |
| hsa-miR-330-5p | ZFAND3 | 1 | 1 | 1 | 1 | 4 |
| hsa-miR-330-5p | SORT1 | 1 | 1 | 1 | 1 | 4 |
| hsa-miR-330-5p | SALL2 | 1 | 1 | 1 | 1 | 4 |
| hsa-miR-330-5p | SELP | 1 | 1 | 1 | 1 | 4 |
| hsa-miR-330-5p | ZFYVE20 | 1 | 1 | 1 | 1 | 4 |
| hsa-miR-330-5p | PDLIM2 | 1 | 1 | 1 | 1 | 4 |
| hsa-miR-330-5p | HS1BP3 | 1 | 1 | 1 | 1 | 4 |
| hsa-miR-330-5p | LOC643641 | 1 | 1 | 1 | 1 | 4 |
| hsa-miR-330-5p | SUDS3 | 1 | 1 | 1 | 1 | 4 |
| hsa-miR-330-5p | NARFL | 1 | 1 | 1 | 1 | 4 |
| hsa-miR-330-5p | C16orf58 | 1 | 1 | 1 | 1 | 4 |
| hsa-miR-330-5p | TNS3 | 1 | 1 | 1 | 1 | 4 |
| hsa-miR-330-5p | ST3GAL4 | 1 | 1 | 1 | 1 | 4 |
| hsa-miR-330-5p | JMJD4 | 1 | 1 | 1 | 1 | 4 |
| hsa-miR-330-5p | SLC6A1 | 1 | 1 | 1 | 1 | 4 |
| hsa-miR-330-5p | SLC6A2 | 1 | 1 | 1 | 1 | 4 |
| hsa-miR-330-5p | SLC13A1 | 1 | 1 | 1 | 1 | 4 |
| hsa-miR-330-5p | SLCO2A1 | 1 | 1 | 1 | 1 | 4 |
| hsa-miR-330-5p | SP4 | 1 | 1 | 1 | 1 | 4 |
| hsa-miR-330-5p | SPG7 | 1 | 1 | 1 | 1 | 4 |
| hsa-miR-330-5p | SREBF1 | 1 | 1 | 1 | 1 | 4 |
| hsa-miR-330-5p | STAT1 | 1 | 1 | 1 | 1 | 4 |
| hsa-miR-330-5p | SYP | 1 | 1 | 1 | 1 | 4 |
| hsa-miR-330-5p | TACC1 | 1 | 1 | 1 | 1 | 4 |
| hsa-miR-330-5p | TBCD | 1 | 1 | 1 | 1 | 4 |
| hsa-miR-330-5p | TEAD3 | 1 | 1 | 1 | 1 | 4 |
| hsa-miR-330-5p | DAGLA | 1 | 1 | 1 | 1 | 4 |
| hsa-miR-330-5p | ZNF10 | 1 | 1 | 1 | 1 | 4 |
| hsa-miR-330-5p | CHAC1 | 1 | 1 | 1 | 1 | 4 |
| hsa-miR-330-5p | C1orf89 | 1 | 1 | 1 | 1 | 4 |
| hsa-miR-330-5p | NKAIN1 | 1 | 1 | 1 | 1 | 4 |
| hsa-miR-330-5p | CHPF | 1 | 1 | 1 | 1 | 4 |
| hsa-miR-330-5p | FLJ11506 | 1 | 1 | 1 | 1 | 4 |
| hsa-miR-330-5p | FBXO31 | 1 | 1 | 1 | 1 | 4 |
| hsa-miR-330-5p | EHMT1 | 1 | 1 | 1 | 1 | 4 |
| hsa-miR-330-5p | BAALC | 1 | 1 | 1 | 1 | 4 |
| hsa-miR-330-5p | ADCK4 | 1 | 1 | 1 | 1 | 4 |
| hsa-miR-330-5p | PHF17 | 1 | 1 | 1 | 1 | 4 |
| hsa-miR-330-5p | GPR177 | 1 | 1 | 1 | 1 | 4 |
| hsa-miR-330-5p | GRHL2 | 1 | 1 | 1 | 1 | 4 |
| hsa-miR-330-5p | SVEP1 | 1 | 1 | 1 | 1 | 4 |
| hsa-miR-330-5p | PGAP1 | 1 | 1 | 1 | 1 | 4 |
| hsa-miR-330-5p | EDC3 | 1 | 1 | 1 | 1 | 4 |
| hsa-miR-330-5p | SPSB1 | 1 | 1 | 1 | 1 | 4 |
| hsa-miR-330-5p | FOSL1 | 1 | 1 | 1 | 1 | 4 |
| hsa-miR-330-5p | CUL5 | 1 | 1 | 1 | 1 | 4 |
| hsa-miR-330-5p | STARD5 | 1 | 1 | 1 | 1 | 4 |
| hsa-miR-330-5p | RSHL1 | 1 | 1 | 1 | 1 | 4 |
| hsa-miR-330-5p | PVRL4 | 1 | 1 | 1 | 1 | 4 |
| hsa-miR-330-5p | TIGD6 | 1 | 1 | 1 | 1 | 4 |
| hsa-miR-330-5p | ADAMTS10 | 1 | 1 | 1 | 1 | 4 |
| hsa-miR-330-5p | LZTR1 | 1 | 1 | 1 | 1 | 4 |
| hsa-miR-330-5p | SYN3 | 1 | 1 | 1 | 1 | 4 |
| hsa-miR-330-5p | JARID1C | 1 | 1 | 1 | 1 | 4 |
| hsa-miR-330-5p | SNN | 1 | 1 | 1 | 1 | 4 |
| hsa-miR-330-5p | C9orf58 | 1 | 1 | 1 | 1 | 4 |
| hsa-miR-330-5p | RIOK1 | 1 | 1 | 1 | 1 | 4 |
| hsa-miR-330-5p | TFAP2D | 1 | 1 | 1 | 1 | 4 |
| hsa-miR-330-5p | KCTD10 | 1 | 1 | 1 | 1 | 4 |
| hsa-miR-330-5p | C5orf21 | 1 | 1 | 1 | 1 | 4 |
| hsa-miR-330-5p | C16orf48 | 1 | 1 | 1 | 1 | 4 |
| hsa-miR-330-5p | SLA2 | 1 | 1 | 1 | 1 | 4 |
| hsa-miR-330-5p | CCDC135 | 1 | 1 | 1 | 1 | 4 |
| hsa-miR-330-5p | POLDIP3 | 1 | 1 | 1 | 1 | 4 |
| hsa-miR-330-5p | NR0B2 | 1 | 1 | 1 | 1 | 4 |
| hsa-miR-330-5p | RAB11FIP4 | 1 | 1 | 1 | 1 | 4 |
| hsa-miR-330-5p | SPIRE2 | 1 | 1 | 1 | 1 | 4 |
| hsa-miR-330-5p | GHDC | 1 | 1 | 1 | 1 | 4 |
| hsa-miR-330-5p | KRBA1 | 1 | 1 | 1 | 1 | 4 |
| hsa-miR-330-5p | GLIS2 | 1 | 1 | 1 | 1 | 4 |
| hsa-miR-330-5p | MYO18B | 1 | 1 | 1 | 1 | 4 |
| hsa-miR-330-5p | CNDP1 | 1 | 1 | 1 | 1 | 4 |
| hsa-miR-330-5p | PPFIBP2 | 1 | 1 | 1 | 1 | 4 |
| hsa-miR-330-5p | MICALCL | 1 | 1 | 1 | 1 | 4 |
| hsa-miR-330-5p | JUB | 1 | 1 | 1 | 1 | 4 |
| hsa-miR-330-5p | FAM83A | 1 | 1 | 1 | 1 | 4 |
| hsa-miR-330-5p | GAS7 | 1 | 1 | 1 | 1 | 4 |
| hsa-miR-330-5p | LL22NC03-75B3.6 | 1 | 1 | 1 | 1 | 4 |
| hsa-miR-330-5p | SYDE1 | 1 | 1 | 1 | 1 | 4 |
| hsa-miR-330-5p | SLC45A3 | 1 | 1 | 1 | 1 | 4 |
| hsa-miR-330-5p | KNDC1 | 1 | 1 | 1 | 1 | 4 |
| hsa-miR-330-5p | KIAA1755 | 1 | 1 | 1 | 1 | 4 |
| hsa-miR-330-5p | AOC3 | 1 | 1 | 1 | 1 | 4 |
| hsa-miR-330-5p | STX16 | 1 | 1 | 1 | 1 | 4 |
| hsa-miR-330-5p | ADAM15 | 1 | 1 | 1 | 1 | 4 |
| hsa-miR-330-5p | ACTN2 | 1 | 1 | 1 | 1 | 4 |
| hsa-miR-330-5p | CACNA1H | 1 | 1 | 1 | 1 | 4 |
| hsa-miR-330-5p | C9orf140 | 1 | 1 | 1 | 1 | 4 |
| hsa-miR-330-5p | EMILIN3 | 1 | 1 | 1 | 1 | 4 |
| hsa-miR-330-5p | ZNF551 | 1 | 1 | 1 | 1 | 4 |
| hsa-miR-330-5p | CCDC97 | 1 | 1 | 1 | 1 | 4 |
| hsa-miR-330-5p | BMF | 1 | 1 | 1 | 1 | 4 |
| hsa-miR-330-5p | STARD13 | 1 | 1 | 1 | 1 | 4 |
| hsa-miR-330-5p | SPOCD1 | 1 | 1 | 1 | 1 | 4 |
| hsa-miR-330-5p | KIFC2 | 1 | 1 | 1 | 1 | 4 |
| hsa-miR-330-5p | MTMR7 | 1 | 1 | 1 | 1 | 4 |
| hsa-miR-330-5p | L3MBTL4 | 1 | 1 | 1 | 1 | 4 |
| hsa-miR-330-5p | RABEP1 | 1 | 1 | 1 | 1 | 4 |
| hsa-miR-330-5p | SYNGR1 | 1 | 1 | 1 | 1 | 4 |
| hsa-miR-330-5p | SLC28A1 | 1 | 1 | 1 | 1 | 4 |
| hsa-miR-330-5p | SYT12 | 1 | 1 | 1 | 1 | 4 |
| hsa-miR-330-5p | ADAMTSL1 | 1 | 1 | 1 | 1 | 4 |
| hsa-miR-330-5p | SOCS6 | 1 | 1 | 1 | 1 | 4 |
| hsa-miR-330-5p | STOML1 | 1 | 1 | 1 | 1 | 4 |
| hsa-miR-330-5p | SFXN5 | 1 | 1 | 1 | 1 | 4 |
| hsa-miR-330-5p | STXBP5L | 1 | 1 | 1 | 1 | 4 |
| hsa-miR-330-5p | ENTPD2 | 1 | 1 | 1 | 1 | 4 |
| hsa-miR-330-5p | IER2 | 1 | 1 | 1 | 1 | 4 |
| hsa-miR-330-5p | AATK | 1 | 1 | 1 | 1 | 4 |
| hsa-miR-330-5p | CROCC | 1 | 1 | 1 | 1 | 4 |
| hsa-miR-330-5p | SAPS2 | 1 | 1 | 1 | 1 | 4 |
| hsa-miR-330-5p | SNPH | 1 | 1 | 1 | 1 | 4 |
| hsa-miR-330-5p | MLL4 | 1 | 1 | 1 | 1 | 4 |
| hsa-miR-330-5p | RAPGEF5 | 1 | 1 | 1 | 1 | 4 |
| hsa-miR-330-5p | RNF40 | 1 | 1 | 1 | 1 | 4 |
| hsa-miR-330-5p | KIAA0753 | 1 | 1 | 1 | 1 | 4 |
| hsa-miR-330-5p | MAFB | 1 | 1 | 1 | 1 | 4 |
